# KLF4 is a key determinant in the development and progression of cerebral cavernous malformations

**DOI:** 10.15252/emmm.201505433

**Published:** 2015-11-26

**Authors:** Roberto Cuttano, Noemi Rudini, Luca Bravi, Monica Corada, Costanza Giampietro, Eleanna Papa, Marco Francesco Morini, Luigi Maddaluno, Nicolas Baeyens, Ralf H Adams, Mukesh K Jain, Gary K Owens, Martin Schwartz, Maria Grazia Lampugnani, Elisabetta Dejana

**Affiliations:** ^1^ IFOMthe FIRC Institute of Molecular OncologyMilanItaly; ^2^ Department of BiosciencesUniversity of MilanMilanItaly; ^3^ on leave of absence at Department of NeurologyLaboratory for Molecular Neuro‐OncologyUniversity Hospital ZurichZurichSwitzerland; ^4^ on leave of absence at Department of BiomedicineUniversity of BaselBaselSwitzerland; ^5^ on leave of absence at Institute of Molecular Health SciencesETH ZurichZurichSwitzerland; ^6^ Yale Cardiovascular Research CenterNew HavenCTUSA; ^7^ Department of Tissue MorphogenesisFaculty of MedicineMax Planck Institute for Molecular BiomedicineUniversity of MünsterMünsterGermany; ^8^ Case Cardiovascular Research InstituteClevelandOHUSA; ^9^ Harrington Heart & Vascular InstituteClevelandOHUSA; ^10^ Department of MedicineUniversity Hospitals Case Medical CenterClevelandOHUSA; ^11^ Case Western Reserve University School of MedicineUniversity Hospitals Case Medical CenterClevelandOHUSA; ^12^ Robert M. Berne Cardiovascular Research CenterUniversity of Virginia School of MedicineCharlottesvilleVirginiaUSA; ^13^ Mario Negri Institute of Pharmacological ResearchMilanItaly; ^14^ Department of ImmunologyGenetics and PathologyUppsala UniversityUppsalaSweden; ^15^ Department of Oncology and OncohematologyUniversity of MilanMilanItaly

**Keywords:** CCM, EndMT, endothelial cells, KLF4, TGFβ‐BMP, Cardiovascular System, Genetics, Gene Therapy & Genetic Disease, Vascular Biology & Angiogenesis

## Abstract

Cerebral cavernous malformations (CCMs) are vascular malformations located within the central nervous system often resulting in cerebral hemorrhage. Pharmacological treatment is needed, since current therapy is limited to neurosurgery. Familial CCM is caused by loss‐of‐function mutations in any of *Ccm1*,* Ccm2,* and *Ccm3* genes. CCM cavernomas are lined by endothelial cells (ECs) undergoing endothelial‐to‐mesenchymal transition (EndMT). This switch in phenotype is due to the activation of the transforming growth factor beta/bone morphogenetic protein (TGFβ/BMP) signaling. However, the mechanism linking *Ccm* gene inactivation and TGFβ/BMP‐dependent EndMT remains undefined. Here, we report that *Ccm1* ablation leads to the activation of a MEKK3‐MEK5‐ERK5‐MEF2 signaling axis that induces a strong increase in Kruppel‐like factor 4 (KLF4) in ECs *in vivo*. KLF4 transcriptional activity is responsible for the EndMT occurring in CCM1‐null ECs. KLF4 promotes TGFβ/BMP signaling through the production of BMP6. Importantly, in endothelial‐specific *Ccm1* and *Klf4* double knockout mice, we observe a strong reduction in the development of CCM and mouse mortality. Our data unveil KLF4 as a therapeutic target for CCM.

## Introduction

Cerebral cavernous malformation (CCM) is a vascular dysplasia with a prevalence of 0.5% of the human population that affects almost exclusively the venous microvasculature of the central nervous system and of the retina (Labauge *et al*, 2007; Riant *et al*, 2010). Cavernomas are formed by mulberry‐like enlarged and irregular blood vessels that often lack the support of mural cells and tend to bleed (Tomlinson *et al*, 1994; Maraire & Awad, 1995; Clatterbuck *et al*, 2001) causing several neurological symptoms including headache, seizure, paralysis, and hemorrhagic stroke (Cavalcanti *et al*, 2012). Location and number of lesions determine the severity of this disorder (Moriarity *et al*, 1999). To date, the only therapy available is neurosurgery (Rigamonti *et al*, 1988; Li & Whitehead, 2010) that, however, is frequently hazardous. CCM occurs as sporadic (80% of the cases) or familial (20% of the cases) form with high penetrance. Familial CCM is an autosomic dominant genetic disorder caused by loss‐of‐function mutations in anyone of three genes, namely *Ccm1* (*Krit1*), *Ccm2* (*Osm*), and *Ccm3* (*Pdcd10*) (Laberge‐le Couteulx *et al*, 1999; Bergametti *et al*, 2005; Labauge *et al*, 2007; Riant *et al*, 2013).

Similarly to patients, in murine models the vascular phenotype can be faithfully reproduced by endothelium‐specific loss‐of‐function mutations of anyone of these three *Ccm* genes (Liebner *et al*, 2008; Boulday *et al*, 2011; Chan *et al*, 2011; McDonald *et al*, 2011; Maddaluno *et al*, 2013; Mleynek *et al*, 2014). CCM tripartite cytoplasmic complex controls barrier functions both by inhibiting the small GTPase RhoA (Whitehead *et al*, 2009; Borikova *et al*, 2010; Stockton *et al*, 2010) and by acting as an effector of the small GTPase Ras‐related protein 1 (Rap1) at the cell‐to‐cell adherens junctions (AJ) in the endothelium (Beraud‐Dufour *et al*, 2007; Glading *et al*, 2007; Glading & Ginsberg, 2010; Fisher & Boggon, 2013). Recently, we extended these observations showing, both *in vitro* and *in vivo*, that in the absence of CCM endothelial AJ are dismantled (Lampugnani *et al*, 2010; Boulday *et al*, 2011; Maddaluno *et al*, 2013) and endothelial cells (ECs) lining the cavernomas undergo endothelial‐to‐mesenchymal transition (EndMT) (Maddaluno *et al*, 2013) induced by a strong activation of the phospho‐SMAD‐dependent signaling pathway due to an increased endogenous production of the BMP6 ligand. During EndMT, CCM‐null ECs reduce the expression of specific endothelial markers (such as Vascular‐Endothelial (VE)‐CADHERIN and CLAUDIN5), acquire typical mesenchymal and stemness‐associated factors (including Inhibitor of Differentiation 1 ID1, Fibroblast‐Specific Protein 1 FSP1, Stem Cell Antigen1 SCA1, and Kruppel‐like factor 4 KLF4), and show increase in proliferation and migration (James *et al*, 2010; Liang *et al*, 2011; Medici & Kalluri, 2012). TGFβ/BMP‐driven EndMT as well as MAPK‐dependent EndMT is implicated in several pathological conditions (Zeisberg *et al*, 2007; Kitao *et al*, 2009; Medici *et al*, 2010; Chen *et al*, 2012; Garcia *et al*, 2012), and key molecules controlling this biological process are under investigation.

KLF4, a zinc finger transcription factor of the Kruppel‐like factor family (KLF) (Garrett‐Sinha *et al*, 1996; Shields *et al*, 1996), is strongly upregulated in both the lesions and the pseudo‐normal vasculature of endothelial‐specific CCM1 KO mice (Maddaluno *et al*, 2013). KLF4 regulates different vascular functions such as angiogenesis (Ohnesorge *et al*, 2010; Hale *et al*, 2014), vascular tone and permeability (Cowan *et al*, 2010; Shatat *et al*, 2014), coagulation and inflammatory reactions (Hamik *et al*, 2007; Zhou *et al*, 2012).

In the present study, we demonstrate that the genetic inactivation of *Klf4* blocks the development and progression of CCM lesions and almost abrogates the mouse mortality due to brain hemorrhage. Furthermore, we proved that abrogation of *Ccm1* in brain ECs activates extracellular signal‐regulated kinase 5 (ERK5) through the MEKK3‐MEK5 signaling axis that, in turn, upregulates KLF4. Its transcriptional activity promotes EndMT in ECs lining the cavernomas. KLF4 acts by increasing BMP6 that promotes SMAD‐dependent EndMT which, in turn, contributes to the development of CCM vascular malformations and their hemorrhagic evolution.

Overall, this study describes a crucial mechanism through which CCM vascular malformations develop and identifies novel potential pharmacological targets to prevent the progression of this so far incurable disease.

## Results

### KLF4 is a causative factor for the development and progression of CCM lesions

**Figure 1:**
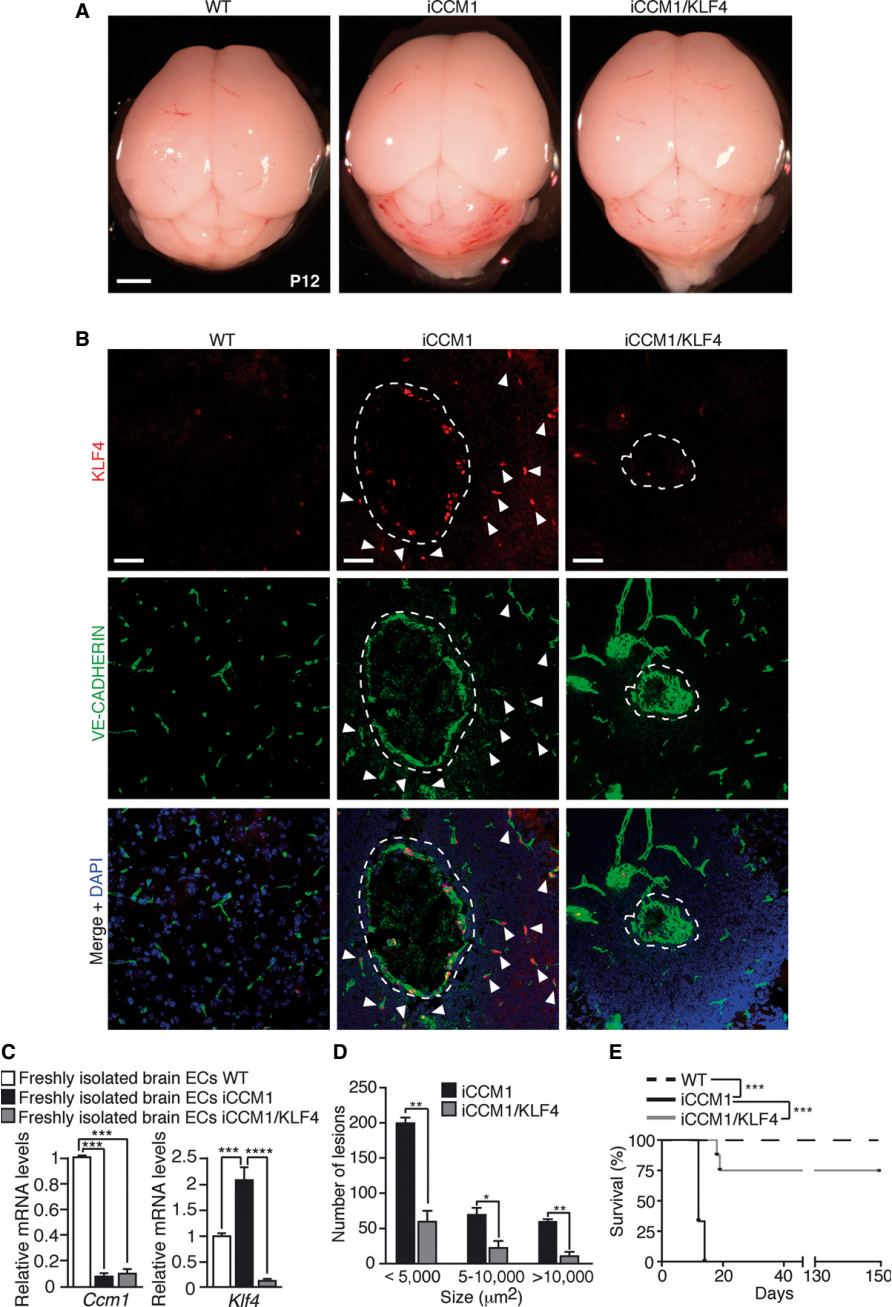
KLF4 is determinant for CCM development *in vivo* Representative images of WT, iCCM1, and iCCM1/KLF4 mouse brains at P12 (*n* = 5 for each genotype). Scale bar: 500 μm.Representative confocal analysis of VE‐CADHERIN (green) and KLF4 (red) in vascular lesions (dotted area) and pseudo‐normal cerebellar vessels (white arrowheads) of WT, iCCM1, and iCCM1/KLF4 mice (*n* = 4 in each group). VE‐CADHERIN identifies ECs; DAPI visualizes nuclei. Scale bar: 50 μm.
qRT–PCR of both *Ccm1* and *Klf4* expression performed on freshly isolated brain ECs derived by WT, iCCM1, and iCCM1/KLF4 mice at P12. Fold difference in gene expression is relative to WT mice. Data are mean ± SD (*n* = 4–5/group). A two‐tailed unpaired *t*‐test was performed. *Ccm1*: ***P = 0.0004; *Klf4*: ***P = 0.0003, ****P = 1.43E‐05.Quantification of number and size of vascular lesions in the cerebellum of iCCM1 and iCCM1/KLF4 mice at P12. Columns represent means ± SD (*n* = 3 for each genotype from 2 litters). A two‐tailed unpaired *t*‐test was performed comparing iCCM1 versus iCCM1/KLF4. < 5,000 μm^2^: **P = 0.0018; 5–1,000 μm^2^: *P = 0.0117; > 10,000 μm^2^: **P = 0.0019.Kaplan–Meier survival curve of WT, iCCM1, and iCCM1/KLF4 mice (*n* = 8 for each group). Mantel–Cox statistical test was performed: ***P < 0.0001.

**Figure EV1:**
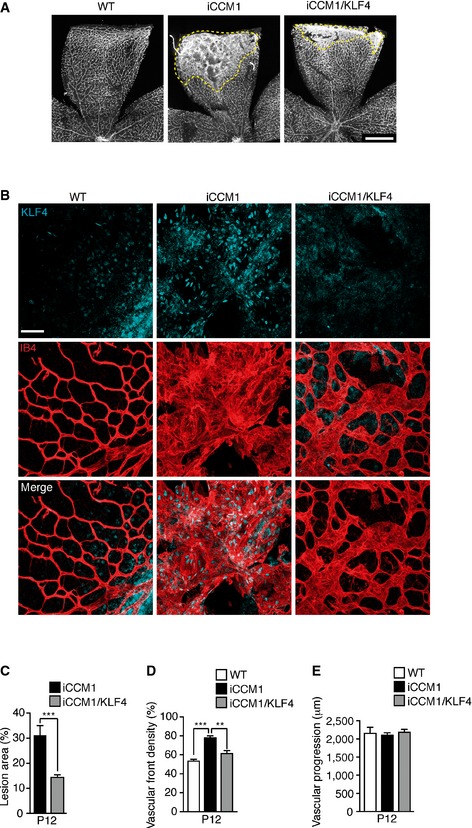
*Klf4* is critical for cavernoma development and progression in the retina AIsolectin B4 staining (IB4, used to identify vasculature) on WT, iCCM1 and iCCM1/KLF4 retinae at P12. Dotted area highlights macroscopic differences in the extension of CCM lesion area between iCCM1 and iCCM1/KLF4 mice. Images are representative of five mice for each genotype. Scale bar: 500 μm.BRepresentative immunostaining (one out of three performed, *n* = 3 in each group) for KLF4 (light blue) in the retinae of WT, iCCM1, and iCCM1/KLF4 mice. Vasculature at the periphery of the retina is shown after isolectin B4 staining (red). Scale bar: 60 μm.C–EQuantification of percentage of retinal area covered by vascular lesions (C), vascular front density at the leading edge of the plexus (D), and average distance covered by the growing vessels measured as vascular progression (E) in retinae from WT, iCCM1, and iCCM1/KLF4 mice at P12. Data are mean ± SD (*n* = 5 for each genotype from three different litters). A two‐tailed unpaired *t*‐test was performed. ***P = 0.0003, **P = 0.004.

**Figure EV2:**
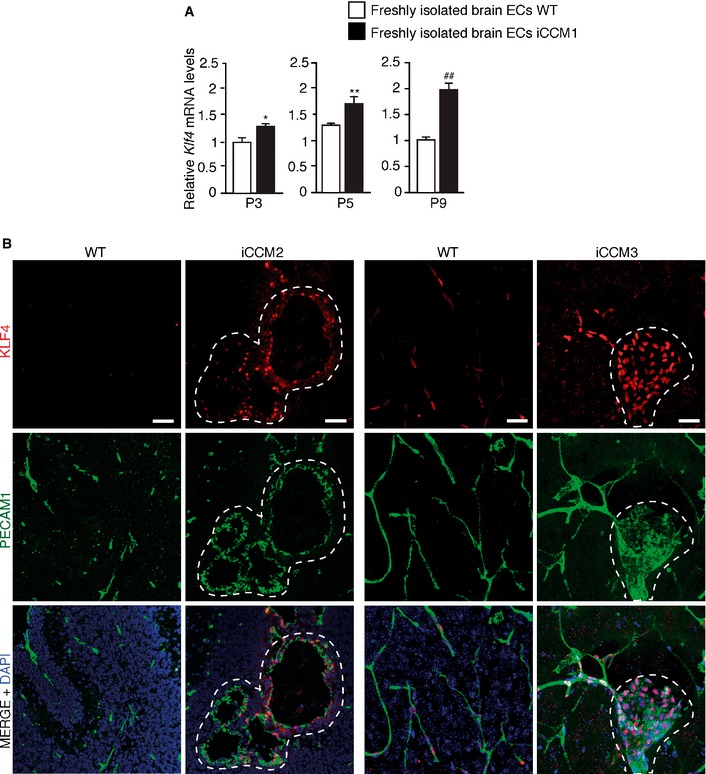
KLF4 amount is increased upon loss of *Ccm1*,* Ccm2,* and *Ccm3* qRT–PCR analysis of *Klf4* during disease progression at different times (P3, P5, and P9) after tamoxifen‐induced *Ccm1* recombination (P1) in freshly isolated brain ECs derived from WT and iCCM1 mice. Data are mean ± SD (*n* = 3 in each group). Fold changes are relative to WT animals. A two‐tailed unpaired *t*‐test was performed. *P = 0.0143, **P = 0.002, ^##^ *P* = 0.001.Representative immunostaining of KLF4 (red) in combination with PECAM1 (green, to identify ECs) in brain sections from iCCM2, iCCM3 mice, and their relative WT controls (one out of three performed). Cell nuclei are visualized with DAPI; dotted area highlights lesion area. Scale bars: 50 μm.

**Figure EV3:**
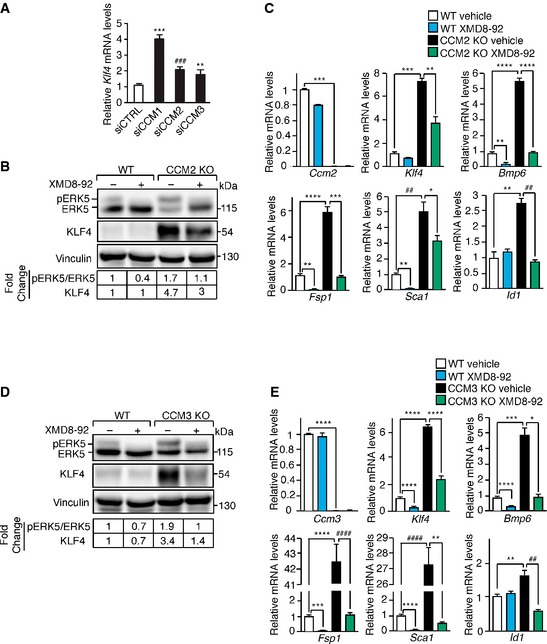
Increased ERK5 phosphorylation is responsible for KLF4 upregulation and KLF4‐dependent EndMT in the absence of both CCM2 and CCM3 qRT–PCR analysis of *Klf4* in WT ECs transfected with either siRNA directed to anyone of the three *Ccm* genes or control siRNA. Data are presented as mean ± SD (*n* = 3). Results are shown as fold changes relative to control siRNA‐treated ECs. A two‐tailed unpaired *t*‐test was performed. ***P = 0.0001, ^###^ *P* = 0.0009, **P = 0.0017.
WB analysis of pERK5, ERK5, and KLF4 in lung‐derived WT and CCM2 KO ECs treated with XMD8‐92 or vehicle for 72 h. Both pERK5/ERK5 ratio and KLF4 amount normalized over vinculin, the loading control, were quantified by densitometry scan. WB results are representative of three independent observations.
qRT–PCR of *Ccm2, Klf4, Bmp6,* and some EndMT markers in WT and CCM2 KO ECs treated with XMD8‐92 or vehicle for 72 h. qRT–PCR results are shown as mean ± SD (*n* = 3), and fold changes are relative to vehicle‐treated WT ECs. A two‐tailed unpaired *t*‐test was performed. *Ccm2*: ***P = 0.0003; *Klf4*: ***P = 0.0002, **P = 0.006; *Bmp6*: **P = 0.0018, ****P = 8.7E‐05; *Fsp1*: **P = 0.0013, ****P = 6.5E‐05, ***P = 0.0008; *Sca1*: **P = 0.001, ^##^ *P* = 0.0025, *P = 0.029; *Id1*: **P = 0.0013, ^##^ *P* = 0.0029.
WB analysis of pERK5, ERK5, and KLF4 in lung‐derived WT and CCM3 KO ECs treated with XMD8‐92 or vehicle for 72 h. Both pERK5/ERK5 ratio and KLF4 were normalized and quantified as in (A).
qRT–PCR of *Ccm3, Klf4, Bmp6,* and some EndMT markers in WT and CCM3 KO ECs treated with XMD8‐92 or vehicle for 72 h quantified as in (C). *Ccm3*: ****P < 0.00001; *Klf4*: ****P < 0.00001; *Bmp6*: ****P < 0.00001, ***P = 0.0009, *P = 0.01; *Fsp1*: ***P = 0.0002, ****P < 0.00001, ^####^ *P* = 3.8E‐05; *Sca1*: ****P < 0.00001, ^####^ *P* = 4.79E‐05, **P = 0.0013; *Id1*: **P = 0.0039, ^##^ *P* = 0.0017.Source data are available online for this figure.

As previously described (Maddaluno *et al*, 2013), tamoxifen‐induced postnatal and endothelial‐specific *Ccm1* deletion (iCCM1) resulted in the development of several vascular lesions of venous origin mostly concentrated in the cerebellum and in the retina that caused 100% mortality between postnatal days 14 (P14) and 15 (P15) (Figs 1A and EV1A). KLF4 nuclear amount was strongly increased in both the brain and retinal vasculature in iCCM1 mice in comparison with matched controls, both in ECs lining the cavernae of any size and in pseudo‐normal vessels (Maddaluno *et al*, 2013) (Figs 1B and C, and EV1B). *Klf4* upregulation was an early event during CCM pathogenesis since it appeared at P3, soon after *Ccm1* gene recombination, in freshly isolated ECs from iCCM1 brains and it remained high during the progression of the disease (Fig EV2A). KLF4 upregulation and pattern of expression were further confirmed in tamoxifen‐inducible endothelial‐specific *Ccm2* (iCCM2) and *Ccm3* (iCCM3) loss‐of‐function mice (Maddaluno *et al*, 2013) (Fig EV2B). Consistently, either silencing any one of the three *Ccm* genes or inducing stable gene deletion in cultured ECs resulted in *Klf4* mRNA and protein upregulation (Fig EV3A–E).

**Figure 2:**
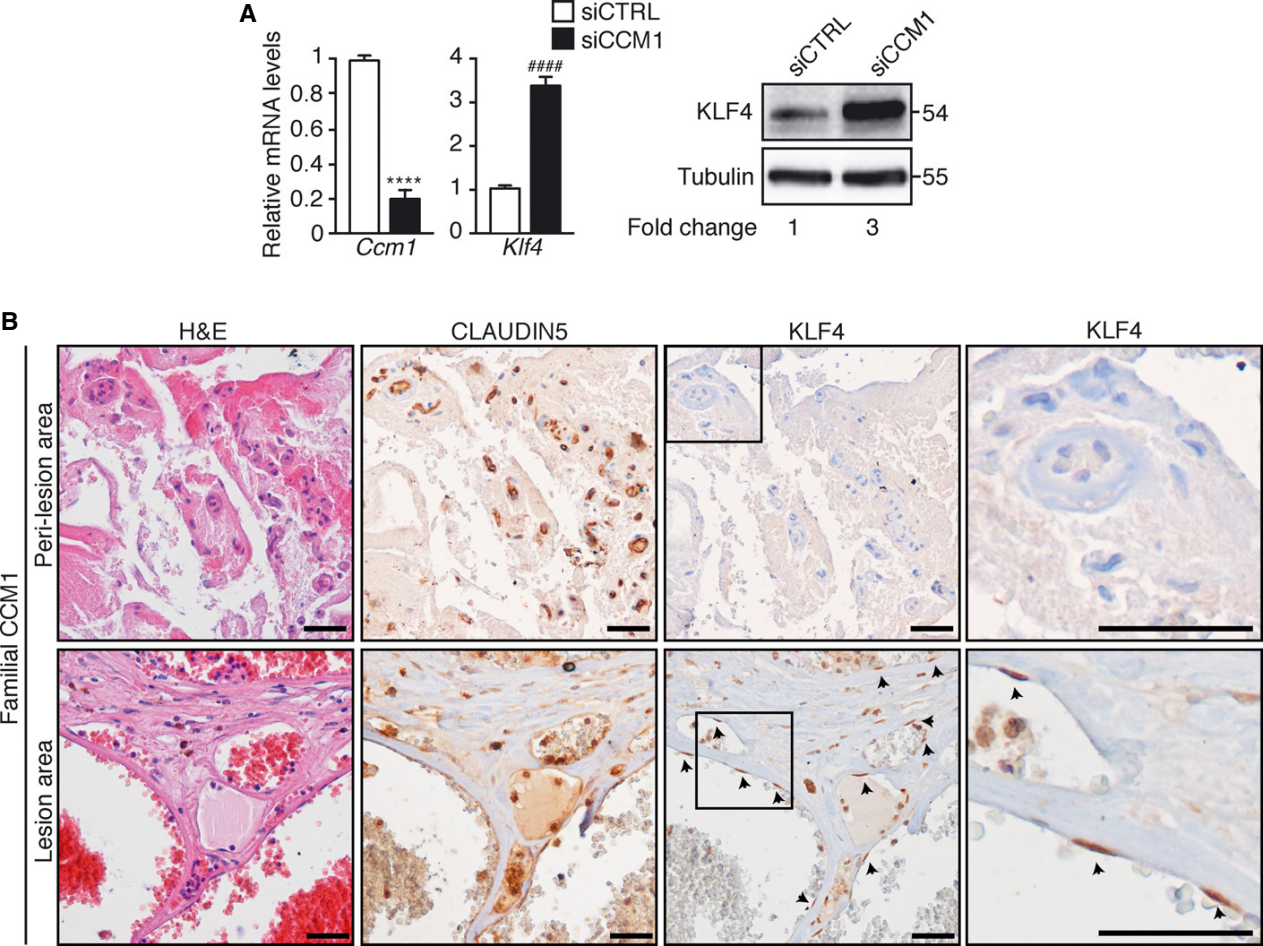
KLF4 is increased in a human brain cell line upon loss of CCM1 and in cerebral lesions of CCM1 patients Left panel: *Ccm1* and *Klf4* relative mRNA levels in *CCM1* (siCCM1) and control (siCTRL) siRNA‐treated hCMEC/D3. The result is shown as fold changes in gene expression in siCCM1‐treated versus control. Data are presented as mean ± SD (*n* = 3). A two‐tailed unpaired *t*‐test was performed. ****P < 0.00001, ^####^ *P* = 1.2E‐05. Right panel: WB analysis of KLF4 amount in siCCM1‐ and siCTRL‐treated hCMEC/D3. Tubulin is used as a loading control. These data are representative of three independent observations.Immunohistochemical analysis performed on serial sections of brain tissue derived from a familial CCM1 patient. Hematoxylin and eosin (left panels), CLAUDIN5 (central panels), and KLF4 (right panels) stainings were performed in brain lesion and normal peri‐lesion vessels as control brain tissue. Higher magnification images of KLF4 staining of the boxed regions are shown. CLAUDIN5 identifies ECs and arrowheads mark endothelial KLF4‐positive nuclei. Scale bar: 100 μm. These data are representative of three independent observations.Source data are available online for this figure.

The increase in KLF4 was observed also in cultured human brain ECs (hCMEC/D3) when *Ccm1* was silenced (Fig 2A) and, most importantly, immunohistochemical analysis of tissue biopsies of CCM1 familial patients confirmed the increase in KLF4 nuclear signal in ECs lining the cavernomas in comparison with normal peri‐lesion vessels (Fig 2B).

We then investigated the role of KLF4 in the development of CCM malformations. To this purpose, we developed endothelial‐specific tamoxifen‐inducible *Ccm1* and *Klf4* double loss‐of‐function mice (iCCM1/KLF4) (Katz *et al*, 2005; Wang *et al*, 2010; Maddaluno *et al*, 2013). The newborn mice were tamoxifen‐injected at P1 to induce the expression of Cre‐recombinase and endothelial‐specific gene ablation. The pups were analyzed within 2 weeks upon gene recombination. *Ccm1* deletion was comparable in freshly isolated brain ECs derived from iCCM1 and iCCM1/KLF4 animals at P12 (Fig 1C). *Klf4* was abrogated in the double KO iCCM1/KLF4 pups as verified by immunofluorescence and qRT–PCR (Fig 1B and C).

iCCM1/KLF4 mice showed a macroscopic reduction in the number, size, and extension of the CCM vascular malformations in the cerebellum (Fig 1A, B, and D). Quantification of the number of cavernomas of any size revealed a 70% reduction in iCCM1/KLF4 in comparison with iCCM1 mice (Fig 1D). Abrogation of *Klf4* also reduced by 75% mouse mortality in *Ccm1*‐deficient pups (Fig 1E).

We previously observed that iCCM1 animals developed vascular malformations also at the periphery of the retinal vascular plexus (Maddaluno *et al*, 2013) (Fig EV1A and B), in line with what was described in 5% of familial CCM patients (Reddy *et al*, 2010). Endothelial‐specific *Klf4* deletion did not alter retinal vasculature formation *per se* (Appendix Fig S1), but significantly reduced the area and the vascular density at the front of malformed retinal vessels *Ccm1* deficient (Fig EV1A–D), while the advancing of the vasculature across the vitreal surface (vascular progression) was not modified (Fig EV1E).

In conclusion, our data indicate that KLF4 is required for the development and progression of CCM1 vascular malformations.

### KLF4 induces EndMT in CCM1‐null ECs

**Figure 3:**
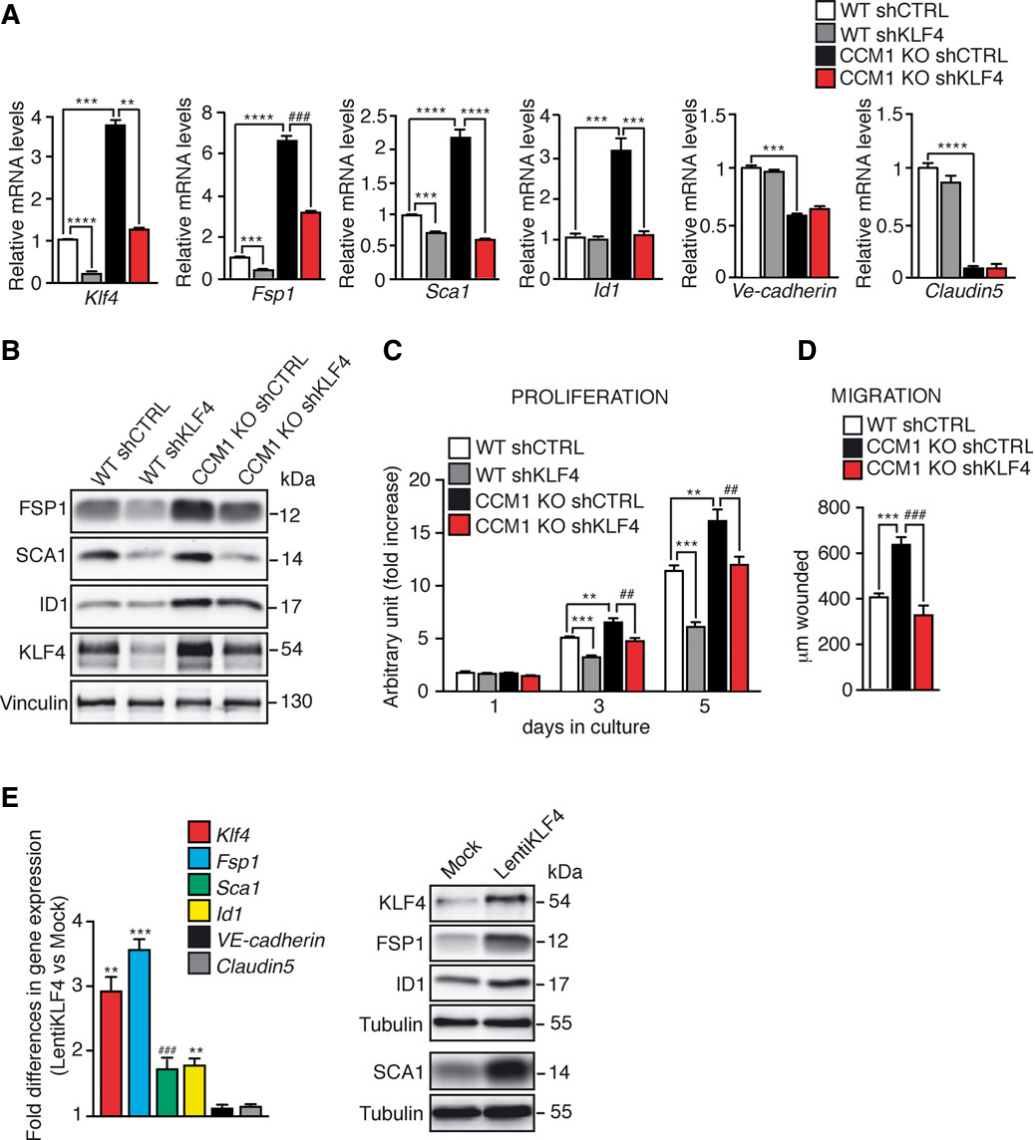
KLF4 regulates the EndMT switch in CCM1 KO ECs A–DCultured lung‐derived WT and CCM1 KO ECs were lentivirally transduced with shRNA directed to either *Klf4* (shKLF4) or control sequence (shCTRL). (A) qRT–PCR of mesenchymal (*Fsp1, Id1*), stem cell‐like (*Sca1*), and endothelial markers (*VE‐cadherin* and *Claudin5*) in WT shCTRL, WT shKLF4, CCM1 KO shCTRL, and CCM1 KO shKLF4 ECs. Data are mean ± SD (*n* = 3). Fold difference in gene expression is relative to WT shCTRL ECs. A two‐tailed unpaired *t*‐test was performed. *Klf4*: ****P < 0.00001, ***P = 0.0008, **P = 0.007; *Fsp1*: ***P = 0.0001, ****P = 3.9E‐05, ^###^ *P* = 0.0002; *Sca1*: ***P = 0.0001, ****P < 0.00001; *Id1*: ***P = 0.0001; *Ve‐cadherin*: ***P = 0.0001; *Claudin5*: ****P = 1.37E‐05. (B) WB of EndMT markers in WT shCTRL, WT shKLF4, CCM1 KO shCTRL, and CCM1 KO shKLF4 ECs. Vinculin is the loading control. These data are representative of three independent observations. (C) Proliferation rate of WT shCTRL, WTshKLF4, CCM1 KO shCTRL, and CCM1 KO shKLF4 ECs cultured for 5 days. Columns represent mean ± SD (*n* = 8). A two‐tailed unpaired *t*‐test was performed. 3 days of culture: ***P = 0.0001, **P = 0.0045, ^##^ *P* = 0.0017; 5 days of culture: ***P = 0.0001, **P = 0.0029, ^##^ *P* = 0.0040. (D) Migration rate measured in a wound assay of WT shCTRL, CCM1 KO shCTRL, and CCM1 KO shKLF4 ECs. Mean ± SD is shown (*n* = 6). A two‐tailed unpaired *t*‐test was performed. ***P = 0.0004, ^###^ *P* = 0.0009.ECultured lung‐derived WT ECs were lentivirally transduced with a full‐length murine *Klf4* (LentiKLF4) or empty vector (Mock). qRT–PCR (left panel) and WB (right panel) of EndMT markers in Mock and LentiKLF4 ECs. qRT–PCR data are mean ± SD (*n* = 3) and the fold changes are relative to Mock ECs. A two‐tailed unpaired *t*‐test was performed. **P = 0.002, ***P = 0.0007, ^###^ *P* = 0.0001. WB results are representative of three independent observations. Tubulin is the loading control.Source data are available online for this figure.

**Figure EV4:**
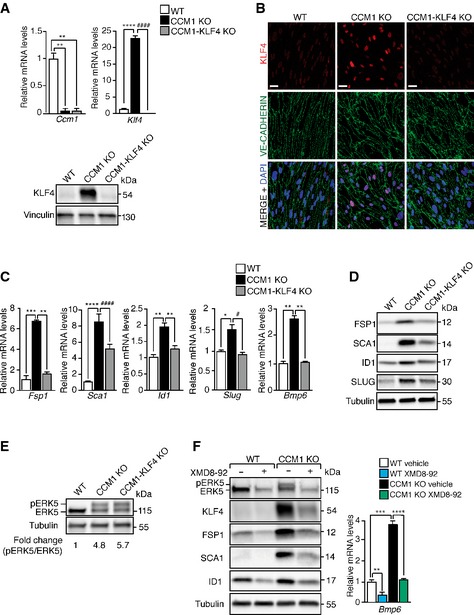
ERK5‐KLF4 axis regulates EndMT in primary brain CCM1 KO ECs Upper panel: qRT–PCR of both *Ccm1* and *Klf4* gene recombination efficiency after *in vitro* TAT‐Cre‐recombinase treatment of primary brain ECs derived from WT,* Ccm1* ^fl/fl^, and *Ccm1* ^fl/fl^/*Klf4* ^fl/fl^ mice to originate cultured WT, CCM1 KO, and CCM1‐KLF4 KO brain ECs, respectively (10 mice for each genotype per experimental replicate). Data are presented as mean ± SD (*n* = 3). Fold changes are relative to WT brain ECs. A two‐tailed unpaired *t*‐test was performed. *Ccm1*: **P = 0.001; *Klf4*: ****P < 0.00001, ^####^ *P* = 5.2E‐05. Lower panel: WB of KLF4 in WT, CCM1 KO, and CCM1‐KLF4 KO brain ECs described above. Vinculin is the loading control. These data are representative of three independent observations.Representative confocal analysis (out of three performed) of KLF4 (red) and VE‐CADHERIN (green) in WT, CCM1 KO, and CCM1‐KLF4 KO primary brain ECs obtained as in (A). Scale bar: 30 μm.
qRT–PCR of EndMT markers in WT, CCM1 KO, and CCM1‐KLF4 KO brain ECs obtained as in (A). Data are mean ± SD (*n* = 3). Fold changes are relative to WT ECs. A two‐tailed unpaired *t*‐test was performed. *Fsp1*: ***P = 0.0006, **P = 0.0011; *Sca1*: ****P < 0.00001, ^####^ *P* = 7.7E‐05; *Id1*: **P = 0.0017; *Slug*: *P = 0.02, ^#^ *P* = 0.01; *Bmp6*: **P = 0.003.
WB of FSP1, SCA1, ID1, and SLUG in WT, CCM1 KO, and CCM1‐KLF4 KO brain ECs obtained as described in (A). Tubulin is the loading control. These data are representative of three independent observations.Analysis of pERK5 and ERK5 protein levels in WT, CCM1 KO, and CCM1‐KLF4 KO brain ECs obtained as in (A). pERK5/ERK5 ratio normalized over tubulin is indicated.
WB analysis of pERK5, ERK5, KLF4, FSP1, SCA1, ID1 (left panel), and qRT–PCR of *Bmp6* (right panel) in WT and CCM1 KO brain ECs, obtained as in (A), and then treated with XMD8‐92 or vehicle for 72 h. qRT–PCR results are shown as mean ± SD (*n* = 3) and fold changes are relative to vehicle‐treated WT ECs. A two‐tailed unpaired *t*‐test was performed. **P = 0.001, ***P = 0.0006, ****P = 6.3E‐05. WB results are representative of three independent observations, and tubulin is the loading control.Source data are available online for this figure.

As we previously reported and extensively characterized both *in vivo* and *in vitro,* endothelial‐specific disruption of *Ccm* genes induces the EndMT switch in ECs lining the cavernomas (Maddaluno *et al*, 2013). Thus, the simultaneous expression of endothelial‐specific molecules (PECAM1, VE‐CADHERIN, or isolectin B4) together with typical EndMT markers was detected in CCM1 KO ECs (Maddaluno *et al*, 2013 and Appendix Fig S2). To investigate whether KLF4 had a key role in this process, we performed *Klf4* silencing in CCM1 WT and KO ECs by lentiviral vector‐mediated trasduction of shRNA. *Klf4* knockdown strongly reduced mRNA and protein amount of markers of the stem cell/EndMT phenotype (Fig 3A and B) that were upregulated in cultured CCM1 KO ECs. Comparable data were obtained when *Ccm1* and *Klf4* LoxP‐site flanked genes were concomitantly deleted by TAT‐Cre‐recombinase (Fig EV4A–D and F).

In these experimental conditions, abrogation of *Klf4* expression did not prevent the decrease and disorganization of *VE‐Cadherin* and *Claudin5*, previously reported in CCM1‐ablated cells (Figs 3A and EV4B).

In the absence of *Ccm1*, cell transition to a mesenchymal phenotype is accompanied by increased proliferation and migration (Wustehube *et al*, 2010; Maddaluno *et al*, 2013). Both parameters were impaired by *Klf4* silencing in cultured CCM1 KO ECs (Fig 3C and D).

To investigate whether KLF4 is sufficient to promote EndMT, we infected WT ECs in culture with a lentiviral vector expressing *Klf4* (LentiKLF4) or an empty vector (Mock) as control, achieving a threefold *Klf4* induction (Fig 3E), a level of upregulation comparable to that observed in CCM1 KO ECs. KLF4 upregulation in WT ECs increased EndMT markers at both mRNA (Fig 3E, left panel) and protein levels (Fig 3E, right panel), whereas it did not affect the expression of the endothelial junction molecules as *VE‐cadherin* and *Claudin 5* (Fig 3E, left panel).

**Figure EV5:**
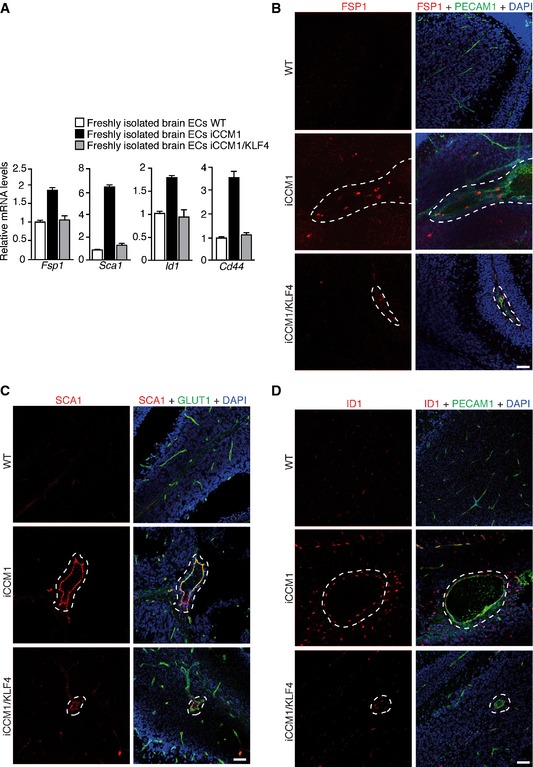
EndMT marker expression is reduced in the absence of KLF4 *ex vivo* and *in vivo* A
qRT–PCR of some EndMT markers in freshly isolated brain ECs from WT, iCCM1, and iCCM1/KLF4 mice analyzed at P12. Fold changes are relative to WT animals. Data are mean ± SD from a representative experiment out of three.B–DRepresentative confocal analysis of (B) PECAM1 (green) and FSP1 (red), (C) GLUT1 (green) and SCA1 (red), or (D) PECAM1 (green) and ID1 (red) in normal cerebellar vessels of WT mice and vascular lesions (dotted area) of both iCCM1 and iCCM1/KLF4 mice (*n* = 4 in each group). PECAM1 identifies ECs; DAPI visualizes nuclei. Scale bars: 50 μm.

Most importantly, in the absence of KLF4, EndMT switch was strongly prevented both in freshly isolated brain ECs (Fig EV5A) and in lesions of iCCM1/KLF4 mice (Fig EV5B–D).

The role of KLF4 as inducer of EndMT appears cell‐context‐dependent, since in the absence of CCM1 KLF4 was increased in ECs of different organs but the full EndMT marker upregulation was detected only in brain ECs (Appendix Fig S3).

In the retina vasculature, KLF4 was higher in veins than in arteries in both WT and iCCM1 mice and further increased in the lesion (Appendix Fig S4A–C). Interestingly, ID1 expression in the retina seemed to correlate with KLF4 (Appendix Fig S4A, B and D). These data suggest that higher levels of KLF4 in veins may explain the venous origin of the cavernomas.

Taken together, these results show that KLF4 upregulation is critical to promote the EndMT switch induced by CCM1 abrogation. However, the strength of this effect is context‐dependent being observed specifically in the brain vasculature.

### KLF4 regulates BMP6‐mediated signaling

**Figure 4:**
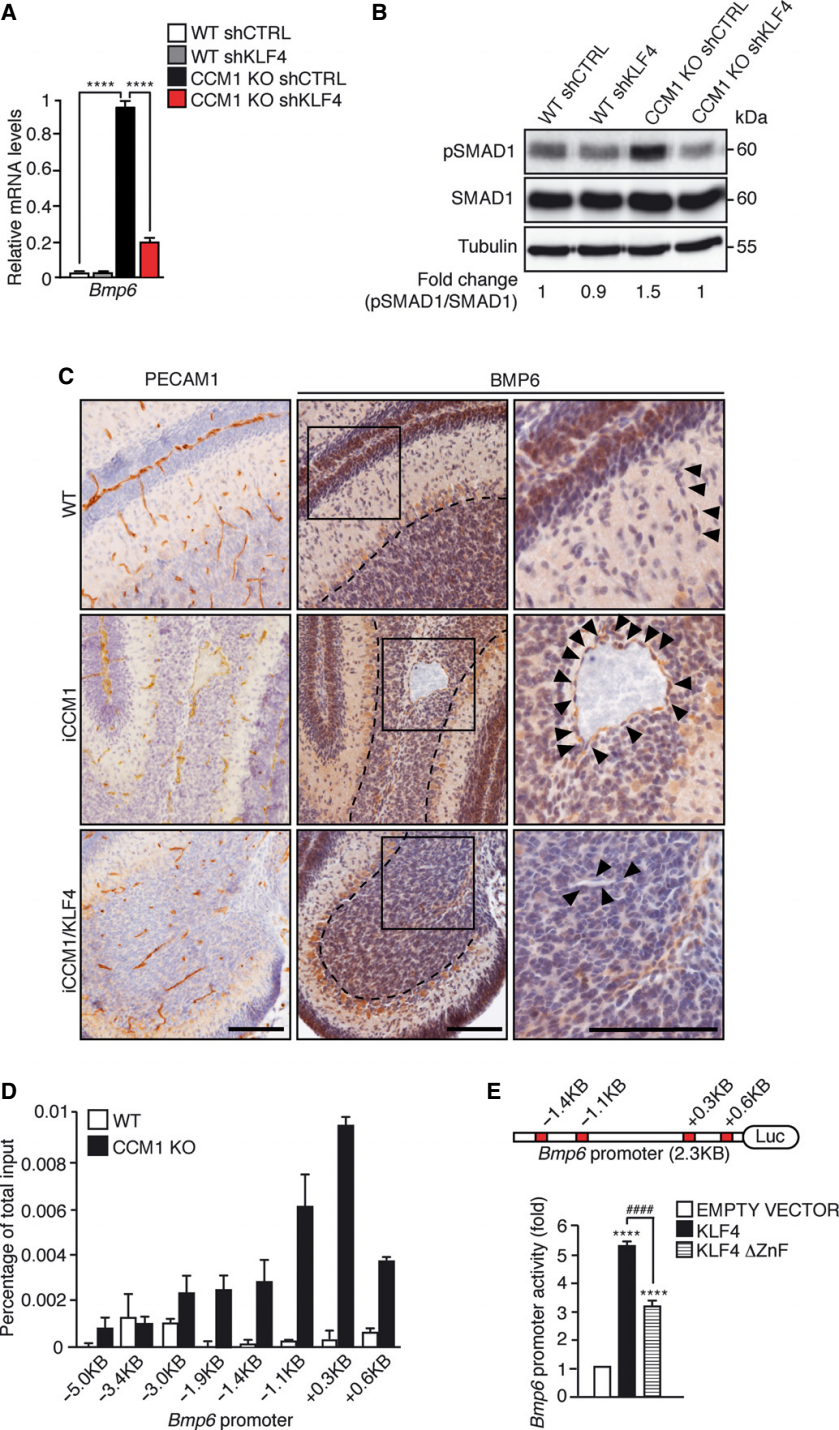
KLF4 increases *Bmp6* expression and SMAD1 phosphorylation in ECs qRT–PCR of *Bmp6* expression in WT shCTRL, WT shKLF4, CCM1 KO shCTRL, and CCM1 KO shKLF4‐cultured ECs. Data represent the mean ± SD (*n* = 3). Fold changes are relative to CCM1 KO shCTRL ECs. A two‐tailed unpaired *t*‐test was performed. ****P < 0.00001.
WB of pSMAD1 and SMAD1 in WT shCTRL, WT shKLF4, CCM1 KO shCTRL, and CCM1 KO shKLF4 ECs. pSMAD1/SMAD1 ratio normalized over tubulin was quantified by densitometry scan. These data are representative of three independent observations.Immunohistochemical analysis of PECAM1 (left panels) and BMP6 (central panels) performed on serial sections of cerebellum derived from WT, iCCM1, and iCCM1/KLF4 mice at P12. Higher magnification images of the boxed regions are shown in the right panels. Black arrowheads mark ECs and dotted area indicates the Purkinje cell layer used as a positive control of the staining. Scale bar: 100 μm. These data are representative of three independent observations (*n* = 3 for each genotype).ChIP analysis of KLF4 binding to *Bmp6* promoter. Putative KLF4 binding sites identified by MatInspector are indicated. The levels of DNA are normalized to input. Columns are mean ± SD of triplicates from a representative experiment out of three.Transcriptional reporter assay performed in HEK‐293 cells transfected with *Bmp6* promoter reporter plasmid together with an empty vector, a full‐length KLF4 or a mutant KLF4 lacking the DNA‐binding zinc finger domains (KLF4 ∆ZnF). Red boxes in the picture indicate KLF4 binding sites validated by ChIP. Fold change in the *Bmp6* promoter activity is relative to empty vector‐transfected cells. Data are mean ± SD (*n* = 3). A two‐tailed unpaired *t*‐test was performed. ****P < 0.00001, ^####^ *P* = 5.8E‐05.Source data are available online for this figure.

Since BMP6 upregulation triggers the transcription program leading to EndMT in *Ccm1‐*deficient cells and mice (Maddaluno *et al*, 2013), we asked whether it could be regulated by KLF4. We observed that *Klf4* silencing strongly decreased *Bmp6* expression and SMAD1 phosphorylation (pSMAD1) in cultured CCM1 KO ECs (Fig 4A and B). Immunohistochemical analysis of brain tissues confirmed BMP6 reduction in ECs lining the cavernomas of iCCM1/KLF4 mice compared to iCCM1 animals (Fig 4C).

Since eight putative KLF4 binding sites were identified within the murine *Bmp6* promoter region at −5.0 kb and +1.0 kb from the transcription start site (tss), we hypothesized *Bmp6* as a direct transcriptional target of KLF4. In ChIP assay, KLF4 binding was enriched at the selected sites on *Bmp6* promoter in CCM1 KO compared to WT ECs, suggesting that *Bmp6* is a KLF4 target gene (Fig 4D). To demonstrate that the binding of KLF4 to *Bmp6* promoter is functionally active, a transcriptional reporter assay was performed by co‐transfecting the *Bmp6* reporter construct (generated by cloning the promoter region enriched in KLF4 binding sites upstream of the luciferase cDNA) together with an expression plasmid of a full‐length KLF4 or a mutant KLF4 lacking the DNA‐binding zinc finger domains (KLF4 ∆ZnF). We found that KLF4 induced *Bmp6* promoter activity and this effect was reduced when KLF4 binding to DNA was altered (Fig 4E).

**Figure 5:**
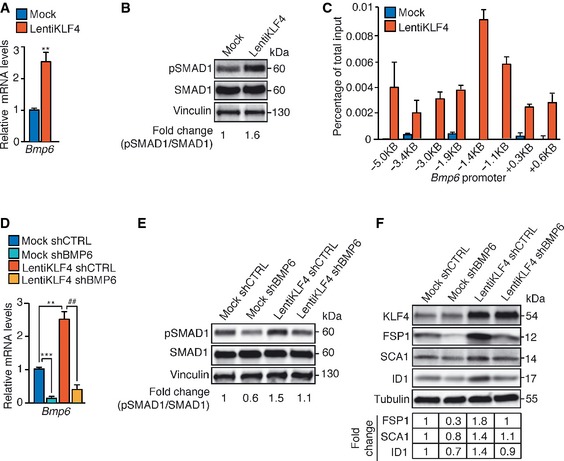
KLF4 promotes EndMT through BMP6 upregulation A
qRT–PCR of *Bmp6* in Mock and LentiKLF4‐cultured ECs. Data are mean ± SD (*n* = 3). Fold changes are relative to Mock ECs. A two‐tailed unpaired *t*‐test was performed. **P = 0.0034.B
WB of pSMAD1 and SMAD1 in Mock and LentiKLF4 ECs. Vinculin is the loading control. pSMAD1/SMAD1 ratio normalized over vinculin was quantified by densitometry scan. These data are representative of three independent observations.CChIP of KLF4 interaction with *Bmp6* promoter in Mock and LentiKLF4 ECs. Putative KLF4 binding sites identified by MatInspector are indicated. The levels of DNA are normalized to input. Columns are mean ± SD of triplicates from a representative experiment out of three.D–FMock and LentiKLF4‐cultured ECs were lentivi rally transduced with shRNA directed to either *Bmp6* (shBMP6) or control sequence (shCTRL). (D) qRT–PCR analysis of *Bmp6* in Mock shCTRL, Mock shBMP6, LentiKLF4 shCTRL, and LentiKLF4 shBMP6 ECs. qRT–PCR data represent the mean ± SD (*n* = 3) and the fold changes are relative to Mock shCTRL ECs. A two‐tailed unpaired *t*‐test was performed. ***P = 0.0008, **P = 0.004, ^##^ *P* = 0.003. WB of pSMAD1 and SMAD1 (E) or EndMT markers (F) in Mock shCTRL, Mock shBMP6, LentiKLF4 shCTRL, and LentiKLF4 shBMP6‐cultured ECs. pSMAD1/SMAD1 ratio was quantified as in (B). These WB data are representative of three independent observations. Tubulin is the loading control. The fold changes of FSP1, SCA1, and ID1 normalized over tubulin were quantified by densitometry scan. These WB data are representative of three independent observations.Source data are available online for this figure.

Furthermore, both *Bmp6* mRNA (Fig 5A) and pSMAD1 (Fig 5B) were upregulated in KLF4‐overexpressing cells and, by ChIP analysis, KLF4 was associated with the selected sites within the *Bmp6* promoter region (Fig 5C). Stable *Bmp6* silencing by lentivirally transduction of shRNA (Fig 5D) prevented the increase of both pSMAD1 (Fig 5E) and EndMT markers induced by KLF4 in LentiKLF4 ECs (Fig 5F).

Overall, our results demonstrate that KLF4 increases *Bmp6* expression that, in turn, contributes to the EndMT program of CCM1‐null ECs.

### KLF4 directly regulates the expression of some EndMT markers

**Figure 6:**
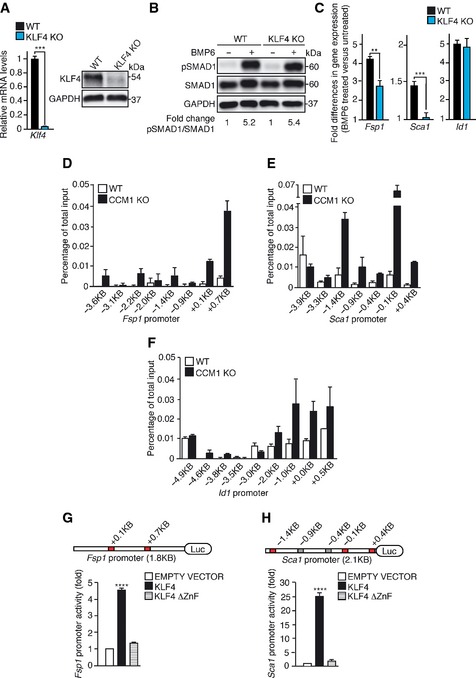
KLF4 directly regulates some EndMT marker expression A
qRT–PCR (left panel) and WB (right panel) analyses of KLF4 in cultured lung WT and KLF4 KO ECs. qRT–PCR data represent the mean ± SD (*n* = 3) and the fold changes are relative to WT ECs. A two‐tailed unpaired *t*‐test was performed. ***P = 0.0006. GAPDH is the loading control in WB. These WB data are representative of three independent observations.B
WB of pSMAD1 and SMAD1 in WT and KLF4 KO ECs left untreated or treated with recombinant BMP6 for 4 h. pSMAD1/SMAD1 ratio normalized over GAPDH was quantified by densitometry scan. These WB data are representative of three independent observations.C
qRT–PCR of *Fsp1*,* Sca1,* and *Id1* in WT and KLF4 KO ECs stimulated with BMP6 for 96 h. qRT–PCR data are mean ± SD (*n* = 3). Fold changes in gene expression in BMP6‐treated versus untreated ECs. A two‐tailed unpaired *t*‐test was performed. **P = 0.0032, ***P = 0.0007.D–FChIP analysis of KLF4 binding to the promoters of *Fsp1* (D), *Sca1* (E), and *Id1* (F) in WT and CCM1 KO cultured ECs. The positions of the putative KLF4 binding sites identified with MatInspector in the promoters of the genes analyzed are indicated. The levels of DNA are normalized to input. Columns are mean ± SD of triplicates from a representative experiment out of three performed.G, HTranscriptional reporter assays performed in HEK‐293 cells transfected with either *Fsp1* (G) or *Sca1* (H) promoter reporter plasmid together with an empty vector, KLF4 or KLF4 ∆ZnF. In the schematics, red boxes indicate KLF4 binding sites validated by ChIP, while grey boxes indicate KLF4 binding sites not validated by ChIP. Fold change in the promoter activity is relative to empty vector‐transfected cells. Data are mean ± SD (*n* = 3). A two‐tailed unpaired *t*‐test was performed. ****P < 0.00001.Source data are available online for this figure.

We then investigated whether KLF4‐induced EndMT is fully dependent on the upregulation of BMP6 or whether KLF4 could also have an independent and direct effect on EndMT marker expression. To this purpose, we treated WT and KLF4 KO ECs (Fig 6A) with recombinant BMP6 and we analyzed a set of representative mesenchymal and stem cell markers. Although the induction of SMAD1 phosphorylation was comparable (Fig 6B), BMP6‐induced expression of *Fsp1* and *Sca1*, but not *Id1*, was reduced in the absence of KLF4 (Fig 6C), suggesting a direct contribution of KLF4 to the upregulation of some EndMT markers. Several consensus sequences for KLF4 were identified in the promoter region of murine *Fsp1*,* Sca1,* and *Id1*. ChIP analysis revealed KLF4 binding to both *Fsp1* (Fig 6D) and *Sca1* promoters (Fig 6E), with the binding occurring mainly in the regions located at +0.1 kb and +0.7 kb (*Fsp1*) and −1.4 kb and +0.4 kb (*Sca1*) from the tss, respectively. Conversely, KLF4 binding to *Id1* promoter was barely detectable, suggesting that Id1, a *bona fide* BMP6 target gene (Korchynskyi & ten Dijke, 2002), is not directly regulated by KLF4 (Fig 6F). Moreover, both *Fsp1* and *Sca1* transcriptional reporters (generated by cloning the promoter regions enriched in KLF4 binding sites identified by ChIP upstream of the luciferase cDNA) were strongly induced by KLF4 in a DNA‐binding‐dependent manner (Fig 6G and H).

Thus besides inducing BMP6, KLF4 can also directly upregulate some EndMT markers.

### Erk5 activation mediates KLF4 upregulation and KLF4‐dependent EndMT in the absence of *Ccm1*

We then approached the problem of the molecular mechanism through which loss of *Ccm* results in increased *Klf4* expression in the brain and retinal vasculature. ERK5 phosphorylation and activation have been reported to promote *Klf2* and *Klf4* transcription in ECs (Dekker *et al*, 2002; Ohnesorge *et al*, 2010; Komaravolu *et al*, 2015). Myocyte enhanced (MEF) family of transcription factors, downstream targets of ERK5, are directly involved in pitavastatin‐dependent KLF4 upregulation in HUVEC (Maejima *et al*, 2014).

**Figure 7:**
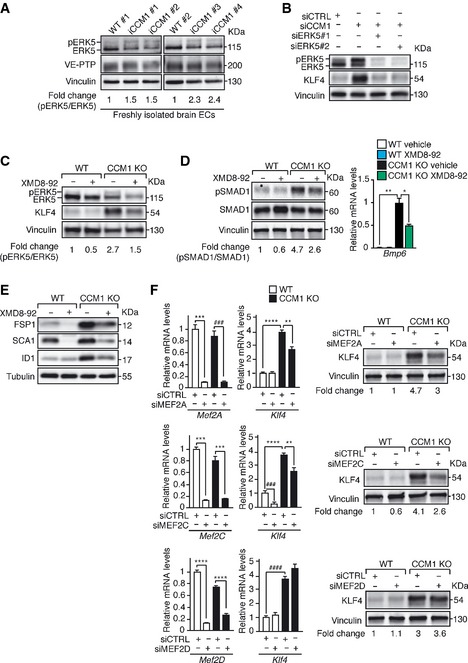
In the absence of CCM1 increased ERK5 phosphorylation is responsible for KLF4 upregulation and KLF4‐dependent EndMT A
WB of phoshorylated ERK5 (pERK5) and total ERK5 in freshly isolated brain ECs from WT (*n* = 2) and iCCM1 (*n* = 4) mice from two different litters at P12. VE‐PTP measures the endothelial content and vinculin is the loading control. pERK5/ERK5 ratio normalized over vinculin and VE‐PTP was quantified by densitometry scan.B
WB of pERK5, ERK5, and KLF4 in cultured lung‐derived WT ECs either *Ccm1* (siCCM1) or control (siCTRL) siRNA‐treated alone or in combination with two siRNA targeting *Erk5* (siERK5#1,2). pERK5/ERK5 ratio normalized over vinculin is indicated. These data are representative of three independent observations.C–ETreatment of cultured lung‐derived WT and CCM1 KO ECs with either 5 μM of XMD8‐92 or the vehicle (72 h). (C) WB analysis of pERK5, ERK5, and KLF4. pERK5/ERK5 ratio was quantified as in (B). (D) Left panel: evaluation of pSMAD1 and SMAD1 protein levels. pSMAD1/SMAD1 ratio normalized over vinculin is shown. These data are representative of three independent observations. Right panel: qRT–PCR of *Bmp6*. The data represent the mean ± SD (*n* = 3). Fold changes are relative to vehicle‐treated CCM1 KO ECs. A two‐tailed unpaired *t*‐test was performed. **P = 0.001, *P = 0.01. (E) Representative WB of EndMT markers (one out of three performed). Tubulin is the loading control.F
qRT–PCR analysis of *Klf4*,* Mef2A*,* Mef2C,* and *Mef2D* (left panels) and WB of KLF4 (right panels) in cultured WT and CCM1 KO ECs treated with either control siRNA (siCTRL) or siRNA directed to any of *Mef2A* (upper panels), *Mef2C* (central panels), or *Mef2D* (lower panels). qRT–PCR data represent the mean ± SD (*n* = 3) and the fold changes are relative to WT siCTRL ECs. A two‐tailed unpaired *t*‐test was performed. Upper panels: ***P = 0.0002, ^###^ *P* = 0.0005, ****P = 4.09E‐05, **P = 0.001; central panels: ***P = 0.0005 (WT siCRTL vs WT siMEF2C), ***P = 0.0006 (KO siCRTL vs KO siMEF2C), ^###^ *P* = 0.0001, ****P < 0.00001, **P = 0.007; lower panels: ****P = 5.6E‐05 (WT siCRTL vs WT siMEF2D), ****P = 1.53E‐05 (KO siCRTL vs KO siMEF2D), ^####^ *P* = 3.1E‐05. For WB, vinculin is used as a loading control and data are representative of three independent observations.Source data are available online for this figure.

We found that ERK5 phosphorylation was increased in freshly isolated brain‐derived ECs of iCCM1 mice in comparison with matched controls (Fig 7A). Accordingly, ERK5 phosphorylation was higher in cultured ECs upon *Ccm1* ablation (Figs 7B and C, and EV4E and F).

To investigate whether ERK5 activation was responsible for the upregulation of both KLF4 and EndMT marker expression in the absence of CCM1, we transfected WT ECs with siRNAs directed to *Erk5* and *Ccm1*. The downregulation of ERK5 strongly prevented KLF4 upregulation in *Ccm1*‐silenced ECs (Fig 7B). Similarly, the treatment of cultured CCM1 KO ECs with XMD8‐92, a specific ERK5 inhibitor (Yang *et al*, 2010; Komaravolu *et al*, 2015), strongly inhibited KLF4 overexpression, *Bmp6* upregulation, SMAD1 phosphorylation, and EndMT marker acquisition (Figs 7C–E and EV4F). Activated ERK5 promoted KLF4 upregulation through MEF2A and MEF2C transcription factors in CCM1 KO ECs, since both *Mef2A* and *Mef2C* knockdown in CCM1 KO ECs reduced significantly KLF4 (Fig 7F top and middle panels, respectively). Conversely, *Mef2D* silencing did not modify KLF4 amount (Fig 7F bottom panel).

To establish ERK5‐mediated KLF4 upregulation and KLF4‐dependent EndMT as common molecular nodes in CCM, we analyzed ERK5 activation, KLF4 amount, and EndMT marker expression in WT, CCM2 KO, or CCM3 KO ECs treated with XMD8‐92 or vehicle as control. We found that in ECs null for either *Ccm2* or *Ccm3* ERK5 phosphorylation was increased (Fig EV3B and D, respectively). Furthermore, the treatment of cultured CCM2 KO and CCM3 KO ECs with XMD8‐92 strongly inhibited KLF4 overexpression, *Bmp6* upregulation and EndMT marker acquisition (Fig EV3B–E).

**Figure 8:**
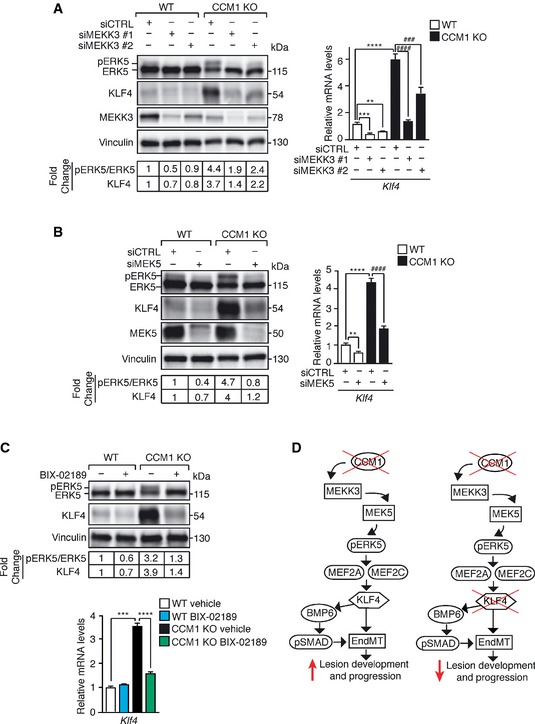
MEKK3‐MEK5‐dependent ERK5 phosphorylation is responsible for KLF4 upregulation Left panel: WB analysis of pERK5, ERK5, KLF4, and MEKK3 in WT and CCM1 KO ECs treated with either two different siRNAs against *Mekk3* (siMEKK3 #1 and siMEKK3 #2) or a control sequence (siCTRL). Both pERK5/ERK5 ratio and KLF4 amount normalized over vinculin were quantified by densitometry scan. WB results are representative of three independent observations. Right panel: qRT–PCR of *Klf4* in WT and CCM1 KO ECs treated with two different siRNAs directed to *Mekk3* (siMEKK3 #1 and siMEKK3 #2) or a control sequence (siCTRL). qRT–PCR results are shown as mean ± SD (*n* = 3), and fold changes are relative to siCTRL‐treated WT ECs. A two‐tailed unpaired *t*‐test was performed. ***P = 0.0002, **P = 0.002, ****P = 2.5E‐05, ^####^ *P* = 3.1E‐05, ^###^ *P* = 0.0007.
WB analysis of pERK5, ERK5, KLF4, and MEK5 (left panel) and qRT–PCR of *Klf4* (right panel) in WT and CCM1 KO ECs treated with a siRNA against MEK5 or a control sequence (siCTRL) quantified as in (A). **P = 0.0035, ****P < 0.00001, ^####^ *P* = 3.5E‐05.
WB analysis of pERK5, ERK5, and KLF4 (upper panel) and qRT–PCR of *Klf4* (lower panel) in WT and CCM1 KO ECs treated with BIX‐02189 or vehicle for 48 h quantified as in (A). Fold changes are relative to vehicle‐treated WT ECs. A two‐tailed unpaired *t*‐test was performed. ***P = 0.0002, ****P = 2.1E‐05.Schematic model of KLF4 activity and regulation during CCM pathogenesis.Source data are available online for this figure.

MAPK/ERK kinase kinase 3 (MEKK3), previously identified as a CCM2 and CCM1 binding partner in the osmosensing complex (Uhlik *et al*, 2003; Zhou *et al*, 2015), activates MAPK/ERK kinase 5 (MEK5) that, in turn, phoshorylates ERK5 (Kato *et al*, 1997; Sohn *et al*, 2005). We therefore explored the possibility that the lack of CCM might increase *Klf4* expression through the MEKK3‐MEK5‐ERK5 signaling axis. Both *Mekk3* (Fig 8A) and *Mek5* (Fig 8B) silencing, as well as the treatment of cultured CCM1 WT and KO ECs with BIX‐02189 (Fig 8C), a specific MEK5 inhibitor (Tatake *et al*, 2008), strongly reduced ERK5 phosphorylation and KLF4 amount in the absence of CCM1.

Thus, *Ccm* gene ablation results in increased MEKK3‐MEK5‐dependent ERK5 phosphorylation responsible for KLF4 upregulation and KLF4‐dependent EndMT.

## Discussion

We report here that the transcription factor KLF4 is a crucial determinant of the development of CCM. In human vascular lesions and in mice deficient of anyone of the three *Ccm* genes, KLF4 is strongly upregulated in ECs lining the cavernomas. Most importantly, *Klf4* genetic inactivation blocks the development of the vascular lesions and mortality. To our knowledge, this is the first targeting condition that essentially abolishes the 100% mortality observed in iCCM1 mice due to brain hemorrhage.

The mechanism of action of KLF4 in inducing CCM malformations is complex and the data reported here define some of the molecular steps involved (Fig 8D). As previously shown (Maddaluno *et al*, 2013), the formation of cavernomas is linked to the transition of ECs lining the malformations to a mesenchymal phenotype (EndMT switch). In the absence of CCM1, EndMT is mediated by the increase of endogenous *Bmp6* that we show here to be induced by KLF4. *Klf4* expression increases soon after *Ccm* ablation and remains high in ECs lining the CCM lesions. KLF4 induction could therefore contribute to the induction and progression of the disease by upregulating BMP6.

KLF4 can also induce EndMT by binding directly to the promoters of some EndMT markers (*Sca1* and *Fsp1*). Intriguingly, the promoter regions of the EndMT markers that showed a higher binding affinity for KLF4 are surrounded by putative SMAD‐binding sites. Since pSMADs bind weakly to target gene promoters (Shi *et al*, 1998), it is possible that their DNA binding is reinforced through the association with KLF4, adding a further level of regulation of SMAD activity by KLF4 (Li *et al*, 2010).

KLF4 has pleiotropic functions in EC biology. It is widely considered a vascular protective factor due to its ability to activate specific transcriptional programs with anti‐inflammatory, anticoagulant, and antioxidant roles in the endothelium (Hamik *et al*, 2007; Zhou *et al*, 2012; Shatat *et al*, 2014). However, recent publications have associated KLF4 also with endothelial diseases, since it can promote sprouting angiogenesis by regulating Notch pathway and it is upregulated in several pathological conditions (Chen *et al*, 2014; Hale *et al*, 2014; Magrini *et al*, 2014). Although KLF4 may act either as an inducer or a suppressor of cell proliferation in different cell types (McConnell & Yang, 2010), several publications reported KLF4 as a promoter of endothelial cell growth (Hale *et al*, 2014; Wang *et al*, 2015). These data are consistent with the fact that high KLF4 amount in CCM1 KO ECs results in increased cell growth and *Klf4* silencing lowers cell proliferation in both WT and CCM1‐null ECs.

A context‐dependent function of KLF4 could explain its protective versus pathogenic role in different type of vessels. While it confers vascular protection to atherothrombosis and pulmonary arterial hypertension when is upregulated in arteries (Zhou *et al*, 2012; Shatat *et al*, 2014), it may play a causative role in the development of venous‐derived CCM cavernomas (Boulday *et al*, 2011; Maddaluno *et al*, 2013). Albeit we still do not know the molecular basis of these discrepancies, it is conceivable that the interaction with cell‐specific transcription factors or differences in kinetics and levels of expression may modulate the response of ECs to KLF4. In this direction, it is noticeable that the levels of KLF4 are higher in retinal veins than in arteries in both physiological condition and upon *Ccm1* deletion. The cell specificity of KLF4‐dependent biological responses is also underlined by the observation that a comparable increase in KLF4 induced in ECs of different organs by loss of *Ccm1* resulted in a full EndMT switch only in brain‐derived ECs. Thus, cell context‐dependent function of KLF4 could be placed in a central position in promoting either vascular health or disease.

In the present paper, we also investigated the mechanism through which the absence of CCM may increase KLF4. Laminar flow through ERK5 activation is a well‐known inducer of *Klf4* expression and activity (Ohnesorge *et al*, 2010; Clark *et al*, 2011). However, we observed a marked increase of *Klf4* in CCM‐null ECs in the absence of flow. CCM complex disruption in the endocardium modulates the MEKK3‐MEK5‐ERK5 axis to upregulate *Klf4* and *Klf2* expression resulting in mid‐gestation heart failure (Zhou *et al*, 2015). MEKK3, identified as a CCM2 binding partner in the osmosensing complex (Uhlik *et al*, 2003), activates MEK5 that, in turn, through ERK5 dual phosphorylation, induces transcription factors MEF2A, MEF2C, and MEF2D to regulate their target genes (Kato *et al*, 1997; Sohn *et al*, 2005).

In agreement with these reports, we found that MEKK3‐MEK5 axis promotes ERK5 activation upon loss of CCM1 and inhibition of ERK5 prevents KLF4 upregulation and EndMT induction in cultured CCM1 KO ECs. These data support the concept that ERK5 is a key regulator of *Klf4* expression after CCM complex disruption. Importantly, ERK5‐dependent KLF4 upregulation is induced by inactivation of any one of the three *Ccm* genes consistent with the comparable phenotype observed in patients.

Phosphorylated ERK5 promoted *Klf4* expression largely through MEF2A and MEF2C transcriptional activity. However, since a complete KLF4 downregulation was not observed when these two transcription factors were silenced, either ERK5 alone or other ERK5 downstream transcription factors could be responsible for KLF4 upregulation (Nithianandarajah‐Jones *et al*, 2012). Moreover, since several signaling pathways are reported to contribute to CCM, it would interesting to investigate whether other transcription factors could have a crosstalk with the MEKK3‐MEK5‐ERK5‐MEF2‐KLF4 axis during the development and progression of cavernomas (Goitre *et al*, 2010; Stockton *et al*, 2010; Faurobert *et al*, 2013; Bravi *et al*, 2015; Gibson *et al*, 2015).

KLF2 has a causative role in the cardiac malformations observed in the absence of CCM in mouse and zebra fish embryos. In these models, KLF2 is responsible for both digestion of the cardiac jelly (Zhou *et al*, 2015) and increased angiogenesis due to β1integrin–KLF2‐egfl7 signaling pathway (Renz *et al*, 2015). In our CCM disease model, we found that *Klf2* was upregulated in freshly isolated brain‐derived CCM1‐null ECs or in cultured CCM1 KO ECs (data not shown), supporting the idea of a possible cooperation between KLF2 and KLF4. However, although these two transcription factors have many common targets, they still exhibit specificity due to individual differences in their affinities for jointly regulated promoters. The lack of a fully overlapping biological role of KLF2 and KLF4 is also demonstrated by the different phenotype of the respective null mice. While *Klf2*‐null mice exhibit abnormal blood vessel formation resulting in embryonic hemorrhage and death (Kuo *et al*, 1997), *Klf4*‐null mice die within 1 day after birth due to loss of skin barrier function (Segre *et al*, 1999). The relative contribution of KLF2 to the development of CCM remains an open question and requires future studies, since *Klf2* was not further upregulated upon *Klf4* ablation and apparently did not compensate for the lack of KLF4 in inducing EndMT (data not shown).

Taken together, the data presented here demonstrate that KLF4 is a master regulator of EndMT in CCM pathology; thus, the inhibition of *Klf4* expression may have a therapeutic role in limiting CCM appearance and progression. Transcription factors are considered, in general, “undruggable agents”. Indeed, KLF4 is a member of the large family of Kruppel‐like factors that are highly homologous sharing a triple zinc finger DNA‐binding domain (McConnell & Yang, 2010). The functions of the KLF family members can be exclusive or in some cases overlapping and redundant, compensating for one another and this might impair the efficacy of a possible therapy based on specific chemical compounds (Tetreault *et al*, 2013). Moreover, possible adverse effects such as increased sensitivity to inflammation and atherosclerosis should be considered. The use of either KLF4 or ERK5 bound to nanoparticles targeting CCM vascular lesions may be a more specific approach in the future.

## Materials and Methods

### Antibodies

For Western blotting and immunostainings, the following antibodies were used: VE‐CADHERIN rat (550548, BD Biosciences); VE‐CADHERIN goat (sc‐6458, Santa Cruz); PECAM1 hamster (MAB1398Z, Millipore); PECAM1 rat (553370, BD); PECAM1 rabbit (ab28364, Abcam); mKLF4 goat (AF3158, R&D); hKLF4 goat (AF3640, R&D); FSP1 rabbit (07‐2274, Millipore); ID1 rabbit (BCH‐1/37‐2, BIOCHECK); SCA1 rat (ab51317, Abcam); hCLAUDIN5 rabbit (ab53765, Abcam); pSMAD1/5 rabbit (9516, Cell Signaling); SMAD1 rabbit (9644, Cell Signaling); Glucose transporter type 1 (GLUT1) rabbit (RB9052P1, Thermo Scientific); ERK5 rabbit (07‐039, Upstate); VE‐PTP rabbit (produced and purified by New England Peptide); MEKK3 rabbit (5727, Cell Signaling); MEK5 mouse (610957, BD); BMP6 sheep (LS‐C150156, LS‐BIO); GAPDH mouse (SC‐32233, Santa Cruz); tubulin mouse (T9026, Sigma); vinculin mouse (V9264, Sigma); horseradish peroxidase (HRP)‐linked anti‐mouse, anti‐rat, and anti‐rabbit (Cell Signaling); and HRP‐linked anti‐goat (Promega). Biotin‐conjugated isolectin B4 (Vector Lab) was used to identify retinal vasculature. ALEXA FLUOR 488, 555, and 647 donkey secondary antibodies were from Life Technologies.

### Mouse lines

Mice were all bred on a C57BL/6 background. *Ccm1* ^fl/fl^, *Ccm2* ^fl/fl^, and *Ccm3* ^fl/fl^ mouse strains were already described (Boulday *et al*, 2011; Maddaluno *et al*, 2013; Bravi *et al*, 2015). *Klf4* ^fl/fl^ mice (Katz *et al*, 2005) were kindly donated by G.K. Owens. The *Cdh5(PAC)*‐CreERT2 mouse line (Wang *et al*, 2010) was kindly provided by R.H. Adams. Rosa26‐Stopfl‐LacZ mice (Srinivas *et al*, 2001) have been kindly donated by S. Casola. To obtain the iCCM1 mice, *Cdh5(PAC)*‐CreERT2 animals were first bred with the *Ccm1* ^fl/fl^ mouse strain to obtain tamoxifen‐inducible endothelial‐specific expression of Cre‐recombinase and *Ccm1* gene recombination. The *Cdh5(PAC)*‐CreERT2; *Ccm1* ^fl/fl^ mice were then crossed with Rosa26‐Stopfl‐LacZ animals to monitor the activity of Cre‐recombinase through the expression of enhanced yellow fluorescent protein (EYFP). To generate double iCCM1/KLF4 mice, *Cdh5(PAC)*‐CreERT2; *Ccm1* ^fl/fl^; Rosa26‐Stopfl‐LacZ mice were crossed with *Klf4* ^fl/fl^ animals. Deletion of floxed exons by Cre‐recombinase produces loss of function. Both iCCM2 and iCCM3 mice were derived as described for iCCM1 animals. iKLF4 mice were generated by crossing *Cdh5(PAC)*‐CreERT2 mice with *Klf4* ^fl/fl^ animals. For tamoxifen‐induced gene ablation, tamoxifen (T5648, Sigma) was dissolved in corn oil‐10% ethanol at 10 mg/ml and then further diluted at 2 mg/ml in corn oil (Pitulescu *et al*, 2010). Pups of the strains indicated in each experimental procedure, without any gender distinction, were injected at P1 with an intragastric injection of 100 μg (Pitulescu *et al*, 2010) and analyzed within 13 days from the gene recombination. The number of mice of each genotype analyzed in each experiment was specified in the figure legends. All the mouse strains were housed in specific pathogen‐free conditions.

### Endothelial cell isolation and culture

Cultured WT, CCM1 KO, CCM2 KO, CCM3 KO, and KLF4 KO lung‐derived ECs obtained from the respective floxed mouse strains (see mouse line paragraph), as well as brain microvascular fragments from WT, *Ccm1* ^fl/fl^, and *Ccm1* ^fl/fl^ *Klf4* ^fl/fl^ mice, were derived and cultured following standard protocols already used in different publications from our laboratory and other vascular biology groups (Bussolino *et al*, 1991; Calabria *et al*, 2006; Liebner *et al*, 2008; Anderberg *et al*, 2013). Lung‐derived ECs were isolated from the lung of two‐month‐old mice upon organ dissection and disaggregation with collagenase A (1.5 mg/ml; Roche) and DNase (25 μg/ml; Roche) in DMEM and immortalized with polyoma middle T antigen. Mouse brain microvascular fragments were recovered by brains of two‐month‐old mice rolled on Whatman 3MM chromatography blotting paper to remove the meninges, triturated, and digested 1 h at 37°C with 0.75% type 2 collagenase (Worthington Biochemical Corp.). The pellet was resuspended in a 25% w/v BSA solution and centrifuged for 20 min at 2,600 rpm at 4°C to obtain a microvessel‐enriched cell pellet. Then, the pellet was further digested in 10 mg/ml collagenase/dispase (Roche) and 1 μg/ml DNase I (Roche) for 15 min at 37°C. Capillary fragments were seeded in culture medium and selected for puromycin (4 μg/ml) resistance for 2 days. *In vitro* staining of endothelial cell‐specific molecules has been extensively performed in these EC monolayers of different origin, showing that endothelial cultures are 100% pure (Maddaluno *et al*, 2013). *Ccm1, Ccm2,* and *Klf4* floxed genes were ablated by treating cultured ECs with TAT‐Cre‐recombinase *in vitro* (Liebner *et al*, 2008). Recombination of the floxed *Ccm3* gene was induced by treating the cells at culture day 1 with the AdenoCre viral vector, as previously described (Cattelino *et al*, 2003). Immortalized cerebral microvascular EC of human origin hCMEC/D3 ECs were provided by P.O. Couraud (Weksler *et al*, 2005). For *ex vivo* experiments, freshly isolated Pecam1^+^ brain‐derived ECs from iCCM1, iCCM1/KLF4 mice, and matched controls (P12) were derived and processed as previously described (Maddaluno *et al*, 2013) without to be exposed to any culture condition. In order to obtain both lung‐ and spleen‐derived Pecam1^+^ ECs from iCCM1 and matched controls, the organs were disaggregated with collagenase A plus DNase (1.5 mg/ml and 25 μg/ml, respectively; Roche) for 2 h at 37°C and passed through a 70‐μm cell strainer before to be incubated for 45 min at 4°C with anti‐mouse Pecam1‐covered dynabeads. Heart‐derived Pecam1^+^ ECs from the same animals were recovered by incubating minced hearts with type I collagenase (2 mg/ml, Sigma) for 45 min at 37°C. A trituration of the heart suspension using a 30‐cc syringe is required before pipetting the cell suspension through a 70‐μm cell strainer and loading it on anti‐mouse Pecam1‐covered dynabeads.

### Cell treatments

XMD8‐92 (SelleckBio) and BIX‐02189 (SelleckBio) were dissolved in dimethylsulfoxide (DMSO). Confluent monolayers of ECs were grown in complete media (MCDB‐131 with 20% FBS) and treated daily with either 5 μM XMD8‐92 (for 72 h), 10 μM BIX‐02189 (for 48 h), or vehicle only. For BMP6 (R&D) treatment, ECs were grown in MCDB‐131 with 5% FBS and stimulated (100 ng/ml) either for 4 h in order to analyze SMAD1 phosphorylation or for 96 h for EndMT marker evaluation.

### Production of lentiviruses

Both shRNAs against murine *Klf4* and *Bmp6* and control non‐targeting shRNA cloned into pLKO.1 puro‐based lentiviral vector were purchased from Sigma‐Aldrich (St Louis, MO, USA, TRCN0000238250 and TRCN0000065652). The cDNA coding for the wild‐type mouse *Klf4* was obtained by GeneCopoeia (Rockville, USA) and subcloned into the lentiviral expression vector pLenti‐III‐HA (provided by abm, Richmond, BC, Canada) using XhoI and EcoRI restriction sites (Promega, Fitchburg, WI, USA). The lentiviral particles were produced in 293T cells using a four‐plasmid transfection system mediated by calcium phosphate, as previously described (Taulli *et al*, 2005). Lentivirus‐containing supernatants were collected 48 and 72 h after transfection, passed through a 0.45‐μM filter, and concentrated using PEG. Two consecutive cycles of lentiviral infection were carried out in the presence of polybrene followed by selection with 3 μg/ml puromycin for 72 h and maintenance with 1.5 μg/ml puromycin.

### Cell transfection and RNA interference

To perform RNA interference, the following siRNA were used: Stealth RNAi directed to mouse *Ccm1* (MSS294386, Life Technologies), Stealth RNAi directed to human *CCM1* (MSS234455, Life Technologies), ON‐TARGET plus SMART pool directed to mouse *Ccm2* (L‐057315‐00‐0005, Dharmacon), Stealth RNAi directed to mouse *Ccm3* (MSS249998, Life Technologies), Stealth RNAi directed to mouse *Erk5* (siERK5#1 MSS215825 and siERK5#2 MSS215827, Life Technologies), Stealth RNAi directed to mouse *Mek5* (MSS215820), Stealth RNAi directed to mouse *Mekk3* (siMEKK3#1 MSS218528 and siMEKK3#2 MSS218529), Stealth RNAi directed to mouse *Mef2A* (MSS206607), Stealth RNAi directed to mouse *Mef2C* (MSS206611), Stealth RNAi directed to mouse *Mef2D* (MSS247431), negative control siRNA Medium GC content (12935‐300, Life Technologies), and ON‐TARGET plus siControl (D‐001810‐10, Dharmacon). Cells were transfected with 40 nM siRNA oligonucleotides for 5 h in Optimem using Lipofectamine 2000 (Life Technologies) in accordance with the manufacturer's instructions and as previously described (Lampugnani *et al*, 2010).

### Western blotting

For Western blot analysis, cells were lysed using a boiling modified Laemmli sample buffer (2% SDS, 20% glycerol, and 125 mM Tris–HCl, pH 6.8). Protein concentration was estimated using the BCA Protein Assay Kit (Pierce). Equal amounts of proteins were separated by SDS–PAGE and transferred to a Protran nitrocellulose membrane 0.2‐μm pores (Whatman). Membranes were blocked for 1 h at room temperature (RT) and, then, incubated with primary antibody (overnight at 4°C) and HRP‐linked secondary antibodies (1 h at RT) in Tris‐buffered saline‐Tween (TBST) containing 5% BSA. Specific signals were detected by chemiluminescence system (GE Healthcare) using ChemiDoc XRS gel imaging system (Bio‐Rad). All Western blots shown in figures are representative experiments out of three performed. The predicted molecular weights are indicated in the figures.

### qRT–PCR analysis

Total RNA was isolated by extraction with the RNeasy mini kit (Qiagen). Isolation of RNA from freshly isolated Pecam^+^ brain‐derived ECs was performed using RNeasy micro kit (Qiagen). About 1 μg RNA was reverse‐transcribed with random hexamers (High Capacity cDNA Archive kit; Applied Biosystems). cDNA was amplified with the TaqMan Gene Expression assay (Applied Biosystems) and an ABI/Prism 7900 HT thermocycler. For any sample, the expression level normalized to both the housekeeping genes encoding *18S* and hypoxanthine phosphoribosyltransferase 1 (*Hprt1*) was determined by the comparative threshold cycle (*C*_t_) method as described previously (Spagnuolo *et al*, 2004). mRNA levels of the several transcripts of interest obtained by freshly isolated tissue‐derived ECs were normalized for endothelial cell content using *Pecam1* amount.

### Cell migration assay

Wound healing assay was used to measure cell migration. Confluent EC monolayers were starved overnight and then manually wounded with a pipette tip. Cells were then washed with phosphate‐buffered saline (PBS) and incubated at 37°C in complete media. Cell migration was examined by crystal violet staining (0.5% crystal violet in 20% methanol) and phase‐contrast microscopy. Quantification of migration was performed by ImageJ software.

### Cell proliferation assay

Cell proliferation assay was performed as already described (Giampietro *et al*, 2012). Briefly, cells were plated at density of 4,000 cells/well in 96‐well plates and analyzed at the indicated time points by crystal violet staining (0.1% crystal violet in 20% methanol). The optical density of the dye was measured using a Victor Microplate reader at the wavelength of 590 nm.

### Transcription factor binding site analysis and ChIP assay

The identification of KLF4 consensus binding sequences on the putative *Fsp1*,* Sca1*,* Id1*,* Bmp6* promoter regions was performed using the program MatInspector (Cartharius *et al*, 2005) and analyzing 5.0 kb upstream to 1.0 kb downstream from tss. ChIP assay was performed as already reported (Corada *et al*, 2010; Taddei *et al*, 2008). Chromatin extracts containing 1 mg of DNA fragments with an average size of 500 bp were incubated with either 10 mg KLF4 antibody or IgG control overnight at 4°C in the presence of protein G‐covered magnetic beads (Life Technologies). Bound DNA fragments were eluted and amplified by qRT–PCR using primers flanking the indicated promoter regions listed in Appendix Table S1. For qRT–PCR analyses, DNA was diluted with specific primers (10 mM each) to a final volume of 25 μl in SYBR green Reaction Mix (PerkinElmer).

### Transcriptional reporter assay

HEK‐293 cells were plated in 24‐well plates and transfected for 5 h in Optimem using Lipofectamine 2000 (Life Technologies) with the reporter plasmids (PGL3 basic vector) expressing the luciferase cDNA under the control of a promoter portion, identified as enriched in KLF4 binding sites by ChIP, of either *Fsp1* (1.8 kb), *Sca1* (2.1 kb), or *Bmp6* (2.3 kb). The sequences of the primers used to clone the promoter regions of *Fsp1, Sca1,* and *Bmp6* are listed in Appendix Table S2. pCDNA3 expression vectors containing either full‐length KLF4 or a mutant KLF4 lacking the DNA‐binding zinc finger domains (KLF4 ∆ZnF) (kind gifts of Mukesh Jain; Feinberg *et al*, 2005) were transfected where indicated. Renilla‐expressing plasmid was used as an internal control to correct for transfection efficiency. DualLuciferase^®^ Reporter Assay System (Promega) was used to measure firefly and renilla luciferase activities 30 h upon transfection with a GloMax^®^ luminometer (Promega).

### Brain tissue histology and immunofluorescence

Mouse brains were dissected, embedded in Tissue‐Tek OCT (Sakura), and snap‐frozen. Ten‐micrometer‐thick sections were fixed in 4% paraformaldehyde (PAF) and subjected to immunostaining. The slides were blocked for 3 h at RT with PBS containing 2% BSA, 5% donkey serum, and 0.05% Triton X‐100 (blocking solution). Incubation with primary (overnight at 4°C) and secondary (3 h at RT) antibodies was carried out in blocking solution. Sections were then counterstained with DAPI and mounted in Vectashield. Stained samples were analyzed with a Leica TCS AOBS confocal microscope using the following objectives: 63× (HCX PL APO lbd.BL NA = 1.4, oil), 40× (HCX PL APO NA = 1.25, oil), or 20× (HC PL FLUOTAR NA = 0.5, dry). Leica Confocal Software was used for the acquisition of images. The figures were assembled using Adobe Photoshop and Adobe Illustrator. The adjustments used in the preparation of the figures were for brightness, contrast, and background noise (blur filter).

For lesion quantifications, brains were fixed in 3% PAF overnight at 4°C and then embedded in 4% low‐melting‐point agarose. Serial sagittal sections (150 μm) of the cerebellum were obtained using a vibratome (1000 Plus, The Vibratome Company, St. Louis, MO, US). Each section was stained for Glut1 for vessel detection and examined under confocal microscopy (10× and 20× objectives). Analysis of the number/size of the CCM cavernae was performed using ImageJ software. Three different groups of caverns were defined according to their size (area in μm^2^) (Boulday *et al*, 2011). For immunohistochemistry on human samples, 3‐μm paraffin sections were stained with hematoxylin and eosin (H&E) to assess the histological features. Immunohistochemical analysis for CLAUDIN5 and KLF4 was performed on serial sections. Paraffin was removed with xylene, and the sections were rehydrated in graded alcohol. Antigen retrieval was carried out using preheated sodium citrate solution for 50 min. Tissue sections were blocked with FBS in PBS for 60 min and incubated overnight with primary antibodies. HRP‐Polymer Kit (Biocare Medical, Pike Lane Concord, CA, USA) was used for the detection of antigens followed by a diaminobenzidine chromogen reaction (Peroxidase substrate kit, DAB, SK‐4100; Vector Lab). All sections were counterstained with Mayer's hematoxylin. Images were acquired using an upright microscope (Olimpus BX51) using a 20× objective (UPlanFL N 20× NA = 0.5). NIS Elements software was used for image analysis. BMP6 and PECAM1 immunohistochemistry on murine brain samples were performed as described above for CLAUDIN5 and KLF4. Antigen retrival was carried out using sodium citrate for BMP6 and EDTA for PECAM1.

### Retinal immunohistochemistry

Retina immunostaining was carried out with littermates processed simultaneously under the same conditions. The retinae were dissected from eyes fixed in 2% PFA for 2 h at 4°C. After blocking/permeabilization in 1% BSA, 5% donkey serum and 0.5% Triton X‐100 in PBS overnight, the retinas were washed in Pblec Buffer (1% Triton X‐100, 1 mM CaCl_2_, 1 mM MgCl_2_, and 0.1 mM MnCl_2_ in PBS, pH 6.8) and incubated overnight at 4° C in Pblec buffer containing biotinylated isolectin B4 (IB4) and the primary antibodies. Then, the retinae were incubated with fluorophore‐conjugated antibodies and with Alexa Fluor 488/555/647 streptavidin (Life Technologies) and mounted with ProLong Gold (Life Technologies). Confocal analysis was performed using a Leica TCS AOBS or Leica TCS SP5 confocal microscope using the following objectives: 40× (HCX PL APO NA = 1.25, oil) or 10× (HC PL FLUOTAR NA = 0.3, dry). Leica Confocal Software was used for the acquisition. The figures were prepared as described in “brain tissue histology and immunofluorescence” section.

The vessel front density and vascular progression in the retinae were performed as previously described (Corada *et al*, 2013). All image analyses were carried out using the ImageJ software.

### Statistics

Data are expressed as mean ± SD. Student's two‐tailed non‐paired *t*‐tests were used to determine the statistical significance for the *in vitro, ex vivo,* and *in vivo* analyses. The significance level was set at *P *< 0.05. For survival experiment, Kaplan–Meier curves were analyzed with the Mantel–Cox test.

### Study approval

All experimental animal procedures and the mouse handling described in this study were in full accordance with the guidelines established in the Principles of Laboratory Animal Care (directive 86/609/EEC) and approved by the Italian Ministry of Health (project 19/12).

Human brain specimens from familial CCM1 patients were obtained from Angioma Alliance BioBank upon we got the written permission of the Angioma Alliance BioBank Scientific Advisory Board. Informed consent was obtained for all samples linked with clinical data. All tissue samples were collected and used for standardized operative procedures approved by the IFOM Ethics Committee for Biomedical research with the register number 2/14.

## Author contributions

RC and NR designed and performed *in vitro*,* ex vivo,* and *in vivo* experiments, analyzed the results, and wrote the manuscript; LB contributed to the *in vivo* stainings; MC performed dissection of the retinae; EP subcloned cDNA coding for *Klf4* into lentiviral expression vector; CG and LM contributed to the scientific discussion; MFM provided expertise on ChIP; NB and MS provided help during manuscript revision; RHA and MKJ contributed to the scientific discussion and provided critical comments to the manuscript; MKJ shared also pCDNA3‐KLF4 and pCDNA3‐KLF4 ∆ZnF constructs. GKO provided the *Klf4* ^fl/fl^ mice; MGL contributed to the scientific discussion and provided critical comments to the manuscript; ED directed the research project, analyzed the data, and wrote the manuscript.

## Conflict of interest

The authors declare that they have no conflict of interest.

**The paper explained**

**Problem**

Cerebral cavernous malformation (CCM) is a genetic disease characterized by vascular malformations of brain microvessels that result in neurological signs such as headache, seizures, paralyses, and hemorrhagic stroke. The only therapy available is surgery that is, however, often hazardous depending on the location of the vascular malformation. CCM is caused by the loss‐of‐function mutations of any of three independent genes, known as *CCM1*,* CCM2,* and *CCM3*. We have recently reported that the TGFβ/BMP signaling pathway induces an endothelial‐to‐mesenchymal transition (EndMT) in *Ccm*‐deficient ECs that, in turn, contributes to the development and progression of cavernomas. However, the molecular basis of the activation of such signaling route has not yet been clarified.

**Results**

Here, we identify KLF4 as a master regulator of EndMT during CCM development and progression. Loss of *Ccm1* leads to MEKK3‐MEK5‐dependent ERK5 phosphorylation that, in turn, induces a strong upregulation of *Klf4* expression in brain endothelial cells. The resulting increased KLF4 transcriptional activity is responsible for the EndMT switch observed in CCM1‐null ECs, since it is able to drive the expression of both molecules responsible for TGFβ/BMP signaling activation and a number of EndMT markers involved in CCM pathogenesis.

Importantly, *in vivo Klf4* genetic inactivation blocks the development and progression of cavernomas and almost completely prevents associated mortality.

**Impact**

Our dissection of the molecular mechanisms underlying cavernoma pathogenesis unveils KLF4 upregulation as the key initial event in CCM. In fact, *Klf4* gene ablation almost abolishes the appearance of lesions and mortality in endothelial‐specific CCM1‐null mice. We thus suggest KLF4 to be an important novel therapeutic target for the pharmacological treatment of CCM, which is currently only treatable with surgical intervention.

## Supporting information



Appendix

Expanded View Figures PDF

Source Data for Expanded View and Appendix

Review Process File

Source Data for Figure 2

Source Data for Figure 3

Source Data for Figure 4

Source Data for Figure 5

Source Data for Figure 6

Source Data for Figure 7

Source Data for Figure 8

## References

[emmm201505433-bib-0001] Anderberg C , Cunha SI , Zhai Z , Cortez E , Pardali E , Johnson JR , Franco M , Paez‐Ribes M , Cordiner R , Fuxe J *et al* (2013) Deficiency for endoglin in tumor vasculature weakens the endothelial barrier to metastatic dissemination. J Exp Med 210: 563–579 23401487 10.1084/jem.20120662 PMC3600899

[emmm201505433-bib-0002] Beraud‐Dufour S , Gautier R , Albiges‐Rizo C , Chardin P , Faurobert E (2007) Krit 1 interactions with microtubules and membranes are regulated by Rap1 and integrin cytoplasmic domain associated protein‐1. FEBS J 274: 5518–5532 17916086 10.1111/j.1742-4658.2007.06068.x PMC2580780

[emmm201505433-bib-0003] Bergametti F , Denier C , Labauge P , Arnoult M , Boetto S , Clanet M , Coubes P , Echenne B , Ibrahim R , Irthum B *et al* (2005) Mutations within the programmed cell death 10 gene cause cerebral cavernous malformations. Am J Hum Genet 76: 42–51 15543491 10.1086/426952 PMC1196432

[emmm201505433-bib-0004] Borikova AL , Dibble CF , Sciaky N , Welch CM , Abell AN , Bencharit S , Johnson GL (2010) Rho kinase inhibition rescues the endothelial cell cerebral cavernous malformation phenotype. J Biol Chem 285: 11760–11764 20181950 10.1074/jbc.C109.097220 PMC2852911

[emmm201505433-bib-0005] Boulday G , Rudini N , Maddaluno L , Blecon A , Arnould M , Gaudric A , Chapon F , Adams RH , Dejana E , Tournier‐Lasserve E (2011) Developmental timing of CCM2 loss influences cerebral cavernous malformations in mice. J Exp Med 208: 1835–1847 21859843 10.1084/jem.20110571 PMC3171098

[emmm201505433-bib-0006] Bravi L , Rudini N , Cuttano R , Giampietro C , Maddaluno L , Ferrarini L , Adams RH , Corada M , Boulday G , Tournier‐Lasserve E *et al* (2015) Sulindac metabolites decrease cerebrovascular malformations in CCM3‐knockout mice. Proc Natl Acad Sci USA 112: 8421–8426 26109568 10.1073/pnas.1501352112 PMC4500248

[emmm201505433-bib-0007] Bussolino F , De Rossi M , Sica A , Colotta F , Wang JM , Bocchietto E , Padura IM , Bosia A , DeJana E , Mantovani A (1991) Murine endothelioma cell lines transformed by polyoma middle T oncogene as target for and producers of cytokines. J Immunol 147: 2122–2129 1918946

[emmm201505433-bib-0008] Calabria AR , Weidenfeller C , Jones AR , de Vries HE , Shusta EV (2006) Puromycin‐purified rat brain microvascular endothelial cell cultures exhibit improved barrier properties in response to glucocorticoid induction. J Neurochem 97: 922–933 16573646 10.1111/j.1471-4159.2006.03793.x

[emmm201505433-bib-0009] Cartharius K , Frech K , Grote K , Klocke B , Haltmeier M , Klingenhoff A , Frisch M , Bayerlein M , Werner T (2005) MatInspector and beyond: promoter analysis based on transcription factor binding sites. Bioinformatics 21: 2933–2942 15860560 10.1093/bioinformatics/bti473

[emmm201505433-bib-0010] Cattelino A , Liebner S , Gallini R , Zanetti A , Balconi G , Corsi A , Bianco P , Wolburg H , Moore R , Oreda B *et al* (2003) The conditional inactivation of the beta‐catenin gene in endothelial cells causes a defective vascular pattern and increased vascular fragility. J Cell Biol 162: 1111–1122 12975353 10.1083/jcb.200212157 PMC2172846

[emmm201505433-bib-0011] Cavalcanti DD , Kalani MY , Martirosyan NL , Eales J , Spetzler RF , Preul MC (2012) Cerebral cavernous malformations: from genes to proteins to disease. J Neurosurg 116: 122–132 21962164 10.3171/2011.8.JNS101241

[emmm201505433-bib-0012] Chan AC , Drakos SG , Ruiz OE , Smith AC , Gibson CC , Ling J , Passi SF , Stratman AN , Sacharidou A , Revelo MP *et al* (2011) Mutations in 2 distinct genetic pathways result in cerebral cavernous malformations in mice. J Clin Invest 121: 1871–1881 21490399 10.1172/JCI44393 PMC3083782

[emmm201505433-bib-0013] Chen PY , Qin L , Barnes C , Charisse K , Yi T , Zhang X , Ali R , Medina PP , Yu J , Slack FJ *et al* (2012) FGF regulates TGF‐beta signaling and endothelial‐to‐mesenchymal transition via control of let‐7 miRNA expression. Cell Rep 2: 1684–1696 23200853 10.1016/j.celrep.2012.10.021 PMC3534912

[emmm201505433-bib-0014] Chen PY , Qin L , Tellides G , Simons M (2014) Fibroblast growth factor receptor 1 is a key inhibitor of TGFbeta signaling in the endothelium. Sci Signal 7: ra90 25249657 10.1126/scisignal.2005504

[emmm201505433-bib-0015] Clark PR , Jensen TJ , Kluger MS , Morelock M , Hanidu A , Qi Z , Tatake RJ , Pober JS (2011) MEK5 is activated by shear stress, activates ERK5 and induces KLF4 to modulate TNF responses in human dermal microvascular endothelial cells. Microcirculation 18: 102–117 21166929 10.1111/j.1549-8719.2010.00071.x PMC3075844

[emmm201505433-bib-0016] Clatterbuck RE , Eberhart CG , Crain BJ , Rigamonti D (2001) Ultrastructural and immunocytochemical evidence that an incompetent blood‐brain barrier is related to the pathophysiology of cavernous malformations. J Neurol Neurosurg Psychiatry 71: 188–192 11459890 10.1136/jnnp.71.2.188 PMC1737494

[emmm201505433-bib-0100] Corada M , Nyqvist D , Orsenigo F , Caprini A , Giampietro C , Taketo MM , Iruela‐Arispe ML , Adams RH , Dejana E (2010) The Wnt/beta‐catenin pathway modulates vascular remodeling and specification by upregulating Dll4/Notch signaling. Dev Cell 18: 938–949 20627076 10.1016/j.devcel.2010.05.006 PMC8127076

[emmm201505433-bib-0017] Corada M , Orsenigo F , Morini MF , Pitulescu ME , Bhat G , Nyqvist D , Breviario F , Conti V , Briot A , Iruela‐Arispe ML *et al* (2013) Sox17 is indispensable for acquisition and maintenance of arterial identity. Nat Commun 4: 2609 24153254 10.1038/ncomms3609 PMC3826640

[emmm201505433-bib-0018] Cowan CE , Kohler EE , Dugan TA , Mirza MK , Malik AB , Wary KK (2010) Kruppel‐like factor‐4 transcriptionally regulates VE‐cadherin expression and endothelial barrier function. Circ Res 107: 959–966 20724706 10.1161/CIRCRESAHA.110.219592 PMC3018700

[emmm201505433-bib-0019] Dekker RJ , van Soest S , Fontijn RD , Salamanca S , de Groot PG , VanBavel E , Pannekoek H , Horrevoets AJ (2002) Prolonged fluid shear stress induces a distinct set of endothelial cell genes, most specifically lung Kruppel‐like factor (KLF2). Blood 100: 1689–1698 12176889 10.1182/blood-2002-01-0046

[emmm201505433-bib-0020] Faurobert E , Rome C , Lisowska J , Manet‐Dupe S , Boulday G , Malbouyres M , Balland M , Bouin AP , Keramidas M , Bouvard D *et al* (2013) CCM1‐ICAP‐1 complex controls beta1 integrin‐dependent endothelial contractility and fibronectin remodeling. J Cell Biol 202: 545–561 23918940 10.1083/jcb.201303044 PMC3734079

[emmm201505433-bib-0021] Feinberg MW , Cao Z , Wara AK , Lebedeva MA , Senbanerjee S , Jain MK (2005) Kruppel‐like factor 4 is a mediator of proinflammatory signaling in macrophages. J Biol Chem 280: 38247–38258 16169848 10.1074/jbc.M509378200

[emmm201505433-bib-0022] Fisher OS , Boggon TJ (2013) Signaling pathways and the cerebral cavernous malformations proteins: lessons from structural biology. Cell Mol Life Sci 71: 1881–1892 24287896 10.1007/s00018-013-1532-9 PMC3999170

[emmm201505433-bib-0023] Garcia J , Sandi MJ , Cordelier P , Binetruy B , Pouyssegur J , Iovanna JL , Tournaire R (2012) Tie1 deficiency induces endothelial‐mesenchymal transition. EMBO Rep 13: 431–439 22421998 10.1038/embor.2012.29 PMC3343349

[emmm201505433-bib-0024] Garrett‐Sinha LA , Eberspaecher H , Seldin MF , de Crombrugghe B (1996) A gene for a novel zinc‐finger protein expressed in differentiated epithelial cells and transiently in certain mesenchymal cells. J Biol Chem 271: 31384–31390 8940147 10.1074/jbc.271.49.31384

[emmm201505433-bib-0025] Giampietro C , Taddei A , Corada M , Sarra‐Ferraris GM , Alcalay M , Cavallaro U , Orsenigo F , Lampugnani MG , Dejana E (2012) Overlapping and divergent signaling pathways of N‐cadherin and VE‐cadherin in endothelial cells. Blood 119: 2159–2170 22246030 10.1182/blood-2011-09-381012

[emmm201505433-bib-0026] Gibson CC , Zhu W , Davis CT , Bowman‐Kirigin JA , Chan AC , Ling J , Walker AE , Goitre L , Delle Monache S , Retta SF *et al* (2015) Strategy for identifying repurposed drugs for the treatment of cerebral cavernous malformation. Circulation 131: 289–299 25486933 10.1161/CIRCULATIONAHA.114.010403 PMC4356181

[emmm201505433-bib-0027] Glading A , Han J , Stockton RA , Ginsberg MH (2007) KRIT‐1/CCM1 is a Rap1 effector that regulates endothelial cell cell junctions. J Cell Biol 179: 247–254 17954608 10.1083/jcb.200705175 PMC2064761

[emmm201505433-bib-0028] Glading AJ , Ginsberg MH (2010) Rap1 and its effector KRIT1/CCM1 regulate beta‐catenin signaling. Dis Model Mech 3: 73–83 20007487 10.1242/dmm.003293 PMC2806902

[emmm201505433-bib-0029] Goitre L , Balzac F , Degani S , Degan P , Marchi S , Pinton P , Retta SF (2010) KRIT1 regulates the homeostasis of intracellular reactive oxygen species. PLoS ONE 5: e11786 20668652 10.1371/journal.pone.0011786 PMC2910502

[emmm201505433-bib-0030] Hale AT , Tian H , Anih E , Recio FO 3rd , Shatat MA , Johnson T , Liao X , Ramirez‐Bergeron DL , Proweller A , Ishikawa M *et al* (2014) Endothelial Kruppel‐like factor 4 regulates angiogenesis and the Notch signaling pathway. J Biol Chem 289: 12016–12028 24599951 10.1074/jbc.M113.530956 PMC4002108

[emmm201505433-bib-0031] Hamik A , Lin Z , Kumar A , Balcells M , Sinha S , Katz J , Feinberg MW , Gerzsten RE , Edelman ER , Jain MK (2007) Kruppel‐like factor 4 regulates endothelial inflammation. J Biol Chem 282: 13769–13779 17339326 10.1074/jbc.M700078200

[emmm201505433-bib-0032] James D , Nam HS , Seandel M , Nolan D , Janovitz T , Tomishima M , Studer L , Lee G , Lyden D , Benezra R *et al* (2010) Expansion and maintenance of human embryonic stem cell‐derived endothelial cells by TGFbeta inhibition is Id1 dependent. Nat Biotechnol 28: 161–166 20081865 10.1038/nbt.1605 PMC2931334

[emmm201505433-bib-0033] Kato Y , Kravchenko VV , Tapping RI , Han J , Ulevitch RJ , Lee JD (1997) BMK1/ERK5 regulates serum‐induced early gene expression through transcription factor MEF2C. EMBO J 16: 7054–7066 9384584 10.1093/emboj/16.23.7054 PMC1170308

[emmm201505433-bib-0034] Katz JP , Perreault N , Goldstein BG , Actman L , McNally SR , Silberg DG , Furth EE , Kaestner KH (2005) Loss of Klf4 in mice causes altered proliferation and differentiation and precancerous changes in the adult stomach. Gastroenterology 128: 935–945 15825076 10.1053/j.gastro.2005.02.022

[emmm201505433-bib-0035] Kitao A , Sato Y , Sawada‐Kitamura S , Harada K , Sasaki M , Morikawa H , Shiomi S , Honda M , Matsui O , Nakanuma Y (2009) Endothelial to mesenchymal transition via transforming growth factor‐beta1/Smad activation is associated with portal venous stenosis in idiopathic portal hypertension. Am J Pathol 175: 616–626 19608867 10.2353/ajpath.2009.081061 PMC2716961

[emmm201505433-bib-0036] Komaravolu RK , Adam C , Moonen JR , Harmsen MC , Goebeler M , Schmidt M (2015) Erk5 inhibits endothelial migration via KLF2‐dependent down‐regulation of PAK1. Cardiovasc Res 105: 86–95 25388666 10.1093/cvr/cvu236

[emmm201505433-bib-0037] Korchynskyi O , ten Dijke P (2002) Identification and functional characterization of distinct critically important bone morphogenetic protein‐specific response elements in the Id1 promoter. J Biol Chem 277: 4883–4891 11729207 10.1074/jbc.M111023200

[emmm201505433-bib-0038] Kuo CT , Veselits ML , Barton KP , Lu MM , Clendenin C , Leiden JM (1997) The LKLF transcription factor is required for normal tunica media formation and blood vessel stabilization during murine embryogenesis. Genes Dev 11: 2996–3006 9367982 10.1101/gad.11.22.2996 PMC316695

[emmm201505433-bib-0039] Labauge P , Denier C , Bergametti F , Tournier‐Lasserve E (2007) Genetics of cavernous angiomas. Lancet Neurol 6: 237–244 17303530 10.1016/S1474-4422(07)70053-4

[emmm201505433-bib-0040] Laberge‐le Couteulx S , Jung HH , Labauge P , Houtteville JP , Lescoat C , Cecillon M , Marechal E , Joutel A , Bach JF , Tournier‐Lasserve E (1999) Truncating mutations in CCM1, encoding KRIT1, cause hereditary cavernous angiomas. Nat Genet 23: 189–193 10508515 10.1038/13815

[emmm201505433-bib-0041] Lampugnani MG , Orsenigo F , Rudini N , Maddaluno L , Boulday G , Chapon F , Dejana E (2010) CCM1 regulates vascular‐lumen organization by inducing endothelial polarity. J Cell Sci 123: 1073–1080 20332120 10.1242/jcs.059329

[emmm201505433-bib-0042] Li DY , Whitehead KJ (2010) Evaluating strategies for the treatment of cerebral cavernous malformations. Stroke 41: S92–S94 20876517 10.1161/STROKEAHA.110.594929 PMC3409848

[emmm201505433-bib-0043] Li HX , Han M , Bernier M , Zheng B , Sun SG , Su M , Zhang R , Fu JR , Wen JK (2010) Kruppel‐like factor 4 promotes differentiation by transforming growth factor‐beta receptor‐mediated Smad and p38 MAPK signaling in vascular smooth muscle cells. J Biol Chem 285: 17846–17856 20375011 10.1074/jbc.M109.076992 PMC2878548

[emmm201505433-bib-0044] Liang SX , Khachigian LM , Ahmadi Z , Yang M , Liu S , Chong BH (2011) In vitro and in vivo proliferation, differentiation and migration of cardiac endothelial progenitor cells (SCA1+/CD31+ side‐population cells). J Thromb Haemost 9: 1628–1637 21615679 10.1111/j.1538-7836.2011.04375.x

[emmm201505433-bib-0045] Liebner S , Corada M , Bangsow T , Babbage J , Taddei A , Czupalla CJ , Reis M , Felici A , Wolburg H , Fruttiger M *et al* (2008) Wnt/beta‐catenin signaling controls development of the blood‐brain barrier. J Cell Biol 183: 409–417 18955553 10.1083/jcb.200806024 PMC2575783

[emmm201505433-bib-0046] Maddaluno L , Rudini N , Cuttano R , Bravi L , Giampietro C , Corada M , Ferrarini L , Orsenigo F , Papa E , Boulday G *et al* (2013) EndMT contributes to the onset and progression of cerebral cavernous malformations. Nature 498: 492–496 23748444 10.1038/nature12207

[emmm201505433-bib-0047] Maejima T , Inoue T , Kanki Y , Kohro T , Li G , Ohta Y , Kimura H , Kobayashi M , Taguchi A , Tsutsumi S *et al* (2014) Direct evidence for pitavastatin induced chromatin structure change in the KLF4 gene in endothelial cells. PLoS ONE 9: e96005 24797675 10.1371/journal.pone.0096005 PMC4010393

[emmm201505433-bib-0048] Magrini E , Villa A , Angiolini F , Doni A , Mazzarol G , Rudini N , Maddaluno L , Komuta M , Topal B , Prenen H *et al* (2014) Endothelial deficiency of L1 reduces tumor angiogenesis and promotes vessel normalization. J Clin Invest 124: 4335–4350 25157817 10.1172/JCI70683 PMC4191010

[emmm201505433-bib-0049] Maraire JN , Awad IA (1995) Intracranial cavernous malformations: lesion behavior and management strategies. Neurosurgery 37: 591–605 8559286 10.1227/00006123-199510000-00001

[emmm201505433-bib-0050] McConnell BB , Yang VW (2010) Mammalian Kruppel‐like factors in health and diseases. Physiol Rev 90: 1337–1381 20959618 10.1152/physrev.00058.2009 PMC2975554

[emmm201505433-bib-0051] McDonald DA , Shenkar R , Shi C , Stockton RA , Akers AL , Kucherlapati MH , Kucherlapati R , Brainer J , Ginsberg MH , Awad IA *et al* (2011) A novel mouse model of cerebral cavernous malformations based on the two‐hit mutation hypothesis recapitulates the human disease. Hum Mol Genet 20: 211–222 20940147 10.1093/hmg/ddq433 PMC3005897

[emmm201505433-bib-0052] Medici D , Shore EM , Lounev VY , Kaplan FS , Kalluri R , Olsen BR (2010) Conversion of vascular endothelial cells into multipotent stem‐like cells. Nat Med 16: 1400–1406 21102460 10.1038/nm.2252 PMC3209716

[emmm201505433-bib-0053] Medici D , Kalluri R (2012) Endothelial‐mesenchymal transition and its contribution to the emergence of stem cell phenotype. Semin Cancer Biol 22: 379–384 22554794 10.1016/j.semcancer.2012.04.004 PMC3422405

[emmm201505433-bib-0054] Mleynek TM , Chan AC , Redd M , Gibson CC , Davis CT , Shi DS , Chen T , Carter KL , Ling J , Blanco R *et al* (2014) Lack of CCM1 induces hypersprouting and impairs response to flow. Hum Mol Genet 23: 6223–6234 24990152 10.1093/hmg/ddu342 PMC4222362

[emmm201505433-bib-0055] Moriarity JL , Wetzel M , Clatterbuck RE , Javedan S , Sheppard JM , Hoenig‐Rigamonti K , Crone NE , Breiter SN , Lee RR , Rigamonti D (1999) The natural history of cavernous malformations: a prospective study of 68 patients. Neurosurgery 44: 1166–1171; discussion 1172–1163 10371615

[emmm201505433-bib-0056] Nithianandarajah‐Jones GN , Wilm B , Goldring CE , Muller J , Cross MJ (2012) ERK5: structure, regulation and function. Cell Signal 24: 2187–2196 22800864 10.1016/j.cellsig.2012.07.007

[emmm201505433-bib-0057] Ohnesorge N , Viemann D , Schmidt N , Czymai T , Spiering D , Schmolke M , Ludwig S , Roth J , Goebeler M , Schmidt M (2010) Erk5 activation elicits a vasoprotective endothelial phenotype via induction of Kruppel‐like factor 4 (KLF4). J Biol Chem 285: 26199–26210 20551324 10.1074/jbc.M110.103127 PMC2924030

[emmm201505433-bib-0058] Pitulescu ME , Schmidt I , Benedito R , Adams RH (2010) Inducible gene targeting in the neonatal vasculature and analysis of retinal angiogenesis in mice. Nat Protoc 5: 1518–1534 20725067 10.1038/nprot.2010.113

[emmm201505433-bib-0059] Reddy S , Gorin MB , McCannel TA , Tsui I , Straatsma BR (2010) Novel KRIT1/CCM1 mutation in a patient with retinal cavernous hemangioma and cerebral cavernous malformation. Graefe's Arch Clin Exp Ophthalmol 248: 1359–1361 20306072 10.1007/s00417-010-1329-6 PMC2910301

[emmm201505433-bib-0060] Renz M , Otten C , Faurobert E , Rudolph F , Zhu Y , Boulday G , Duchene J , Mickoleit M , Dietrich AC , Ramspacher C *et al* (2015) Regulation of beta1 Integrin‐Klf2‐Mediated Angiogenesis by CCM Proteins. Dev Cell 32: 181–190 25625207 10.1016/j.devcel.2014.12.016

[emmm201505433-bib-0061] Riant F , Bergametti F , Ayrignac X , Boulday G , Tournier‐Lasserve E (2010) Recent insights into cerebral cavernous malformations: the molecular genetics of CCM. FEBS J 277: 1070–1075 20096038 10.1111/j.1742-4658.2009.07535.x

[emmm201505433-bib-0062] Riant F , Cecillon M , Saugier‐Veber P , Tournier‐Lasserve E (2013) CCM molecular screening in a diagnosis context: novel unclassified variants leading to abnormal splicing and importance of large deletions. Neurogenetics 14: 133–141 23595507 10.1007/s10048-013-0362-0

[emmm201505433-bib-0063] Rigamonti D , Hadley MN , Drayer BP , Johnson PC , Hoenig‐Rigamonti K , Knight JT , Spetzler RF (1988) Cerebral cavernous malformations. Incidence and familial occurrence. N Engl J Med 319: 343–347 3393196 10.1056/NEJM198808113190605

[emmm201505433-bib-0064] Segre JA , Bauer C , Fuchs E (1999) Klf4 is a transcription factor required for establishing the barrier function of the skin. Nat Genet 22: 356–360 10431239 10.1038/11926

[emmm201505433-bib-0065] Shatat MA , Tian H , Zhang R , Tandon G , Hale A , Fritz JS , Zhou G , Martinez‐Gonzalez J , Rodriguez C , Champion HC *et al* (2014) Endothelial Kruppel‐like factor 4 modulates pulmonary arterial hypertension. Am J Respir Cell Mol Biol 50: 647–653 24156273 10.1165/rcmb.2013-0135OC PMC4068930

[emmm201505433-bib-0066] Shi Y , Wang YF , Jayaraman L , Yang H , Massague J , Pavletich NP (1998) Crystal structure of a Smad MH1 domain bound to DNA: insights on DNA binding in TGF‐beta signaling. Cell 94: 585–594 9741623 10.1016/s0092-8674(00)81600-1

[emmm201505433-bib-0067] Shields JM , Christy RJ , Yang VW (1996) Identification and characterization of a gene encoding a gut‐enriched Kruppel‐like factor expressed during growth arrest. J Biol Chem 271: 20009–20017 8702718 10.1074/jbc.271.33.20009 PMC2330254

[emmm201505433-bib-0068] Sohn SJ , Li D , Lee LK , Winoto A (2005) Transcriptional regulation of tissue‐specific genes by the ERK5 mitogen‐activated protein kinase. Mol Cell Biol 25: 8553–8566 16166637 10.1128/MCB.25.19.8553-8566.2005 PMC1265748

[emmm201505433-bib-0069] Spagnuolo R , Corada M , Orsenigo F , Zanetta L , Deuschle U , Sandy P , Schneider C , Drake CJ , Breviario F , Dejana E (2004) Gas1 is induced by VE‐cadherin and vascular endothelial growth factor and inhibits endothelial cell apoptosis. Blood 103: 3005–3012 15070677 10.1182/blood-2003-07-2459

[emmm201505433-bib-0070] Srinivas S , Watanabe T , Lin CS , William CM , Tanabe Y , Jessell TM , Costantini F (2001) Cre reporter strains produced by targeted insertion of EYFP and ECFP into the ROSA26 locus. BMC Dev Biol 1: 4 11299042 10.1186/1471-213X-1-4 PMC31338

[emmm201505433-bib-0071] Stockton RA , Shenkar R , Awad IA , Ginsberg MH (2010) Cerebral cavernous malformations proteins inhibit Rho kinase to stabilize vascular integrity. J Exp Med 207: 881–896 20308363 10.1084/jem.20091258 PMC2856024

[emmm201505433-bib-0101] Taddei A , Giampietro C , Conti A , Orsenigo F , Breviario F , Pirazzoli V , Potente M , Daly Daly , Dimmeler S , Dejana E (2008) Endothelial adherens junctions control tight junctions by VE‐cadherin‐mediated upregulation of claudin‐5. Nat Cell Biol 10: 923–934 18604199 10.1038/ncb1752

[emmm201505433-bib-0072] Tatake RJ , O'Neill MM , Kennedy CA , Wayne AL , Jakes S , Wu D , Kugler SZ Jr , Kashem MA , Kaplita P , Snow RJ (2008) Identification of pharmacological inhibitors of the MEK5/ERK5 pathway. Biochem Biophys Res Commun 377: 120–125 18834865 10.1016/j.bbrc.2008.09.087

[emmm201505433-bib-0073] Taulli R , Accornero P , Follenzi A , Mangano T , Morotti A , Scuoppo C , Forni PE , Bersani F , Crepaldi T , Chiarle R *et al* (2005) RNAi technology and lentiviral delivery as a powerful tool to suppress Tpr‐Met‐mediated tumorigenesis. Cancer Gene Ther 12: 456–463 15719029 10.1038/sj.cgt.7700815

[emmm201505433-bib-0074] Tetreault MP , Yang Y , Katz JP (2013) Kruppel‐like factors in cancer. Nat Rev Cancer 13: 701–713 24060862 10.1038/nrc3582

[emmm201505433-bib-0075] Tomlinson FH , Houser OW , Scheithauer BW , Sundt TM Jr , Okazaki H , Parisi JE (1994) Angiographically occult vascular malformations: a correlative study of features on magnetic resonance imaging and histological examination. Neurosurgery 34: 792–799; discussion 799–800 8052376 10.1227/00006123-199405000-00002

[emmm201505433-bib-0076] Uhlik MT , Abell AN , Johnson NL , Sun W , Cuevas BD , Lobel‐Rice KE , Horne EA , Dell'Acqua ML , Johnson GL (2003) Rac‐MEKK3‐MKK3 scaffolding for p38 MAPK activation during hyperosmotic shock. Nat Cell Biol 5: 1104–1110 14634666 10.1038/ncb1071

[emmm201505433-bib-0077] Wang Y , Nakayama M , Pitulescu ME , Schmidt TS , Bochenek ML , Sakakibara A , Adams S , Davy A , Deutsch U , Luthi U *et al* (2010) Ephrin‐B2 controls VEGF‐induced angiogenesis and lymphangiogenesis. Nature 465: 483–486 20445537 10.1038/nature09002

[emmm201505433-bib-0078] Wang Y , Yang C , Gu Q , Sims M , Gu W , Pfeffer LM , Yue J (2015) KLF4 Promotes Angiogenesis by Activating VEGF Signaling in Human Retinal Microvascular Endothelial Cells. PLoS ONE 10: e0130341 26075898 10.1371/journal.pone.0130341 PMC4467843

[emmm201505433-bib-0079] Weksler BB , Subileau EA , Perriere N , Charneau P , Holloway K , Leveque M , Tricoire‐Leignel H , Nicotra A , Bourdoulous S , Turowski P *et al* (2005) Blood‐brain barrier‐specific properties of a human adult brain endothelial cell line. FASEB J 19: 1872–1874 16141364 10.1096/fj.04-3458fje

[emmm201505433-bib-0080] Whitehead KJ , Chan AC , Navankasattusas S , Koh W , London NR , Ling J , Mayo AH , Drakos SG , Jones CA , Zhu W *et al* (2009) The cerebral cavernous malformation signaling pathway promotes vascular integrity via Rho GTPases. Nat Med 15: 177–184 19151728 10.1038/nm.1911 PMC2767168

[emmm201505433-bib-0081] Wustehube J , Bartol A , Liebler SS , Brutsch R , Zhu Y , Felbor U , Sure U , Augustin HG , Fischer A (2010) Cerebral cavernous malformation protein CCM1 inhibits sprouting angiogenesis by activating DELTA‐NOTCH signaling. Proc Natl Acad Sci USA 107: 12640–12645 20616044 10.1073/pnas.1000132107 PMC2906569

[emmm201505433-bib-0082] Yang Q , Deng X , Lu B , Cameron M , Fearns C , Patricelli MP , Yates JR 3rd , Gray NS , Lee JD (2010) Pharmacological inhibition of BMK1 suppresses tumor growth through promyelocytic leukemia protein. Cancer Cell 18: 258–267 20832753 10.1016/j.ccr.2010.08.008 PMC2939729

[emmm201505433-bib-0083] Zeisberg EM , Tarnavski O , Zeisberg M , Dorfman AL , McMullen JR , Gustafsson E , Chandraker A , Yuan X , Pu WT , Roberts AB *et al* (2007) Endothelial‐to‐mesenchymal transition contributes to cardiac fibrosis. Nat Med 13: 952–961 17660828 10.1038/nm1613

[emmm201505433-bib-0084] Zhou G , Hamik A , Nayak L , Tian H , Shi H , Lu Y , Sharma N , Liao X , Hale A , Boerboom L *et al* (2012) Endothelial Kruppel‐like factor 4 protects against atherothrombosis in mice. J Clin Invest 122: 4727–4731 23160196 10.1172/JCI66056 PMC3533563

[emmm201505433-bib-0085] Zhou Z , Rawnsley DR , Goddard LM , Pan W , Cao XJ , Jakus Z , Zheng H , Yang J , Arthur JS , Whitehead KJ *et al* (2015) The cerebral cavernous malformation pathway controls cardiac development via regulation of endocardial MEKK3 signaling and KLF expression. Dev Cell 32: 168–180 25625206 10.1016/j.devcel.2014.12.009 PMC4589864

