# Supplementary material for: KLF4 is a key determinant in the development and progression of cerebral cavernous malformations
Source: EMBO Mol Med. 2015 Nov 26;8(1):6–24. doi: 10.15252/emmm.201505433 (PMC4718159; doi:10.15252/emmm.201505433)
Supplement: Supplementary file 1 — Appendix [file EMMM-8-06-s001.pdf]

Table of contents

Appendix Figure S1
Appendix Figure S2
Appendix Figure S3
Appendix Figure S4
Appendix Table S1
Appendix Table S2
Appendix Figure Legends

Appendix Figure S1

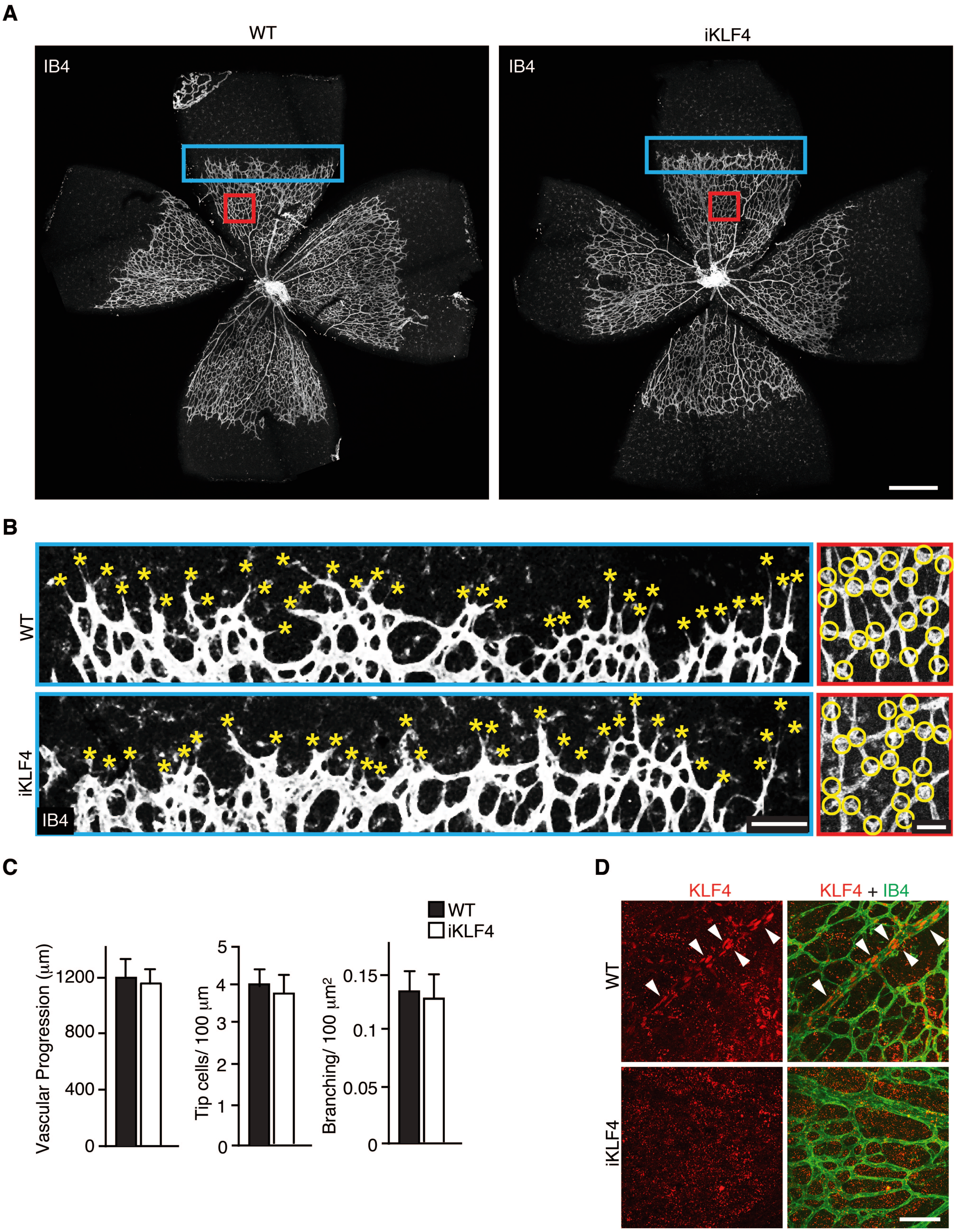

Appendix Figure S2

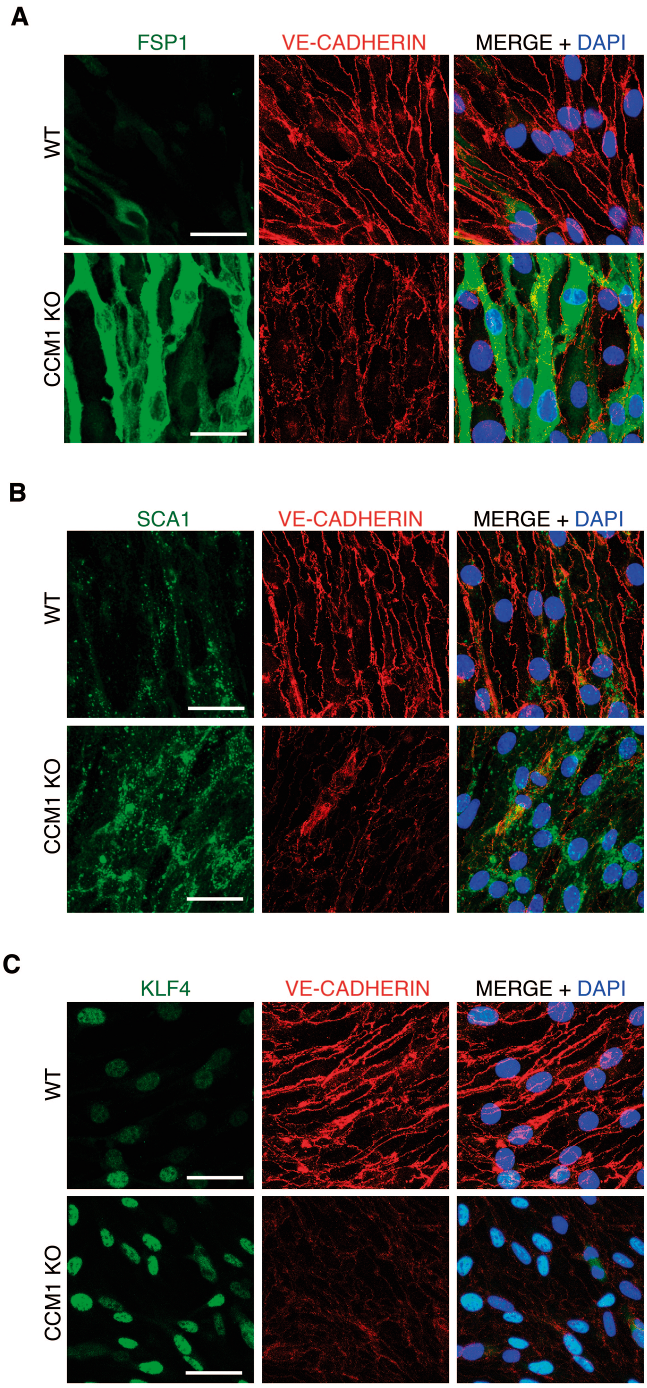

Appendix Figure S3

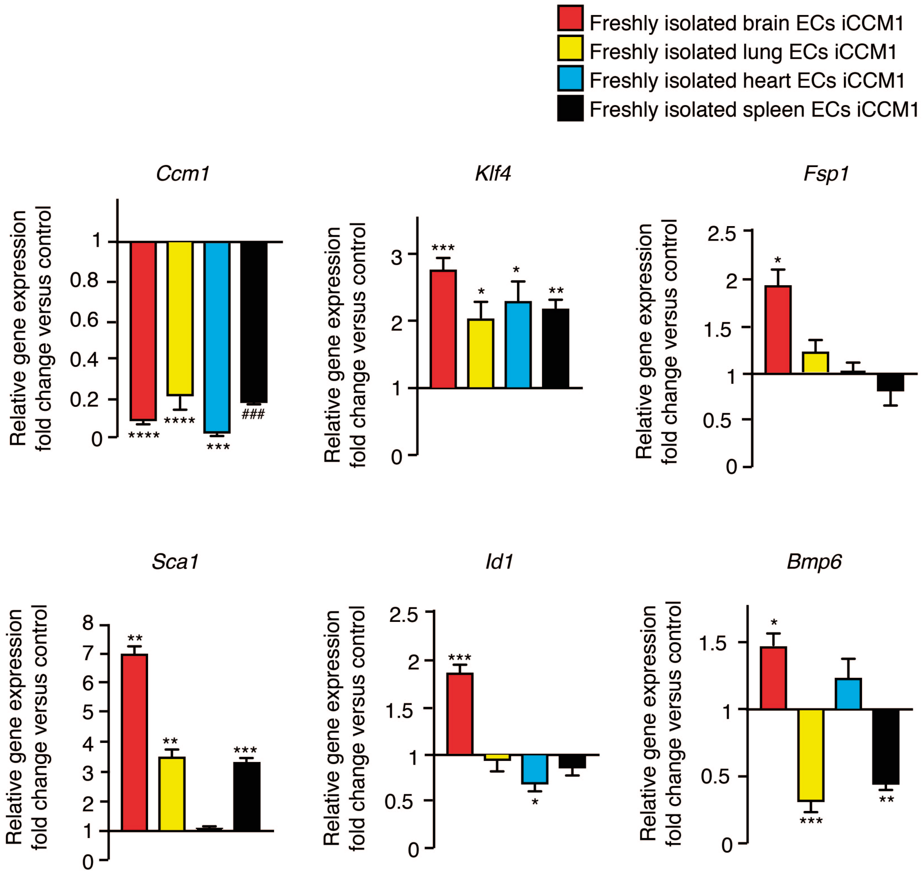

Appendix Figure S4

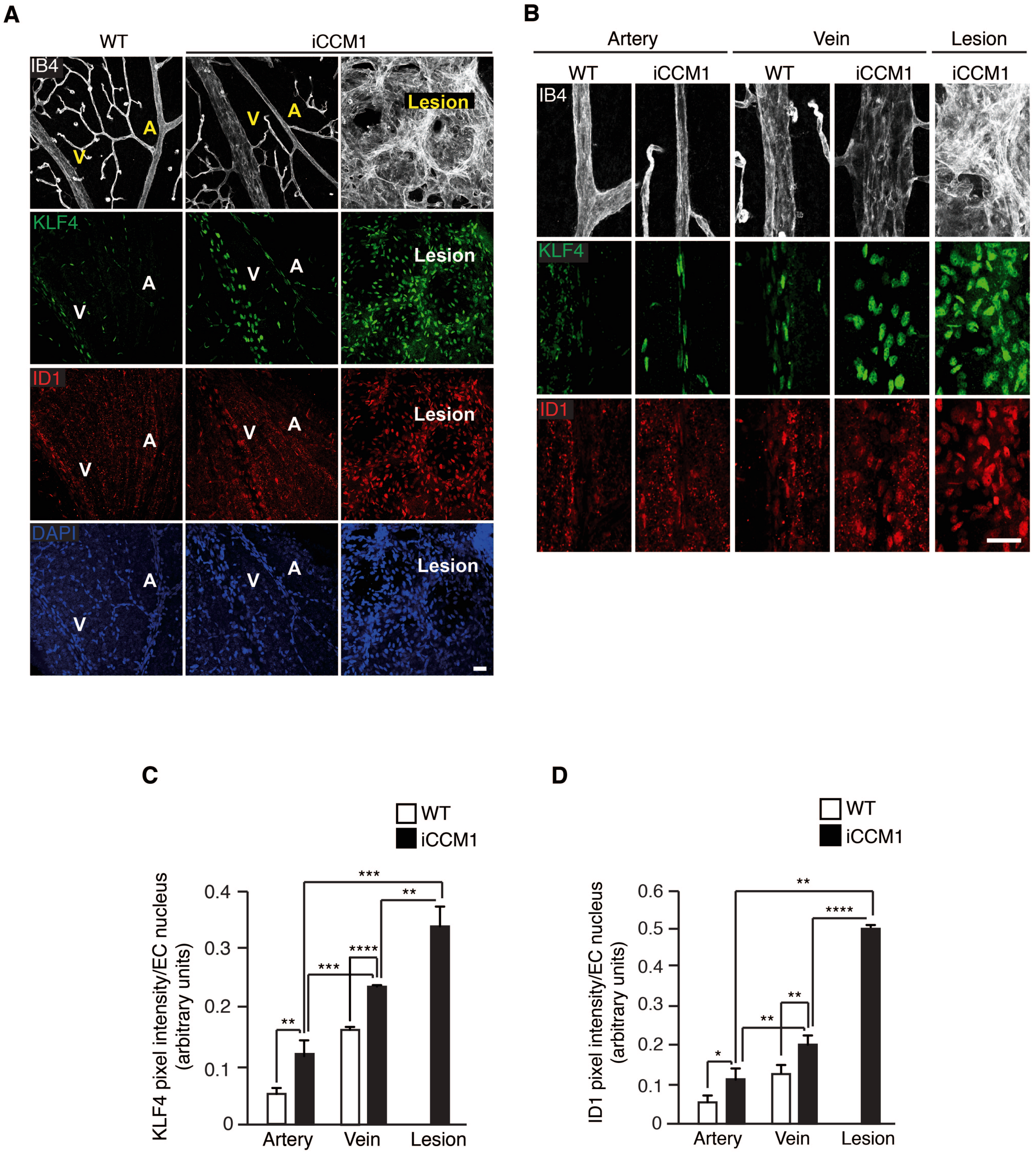

Appendix table S1- List of primers used in ChIP

Gene	Position	Forward	Reverse
BMP6	-5.0 KB	5′-TTTCTTTCTGGCCTCTGCAT-3′	5′-CCCTGAAGAGGTTGGTTCTG-3′
	-3.4 KB	5′-CACATCTGGCTTCTTGACGA-3′	5′-AGGTCTGCCTCCAGAGTTCA-3′
	-3.0 KB	5′-CCCCGAGTTTCACTTGACAT-3′	5′-GTGCACTCTCAACCTGGACA-3′
	-1.9 KB	5′-TGTTTCAGACCCCATAAACCA-3′	5′-GCTCAAATGGCCACTCATCT-3′
	-1.3 KB	5′-AACTCAAATGCAGCCCAAAC-3′	5′-TGGAATGTAGGCGTTTAGCA-3′
	-1.1 KB	5′-ACTCCTGATGCCTCCAAGAA-3′	5′-CAGCCAATGGAGAGGTGACT-3′
	+0.3 KB	5′-AATGACGACGAAGAGGATGG-3′	5′-AGACTCTTGCGGTTCAAGGA-3′
	+0.6 KB	5′-AGGATTTTGGGGGTAGCATC-3′	5′-GTGTTCCAGCTCCTCCCATT-3′
FSP1	-3.6KB	5′-TCTTCAAGCCCTGTGCTTCT-3′	5′-AATGGGGCTGAAGTGTCAAG-3′
	-3.1 KB	5′-CCAGCTATGCTTCTGGGGTA-3′	5′-GCGCCACTTCAGTTGCTATT-3′
	-2.2 KB	5′-CAGGTCTCCAAAAGGCAGAG-3′	5′-TCTACCACCTTCACCCCAAC-3′
	-2.0 KB	5′-GTTGGGGTGAAGGTGGTAGA-3′	5′-CTCCGACTGGCAGATCTTGT -3′
	-1.4 KB	5′-CCCAGTAGCCTCTGATCCAA-3′	5′-GGTGGGGTTAGCACATCAAG-3′
	-0.9 KB	5′-GGTGTCAGGCCCTGTAGAAA-3′	5′-ACTCTCCTTGCAGCATCTGG-3′
	+0.1 KB	5′-ACTGCTAGCGGCATTAGAGG-3′	5′-CTCCGGGAGCTCAGATGTAG-3′
	+0.7 KB	5′-GCAGGCATTCGTGTTTGTAG-3′	5′-AAAAACCCCAGCTGCCTAAT-3′
SCA1	-3.9 KB	5′-TGAGGGCCTTCATACCTCTG-3′	5′-GGTGAAGATGCCTGACTGCT-3′
	-3.3 KB	5′-GCATGACAGTGGTGAGATGG-3′	5′-CCCCCAAGAAAACAGACAGA-3′
	-1.4 KB	5′-GAAGGGAGGACTGTCACCTG-3′	5′-CACGGAGTCCCTCTTTCAGT-3′
	-0.9 KB	5′-ACCACTGTGCTGGGTTATGC-3′	5′- ACCCCACTTAAGCAGGGAAG-3′
	-0.4 KB	5′-GCCACTGCAAACCATACCTT -3′	5′-CTGGCTCCAACACACAGCTA-3′
	-0.1 KB	5′-AACATGATGGCCTGGAAAAG-3′	5′-GCACAGTGGCAAGTTCTGTC-3′
	+0.4 KB	5′-CCCTTCTCTGAGGATGGACA-3′	5′-AGGTGTACCCACAACCCTGA-3′
ID1	-4.9 KB	5′-AGAACTCTCCAGGAAGTGATGG-3′	5′-TCGAGACAGGGTTTCTCTGTG-3′
	-4.6 KB	5′-TCTTGCTGTTTCTAGGGCATC-3′	5′-CGGAAACAGCCTCAACATCT-3′
	-3.8 KB	5′-CACATACCCACCCTTCTGCT-3′	5′-AGCTTCATGTAGCCCATGCT-3′
	-3.5 KB	5′-CCTCTTGAAACAGCACAGCA-3′	5′-CAAGCCTGATGATTGACACC-3′
	-3.0 KB	5′-GCTCTGGATGTCCTGGAACT-3′	5′-AAAGGACTGGAGAGGCACTG-3′
	-2.0 KB	5′-AGAATGCTCCAGCCCAGTTT-3′	5′-GTTGGGGTGTGTGTTGAGG-3′
	-1.0 KB	5′-AGAATGCTCCAGCCCAGTTT-3′	5′-GGGCTGGTCTGTGTCAGC-3′
	+0.1 KB	5′-TCCCACACTCTGTTCTCAGC-3′	5′-TCCCACACTCTGTTCTCAGC-3′
	+0.5 KB	5′-TGAGGTCCGAGGCAGAGTAT-3′	5′-GAGACCCACTGGAAAGGACA-3′

Appendix table S2- List of primers used for promoter cloning

Gene	Forward	Reverse
BMP6	5′-ACCGCTCGAGCAAGAGGATGGGGAAGGCT-3′	5′-GGAAGATCTTCCAGCTCCTCCCATTGC-3′
FSP1	5′-CGGGGTACCCACTGCCACCCTAACTCCA-3′	5′-ACCGCTCGAGTGCCATGGTAACCGTTGAG-3′
SCA1	5′-CGGGGTACCTGATGGCCCCGTTGTCAT-3′	5′-ACCGCTCGAGACTGAGCTCCACGTGTCC-3′

Appendix Figure Legends

Appendix Figure S1. The vasculature of the retina is not altered in the absence of KLF4.

(A) Isolectin B4 staining (IB4, used to identify vasculature) on WT and iKLF4 retinae at P6. Images are representative of 5 mice for each genotype. Scale bar: 300 μm

(B-C) Quantification of several vascular parameters (vascular progression, number of tip cells and branching points) in the retinae from WT and iKLF4 mice at P6. Data are mean ± SD (n=5 for each genotype from 3 different litters).

(B) Higher magnification pictures of the blue and red boxed areas shown in A and used as representative images for quantification of both number of tip cells (indicated by yellow asterisks) and branching points (indicated by yellow circles) in the retinal vasculature of WT and iKLF4 animals. Scale bars: 30 μm (blue boxes) and 20 μm (red boxes).

(C) Quantification of the average distance covered by the growing vessels measured as vascular progression (left panel), number of tip cells in 100 μm at the leading edge of the plexus (middle panel) and number of branchings in 100 μm^2 area (right panel).

(D) Representative immunostaining (one out of three performed) for KLF4 (red) in the retinae of WT and iKLF4 mice to show gene ablation. Vasculature of the retina is stained with IB4 (green). Scale bar: 40 μm.

Appendix Figure S2. Co-expression of VE-CADHERIN and EndMT markers in CCM1 KO ECs.

(A) Confocal analysis of VE-CADHERIN (red) and FSP1 (green) in cultured lung derived WT and CCM1 KO ECs.

(B) Confocal microscopy of VE-CADHERIN (red) and SCA1 (green) in the cell lines reported in A.

(C) Immunofluorescence of VE-CADHERIN (red) and KLF4 (green) in the cell lines reported in A. These data are representative of three independent observations. Scale bar: 30 μm.

Appendix Figure S3. EndMT occurs specifically in the brain vasculature of iCCM1 mice.

qRT-PCR analysis of *Ccm1*, *Klf4*, *Fsp1, Sca1, Id1* and *Bmp6* in freshly isolated ECs from brain, lung, heart and spleen of WT and iCCM1 mice at P12. Fold changes are relative to matched WT animals. Data are expressed as fold change versus control WT animals and are means ± SD from at least three mice for group. The SD of the values obtained in control mice did not exceed 10% of the mean. A two-tailed unpaired *t*-test was performed comparing iCCM1 mice versus WT. *Ccm1*: ****P<0.00001, ***P=0.0001, $^{\#\#\#}$P=0.0005; *Klf4*: ***P=0.0008, *P=0.0490, **P=0.0050; *Fsp1*: *P=0.0366; *Sca1*: **P=0.005, ***P=0.0005; *Id1*: ***P=0.0009, *P=0.0217; *Bmp6*: *P=0.0175, ***P=0.0003, **P=0.0059

Appendix Figure S4. KLF4 amount is higher in veins than in arteries of the retinal vasculature in both WT and iCCM1 mice.

(A) Confocal analysis of isolectin B4 (IB4, white), KLF4 (green) and ID1 (red) in the retinae of WT and iCCM1 mice at p12. A:artery; V:vein. IB4 identifies the vasculature; DAPI visualizes nuclei. Images are representative of three mice for each genotype. Scale bar: 30 μm

(B) Higher magnification images of IB4 (white), KLF4 (green) and ID1 (red) in artery, vein and lesion of WT and iCCM1 mice retinae at p12. Scale bar: 30 μm.

(C) Quantification of the KLF4 pixel intensity per endothelial nucleus in retinal arteries, veins and lesions of WT and iCCM1 mice. Co-localization highlighter plug in of Image J has been used to identify KLF4 positive pixels co-localizing with DAPI (to exclude background). Mean intensity of co-localizing pixel per field has been calculated through Image J and normalized on the number of endothelial nuclei. Three different fields for three different samples for each group (arteries, veins and lesions) have been analysed. A two-tailed unpaired *t*-test was performed. WT artery vs iCCM1 artery **P=0.0036; WT vein vs iCCM1 vein ****P<0.0001; iCCM1 artery vs iCCM1 vein ***P=0.0004; iCCM1 artery vs iCCM1 lesion ***P=0.0002; iCCM1 vein vs iCCM1 lesion **P=0.0015

(D) Quantification of the ID1 pixel intensity per endothelial nucleus in retinal arteries, veins and lesion of WT and iCCM1 mice performed as described in C. A two-tailed unpaired *t*-test was performed. WT artery vs iCCM1 artery *P=0.0146; WT vein vs iCCM1 vein **P=0.0078;

iCCM1 artery vs iCCM1 vein **P=0.0078; iCCM1 artery vs iCCM1 lesion **P=0.0078; iCCM1 vein vs iCCM1 lesion ****P=2.6E-05
