# Supplementary material for: KLF4 is a key determinant in the development and progression of cerebral cavernous malformations
Source: EMBO Mol Med. 2015 Nov 26;8(1):6–24. doi: 10.15252/emmm.201505433 (PMC4718159; doi:10.15252/emmm.201505433)
Supplement: Supplementary file 2 — Expanded View Figures PDF [file EMMM-8-06-s002.pdf]

Expanded View Figures

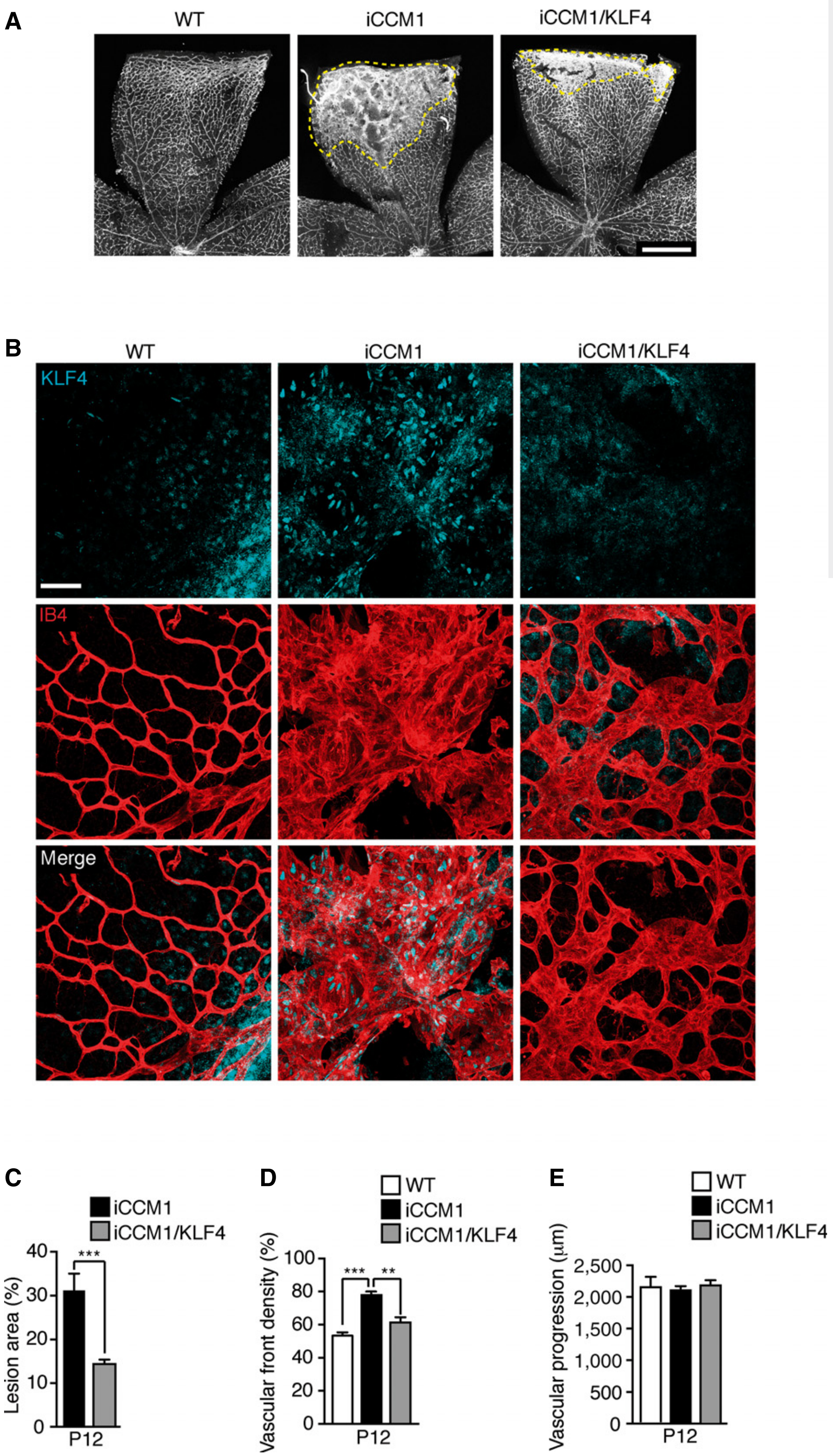

Figure EV1. ***Klf4* is critical for cavernoma development and progression in the retina.**

A Isolectin B4 staining (IB4, used to identify vasculature) on WT, iCCM1 and iCCM1/KLF4 retinae at P12. Dotted area highlights macroscopic differences in the extension of CCM lesion area between iCCM1 and iCCM1/KLF4 mice. Images are representative of five mice for each genotype. Scale bar: 500 µm.

B Representative immunostaining (one out of three performed, $n = 3$ in each group) for KLF4 (light blue) in the retinae of WT, iCCM1, and iCCM1/KLF4 mice. Vasculature at the periphery of the retina is shown after isolectin B4 staining (red). Scale bar: 60 µm.

C–E Quantification of percentage of retinal area covered by vascular lesions (C), vascular front density at the leading edge of the plexus (D), and average distance covered by the growing vessels measured as vascular progression (E) in retinae from WT, iCCM1, and iCCM1/KLF4 mice at P12. Data are mean ± SD ($n = 5$ for each genotype from three different litters). A two-tailed unpaired *t*-test was performed. ***$P = 0.0003$, **$P = 0.004$.

 © 2015 The Authors

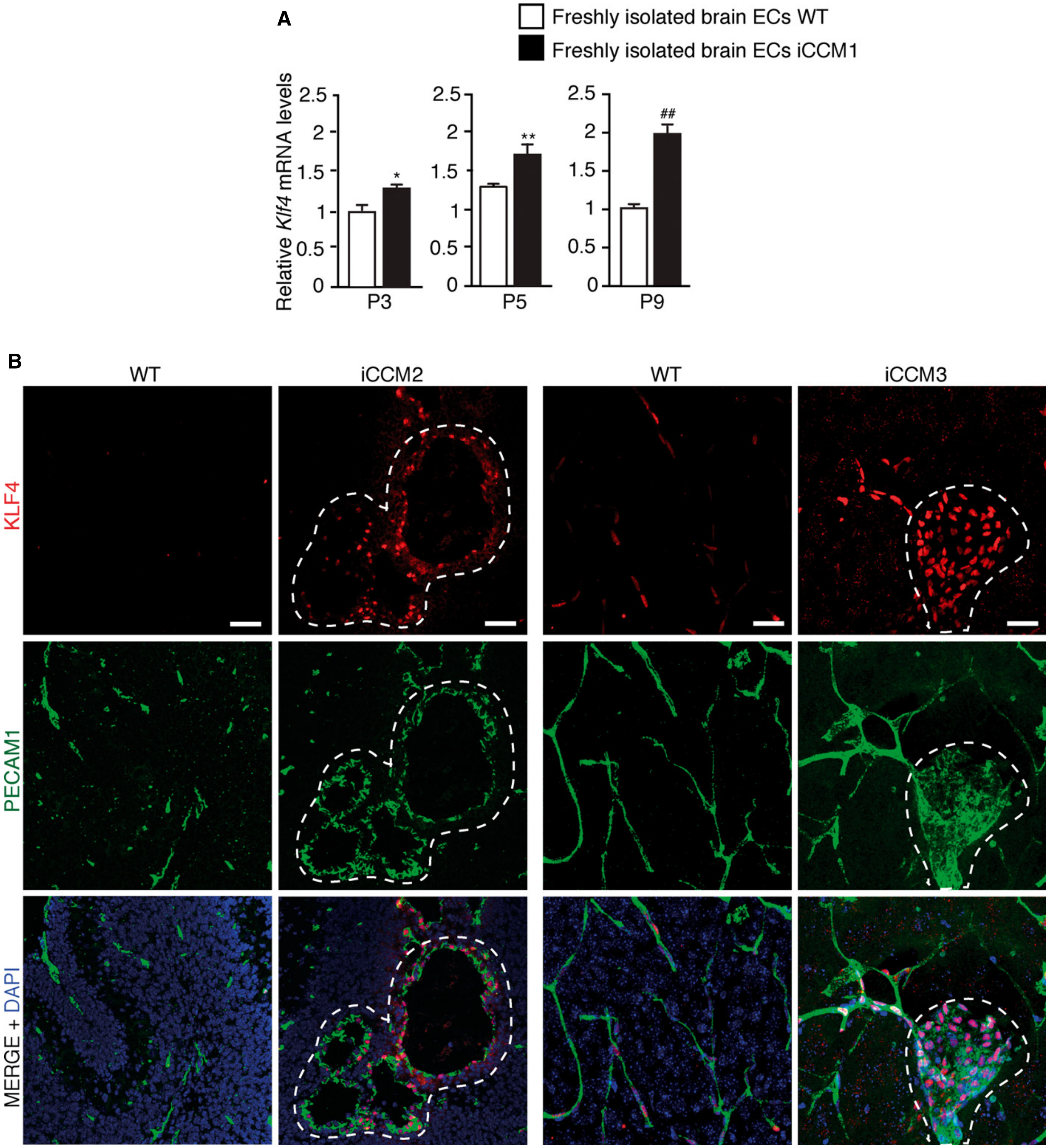

Figure EV2. KLF4 amount is increased upon loss of *Ccm1*, *Ccm2*, and *Ccm3*.

A qRT–PCR analysis of *Klf4* during disease progression at different times (P3, P5, and P9) after tamoxifen-induced *Ccm1* recombination (P1) in freshly isolated brain ECs derived from WT and iCCM1 mice. Data are mean ± SD (n = 3 in each group). Fold changes are relative to WT animals. A two-tailed unpaired t-test was performed. $^{*}P$ = 0.0143, $^{**}P$ = 0.002, $^{\#\#}P$ = 0.001.

B Representative immunostaining of KLF4 (red) in combination with PECAM1 (green, to identify ECs) in brain sections from iCCM2, iCCM3 mice, and their relative WT controls (one out of three performed). Cell nuclei are visualized with DAPI; dotted area highlights lesion area. Scale bars: 50 μm.

© 2015 The Authors

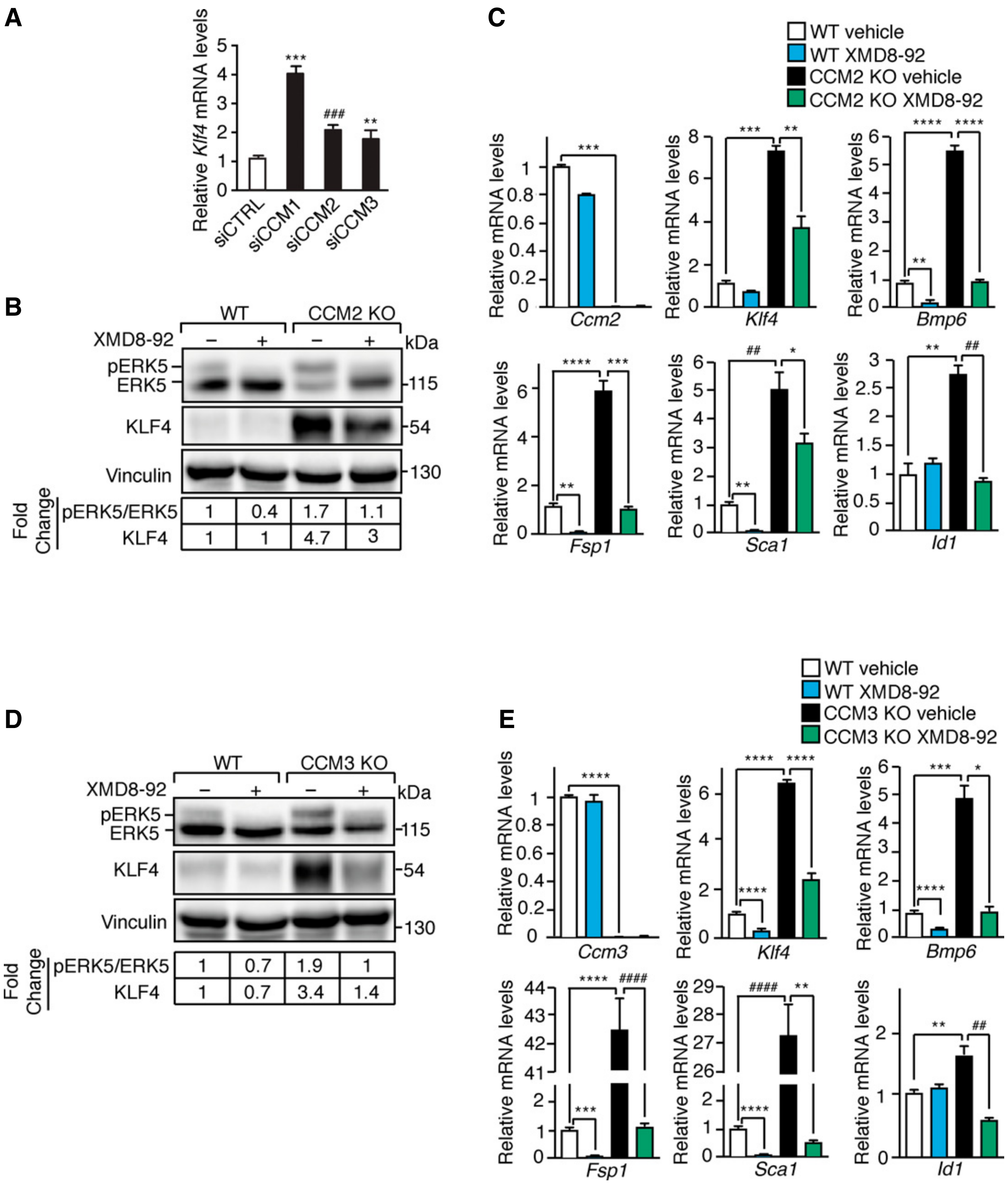

Figure EV3. Increased ERK5 phosphorylation is responsible for KLF4 upregulation and KLF4-dependent EndMT in the absence of both CCM2 and CCM3.

A qRT–PCR analysis of *Klf4* in WT ECs transfected with either siRNA directed to anyone of the three *Ccm* genes or control siRNA. Data are presented as mean ± SD (n = 3). Results are shown as fold changes relative to control siRNA-treated ECs. A two-tailed unpaired t-test was performed. ***P = 0.0001, ###P = 0.0009, **P = 0.0017.

B WB analysis of pERK5, ERK5, and KLF4 in lung-derived WT and CCM2 KO ECs treated with XMD8-92 or vehicle for 72 h. Both pERK5/ERK5 ratio and KLF4 amount normalized over vinculin, the loading control, were quantified by densitometry scan. WB results are representative of three independent observations.

C qRT–PCR of *Ccm2, Klf4, Bmp6*, and some EndMT markers in WT and CCM2 KO ECs treated with XMD8-92 or vehicle for 72 h. qRT–PCR results are shown as mean ± SD (n = 3), and fold changes are relative to vehicle-treated WT ECs. A two-tailed unpaired t-test was performed. *Ccm2*: ***P = 0.0003; *Klf4*: ***P = 0.0002, **P = 0.006; *Bmp6*: **P = 0.0018, ****P = 8.7E-05; *Fsp1*: **P = 0.0013, ****P = 6.5E-05, ***P = 0.0008; *Sca1*: **P = 0.001, ##P = 0.0025, *P = 0.029; *Id1*: **P = 0.0013, ##P = 0.0029.

D WB analysis of pERK5, ERK5, and KLF4 in lung-derived WT and CCM3 KO ECs treated with XMD8-92 or vehicle for 72 h. Both pERK5/ERK5 ratio and KLF4 were normalized and quantified as in (A).

E qRT–PCR of *Ccm3, Klf4, Bmp6*, and some EndMT markers in WT and CCM3 KO ECs treated with XMD8-92 or vehicle for 72 h quantified as in (C). *Ccm3*: ****P < 0.00001; *Klf4*: ****P < 0.00001; *Bmp6*: ****P < 0.00001, ***P = 0.0009, *P = 0.01; *Fsp1*: ***P = 0.0002, ****P < 0.00001, ####P = 3.8E-05; *Sca1*: ****P < 0.00001, ####P = 4.79E-05, **P = 0.0013; *Id1*: **P = 0.0039, ##P = 0.0017.

Source data are available online for this figure.

© 2015 The Authors

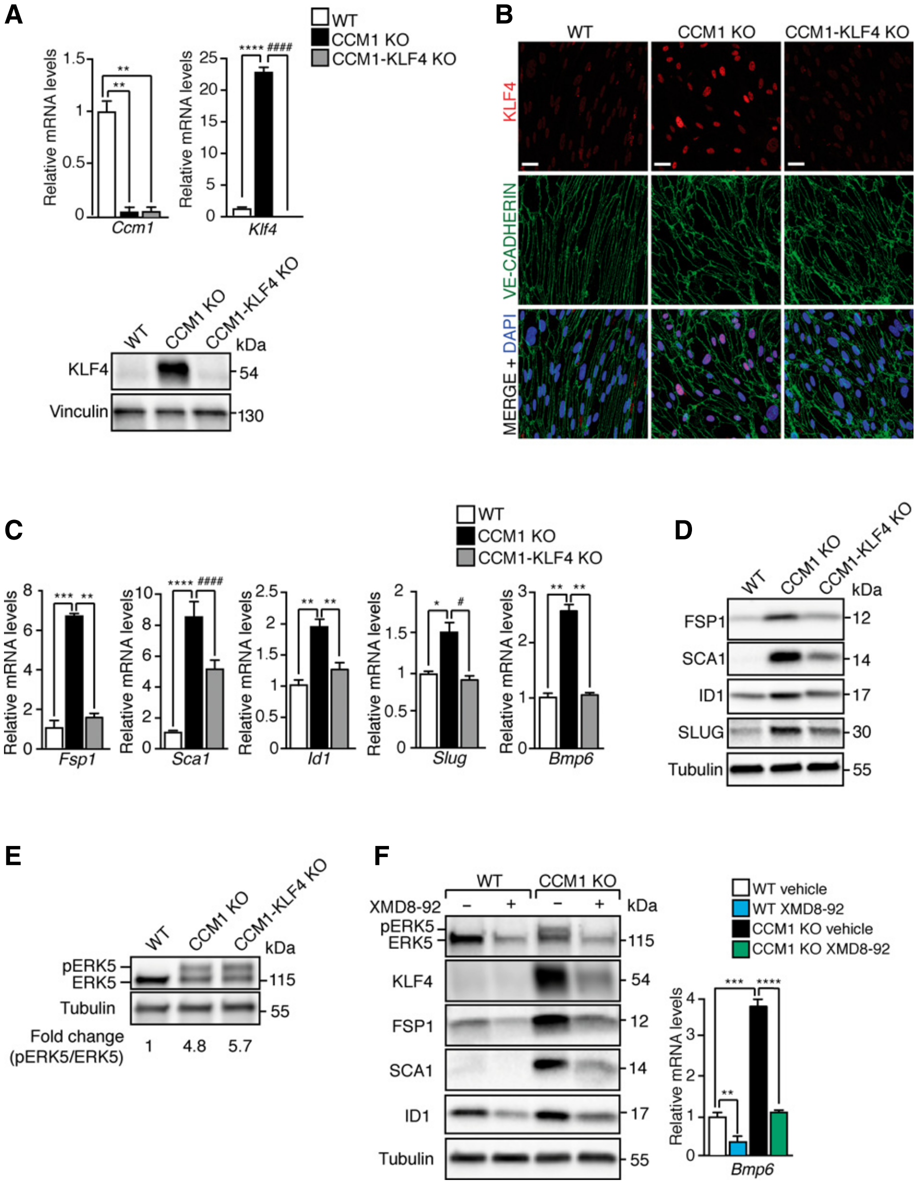

Figure EV4. ERK5-KLF4 axis regulates EndMT in primary brain CCM1 KO ECs.

A Upper panel: qRT–PCR of both *Ccm1* and *Klf4* gene recombination efficiency after *in vitro* TAT-Cre-recombinase treatment of primary brain ECs derived from WT, $Ccm1^{fl/fl}$, and $Ccm1^{fl/fl}/Klf4^{fl/fl}$ mice to originate cultured WT, CCM1 KO, and CCM1-KLF4 KO brain ECs, respectively (10 mice for each genotype per experimental replicate). Data are presented as mean ± SD ($n = 3$). Fold changes are relative to WT brain ECs. A two-tailed unpaired t-test was performed. *Ccm1*: **$P = 0.001$; *Klf4*: ****$P < 0.00001$, $^{\#\#\#\#}P = 5.2E-05$. Lower panel: WB of KLF4 in WT, CCM1 KO, and CCM1-KLF4 KO brain ECs described above. Vinculin is the loading control. These data are representative of three independent observations.

B Representative confocal analysis (out of three performed) of KLF4 (red) and VE-CADHERIN (green) in WT, CCM1 KO, and CCM1-KLF4 KO primary brain ECs obtained as in (A). Scale bar: 30 μm.

C qRT–PCR of EndMT markers in WT, CCM1 KO, and CCM1-KLF4 KO brain ECs obtained as in (A). Data are mean ± SD ($n = 3$). Fold changes are relative to WT ECs. A two-tailed unpaired t-test was performed. *Fsp1*: ***$P = 0.0006$, **$P = 0.0011$; *Sca1*: ****$P < 0.00001$, $^{\#\#\#\#}P = 7.7E-05$; *Id1*: **$P = 0.0017$; *Slug*: *$P = 0.02$, $^{\#}P = 0.01$; *Bmp6*: **$P = 0.003$.

D WB of FSP1, SCA1, ID1, and SLUG in WT, CCM1 KO, and CCM1-KLF4 KO brain ECs obtained as described in (A). Tubulin is the loading control. These data are representative of three independent observations.

E Analysis of pERK5 and ERK5 protein levels in WT, CCM1 KO, and CCM1-KLF4 KO brain ECs obtained as in (A). pERK5/ERK5 ratio normalized over tubulin is indicated.

F WB analysis of pERK5, ERK5, KLF4, FSP1, SCA1, ID1 (left panel), and qRT–PCR of *Bmp6* (right panel) in WT and CCM1 KO brain ECs, obtained as in (A), and then treated with XMD8-92 or vehicle for 72 h. qRT–PCR results are shown as mean ± SD ($n = 3$) and fold changes are relative to vehicle-treated WT ECs. A two-tailed unpaired t-test was performed. **$P = 0.001$, ***$P = 0.0006$, ****$P = 6.3E-05$. WB results are representative of three independent observations, and tubulin is the loading control.

Source data are available online for this figure.

© 2015 The Authors

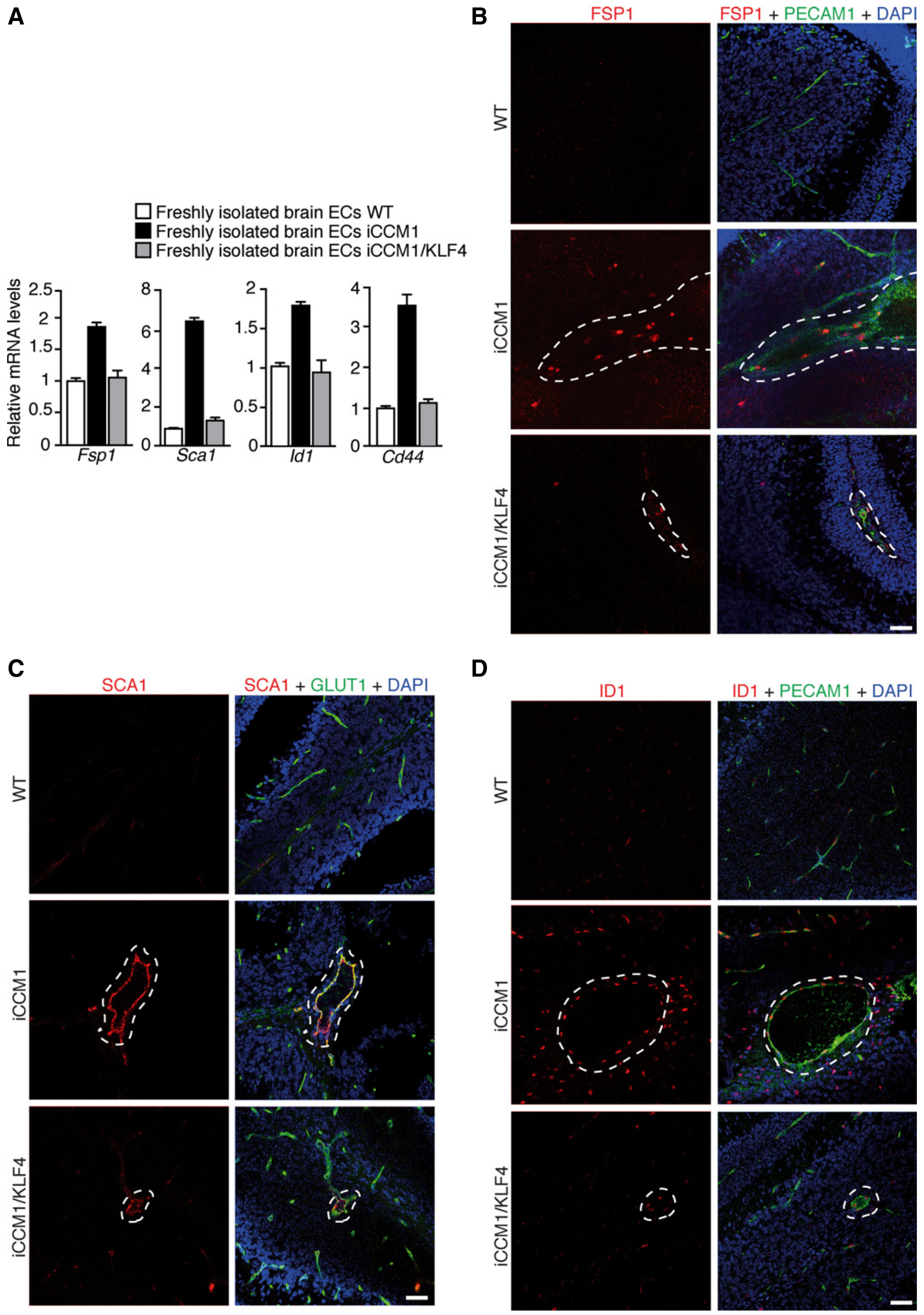

Figure EV5. EndMT marker expression is reduced in the absence of KLF4 *ex vivo* and *in vivo*.

A qRT–PCR of some EndMT markers in freshly isolated brain ECs from WT, iCCM1, and iCCM1/KLF4 mice analyzed at P12. Fold changes are relative to WT animals. Data are mean ± SD from a representative experiment out of three.

B–D Representative confocal analysis of (B) PECAM1 (green) and FSP1 (red), (C) GLUT1 (green) and SCA1 (red), or (D) PECAM1 (green) and ID1 (red) in normal cerebellar vessels of WT mice and vascular lesions (dotted area) of both iCCM1 and iCCM1/KLF4 mice (n = 4 in each group). PECAM1 identifies ECs; DAPI visualizes nuclei. Scale bars: 50 μm.

 © 2015 The Authors
