# Supplementary figures and images for: KLF4 is a key determinant in the development and progression of cerebral cavernous malformations
Source: EMBO Mol Med. 2015 Nov 26;8(1):6–24. doi: 10.15252/emmm.201505433 (PMC4718159; doi:10.15252/emmm.201505433)

Source data Figure EV3

Panel B

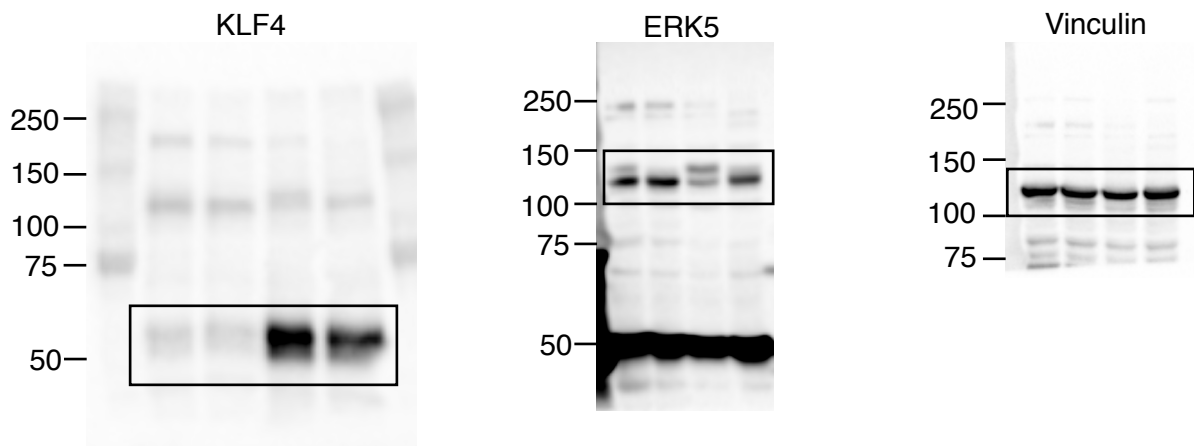

Panel D

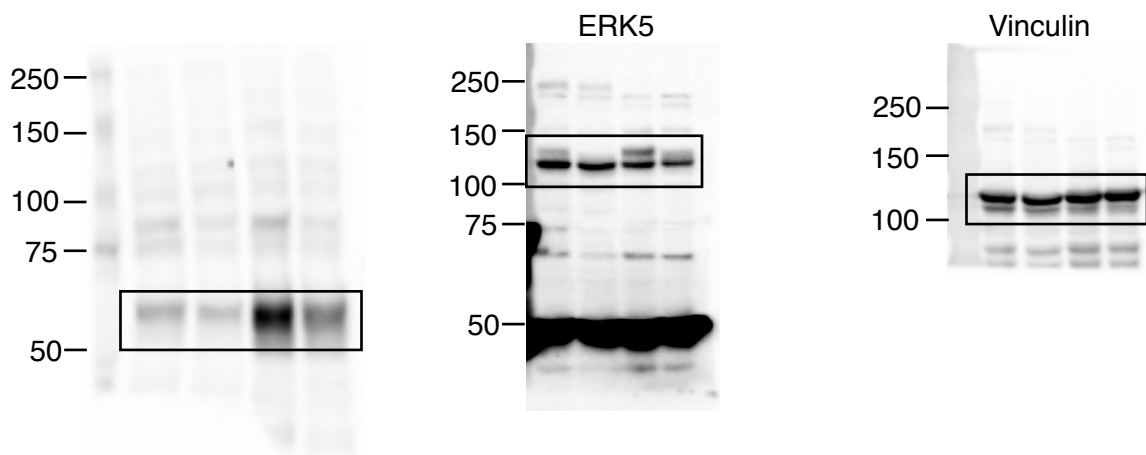

Supplement: Supplementary file 3 — Source Data for Expanded View and Appendix [file EMMM-8-06-s010.zip › Source_data_for_EV_and_Appendix/Source data contained in Figure EV3.pdf]

## Source data Figure EV4

### Panel A

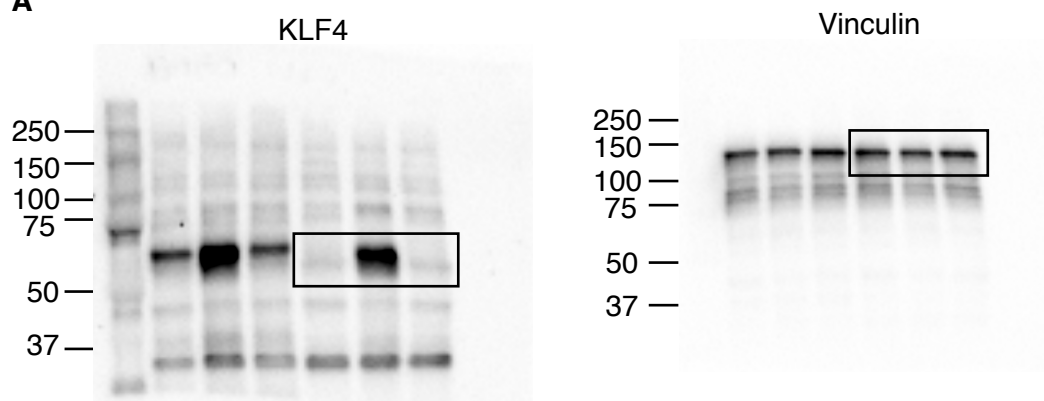

### Panel D

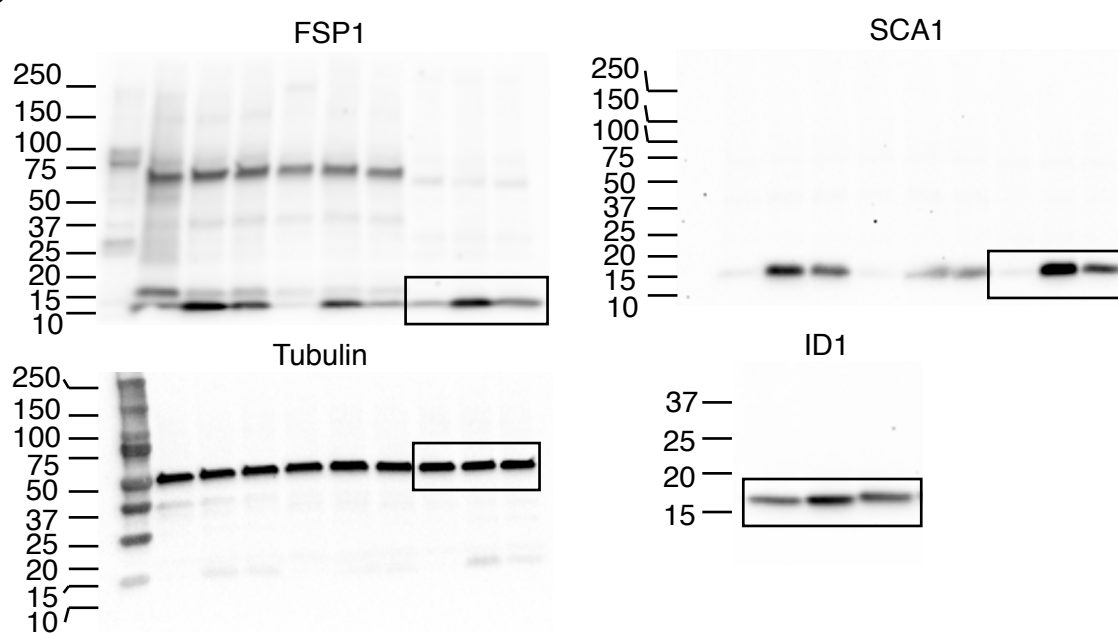

### Panel F

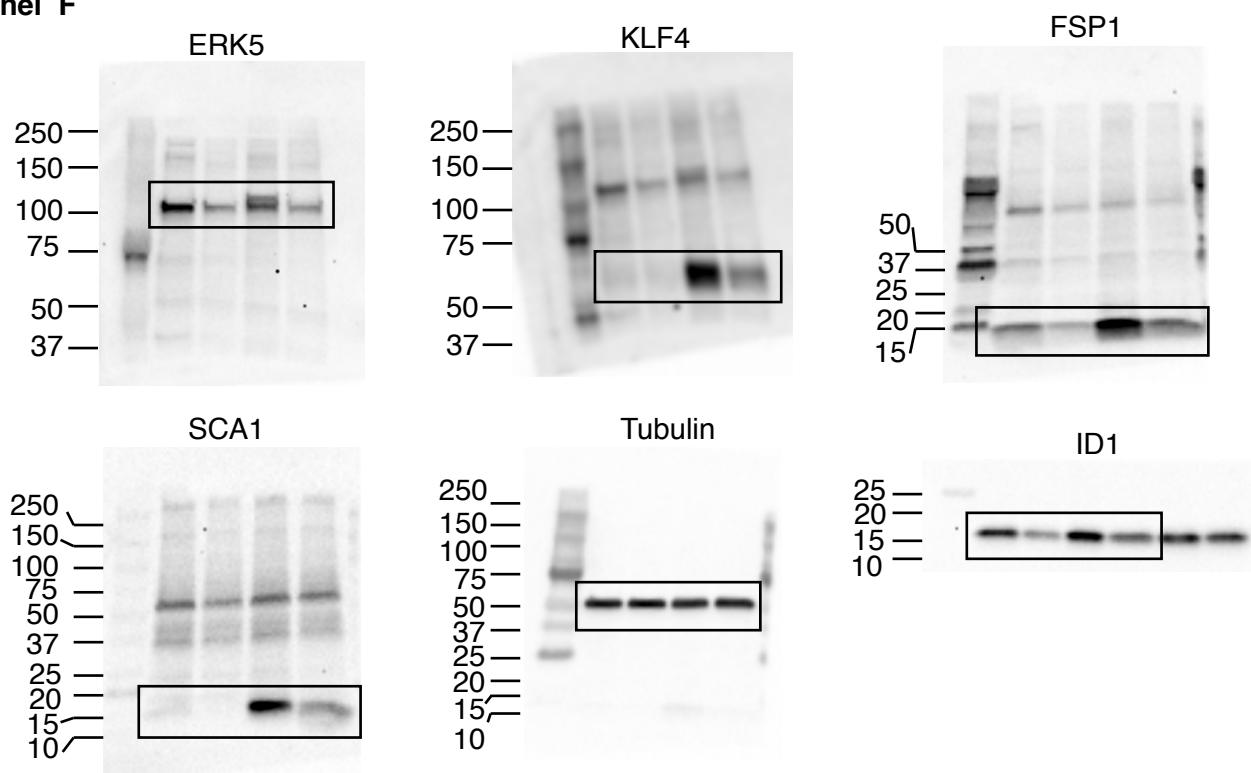

Supplement: Supplementary file 3 — Source Data for Expanded View and Appendix [file EMMM-8-06-s010.zip › Source_data_for_EV_and_Appendix/Source data Figure EV4.pdf]

## Source data Figure 2

### Panel A

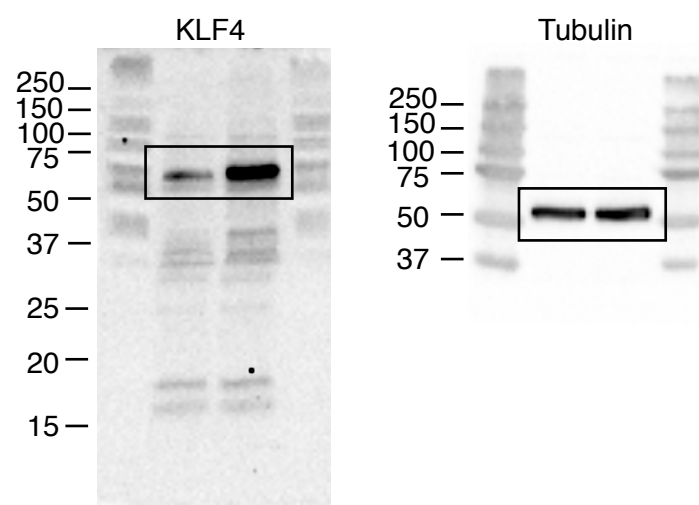

Supplement: Supplementary file 5 — Source Data for Figure 2 [file EMMM-8-06-s003.pdf]

Source data Figure 3

Panel B

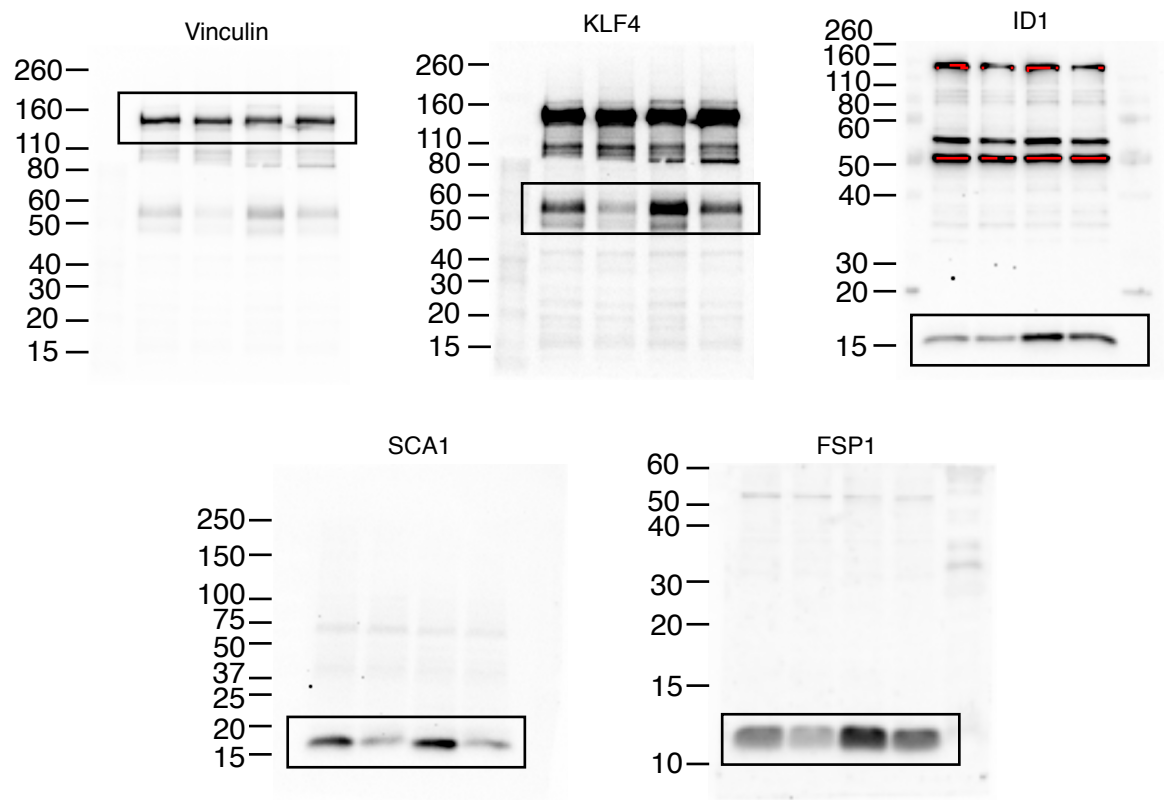

Panel E

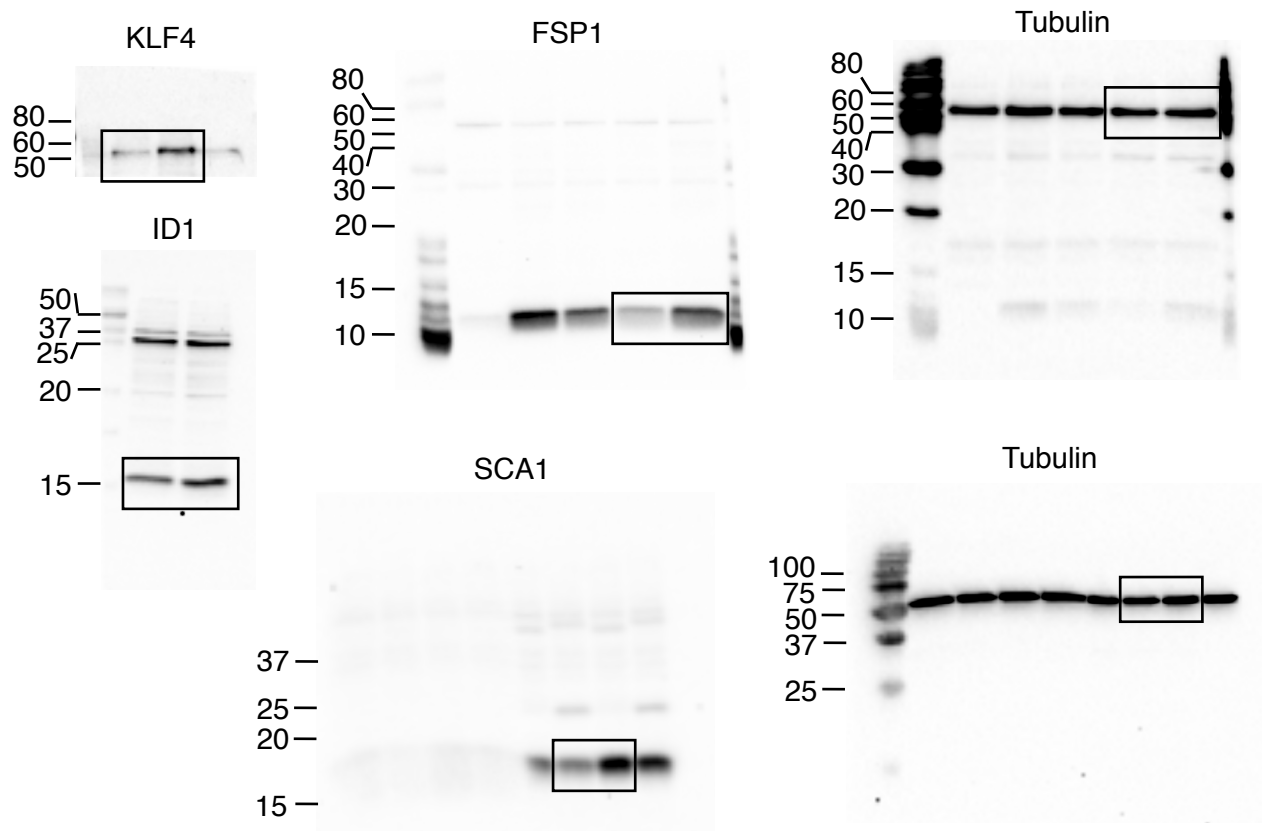

Supplement: Supplementary file 6 — Source Data for Figure 3 [file EMMM-8-06-s004.pdf]

**Source data Figure 4**

**Panel B**

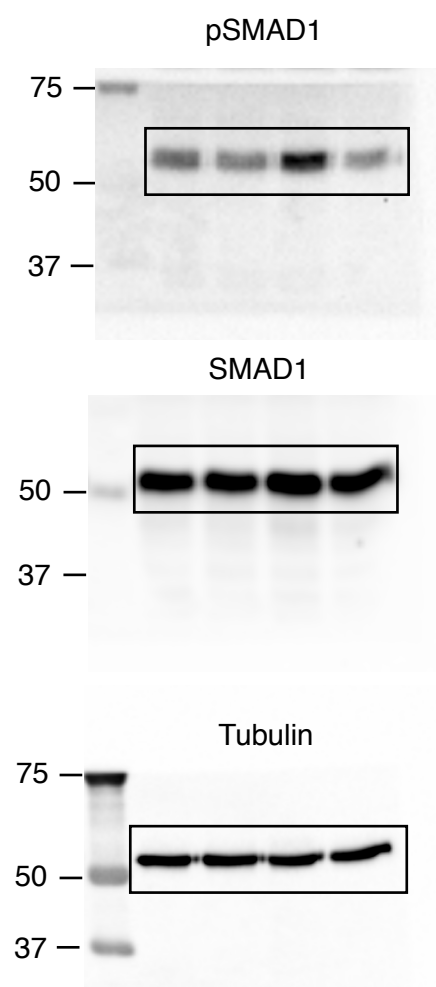

Supplement: Supplementary file 7 — Source Data for Figure 4 [file EMMM-8-06-s005.pdf]

Source data Figure 5

Panel B

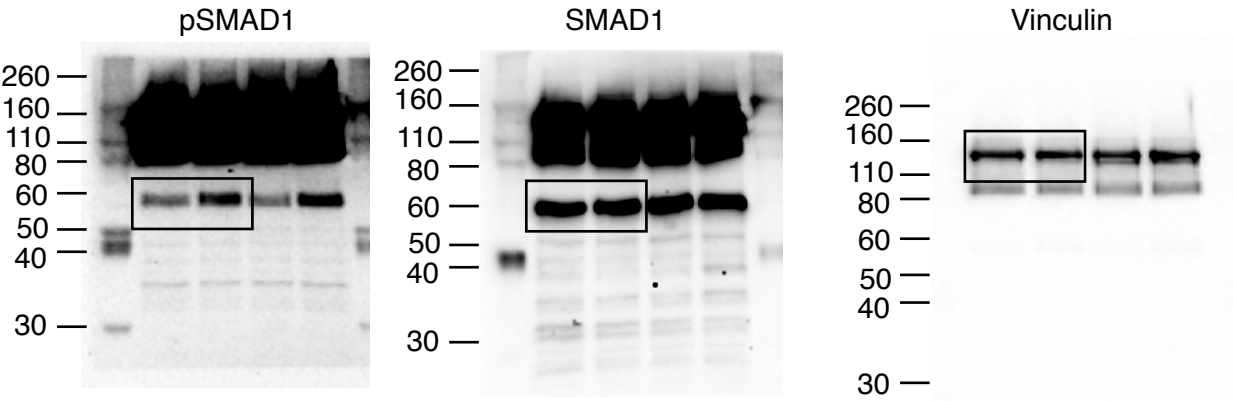

Panel E

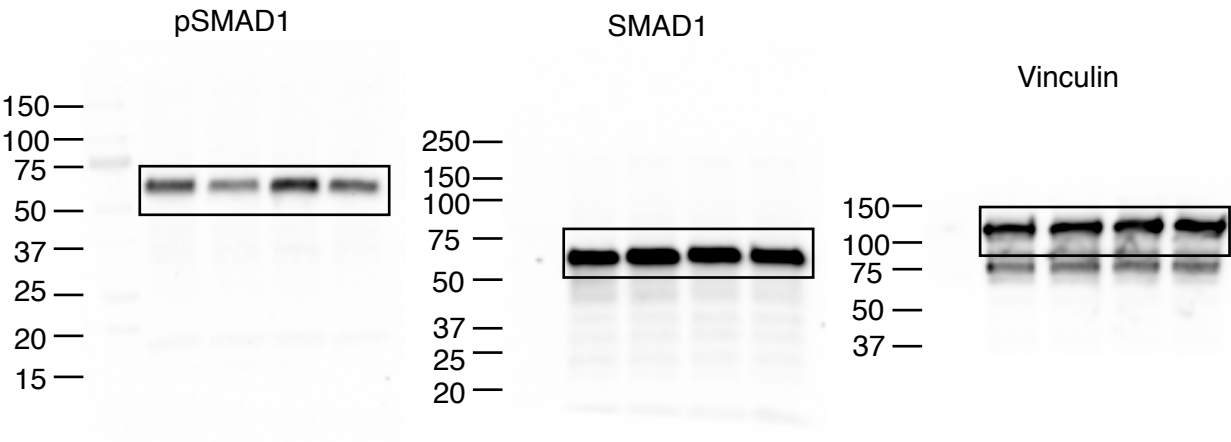

Panel F

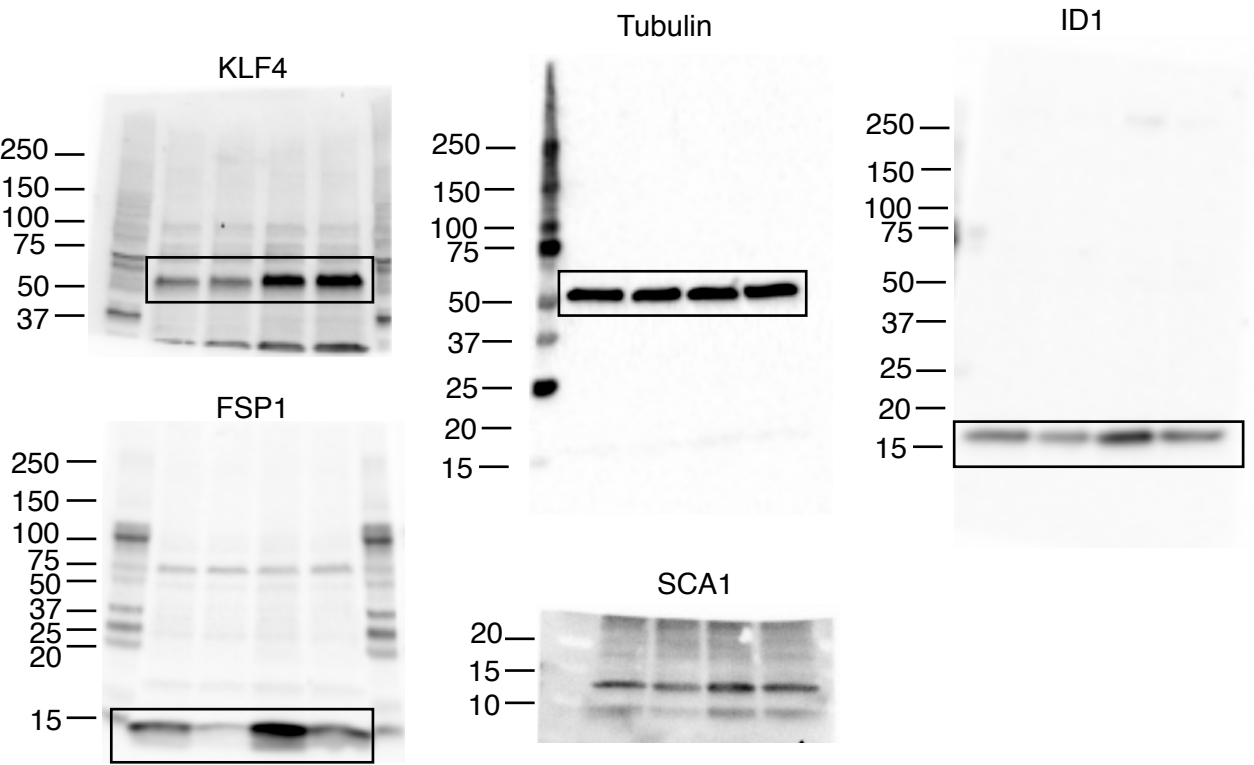

Supplement: Supplementary file 8 — Source Data for Figure 5 [file EMMM-8-06-s006.pdf]

Source data Figure 6

Panel A

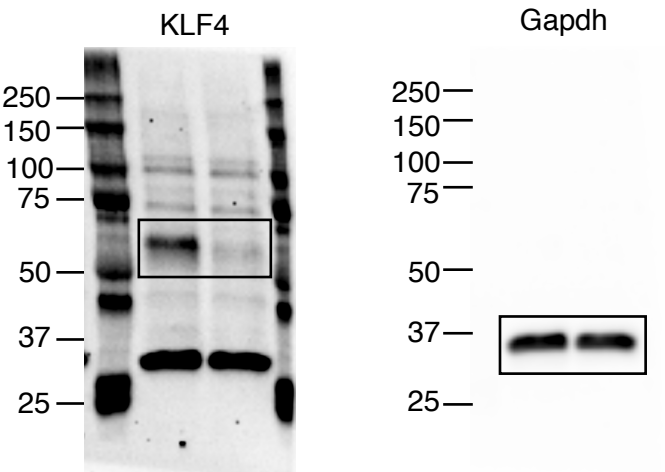

Panel B

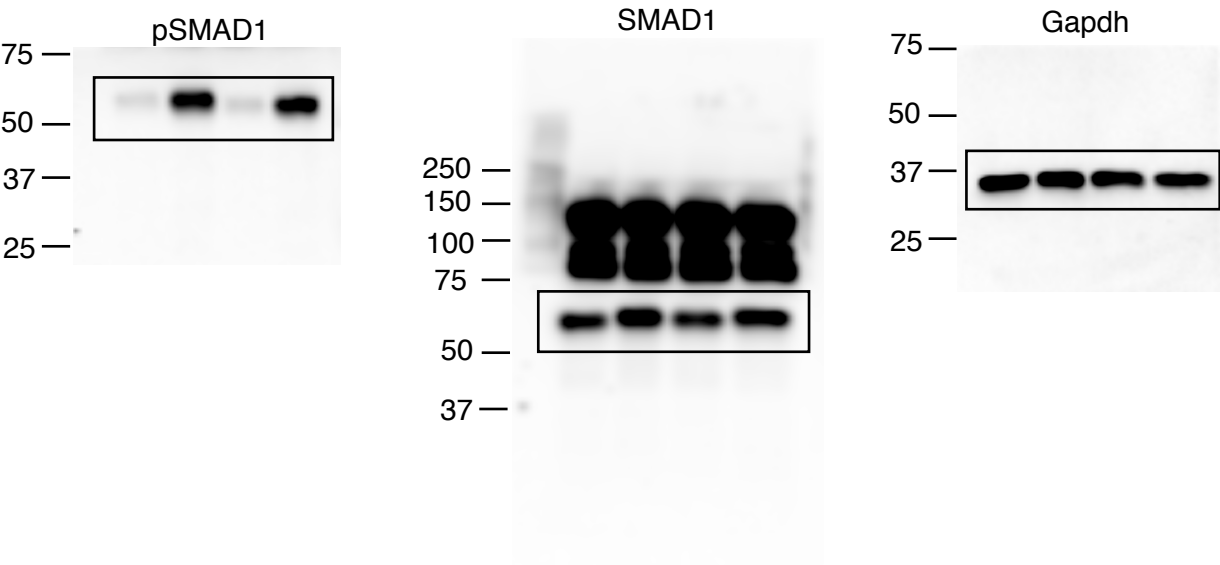

Supplement: Supplementary file 9 — Source Data for Figure 6 [file EMMM-8-06-s007.pdf]

Source data Figure 8

Panel A

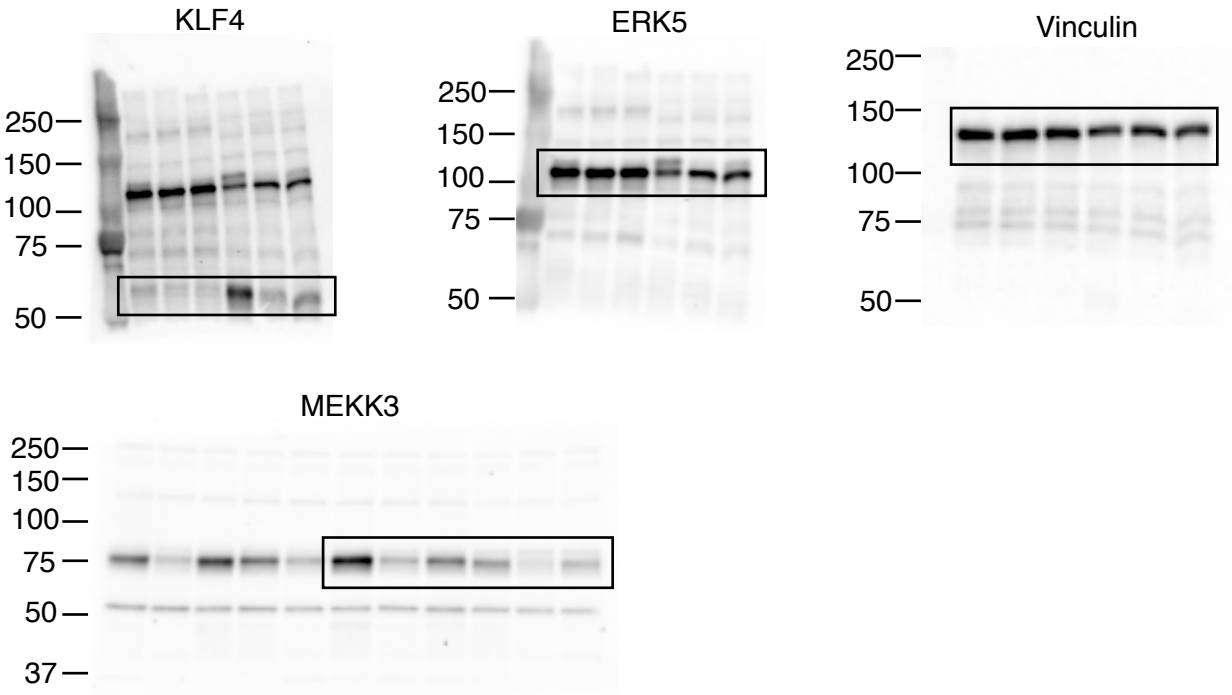

Panel B

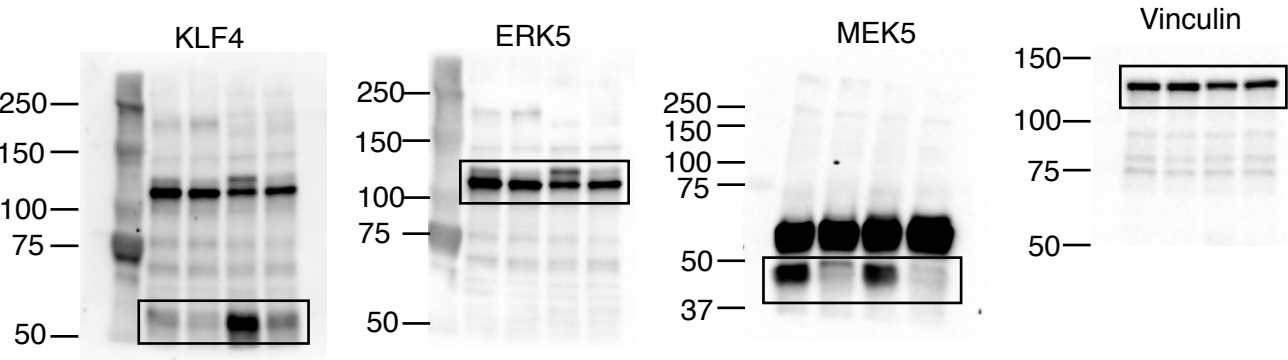

Panel C

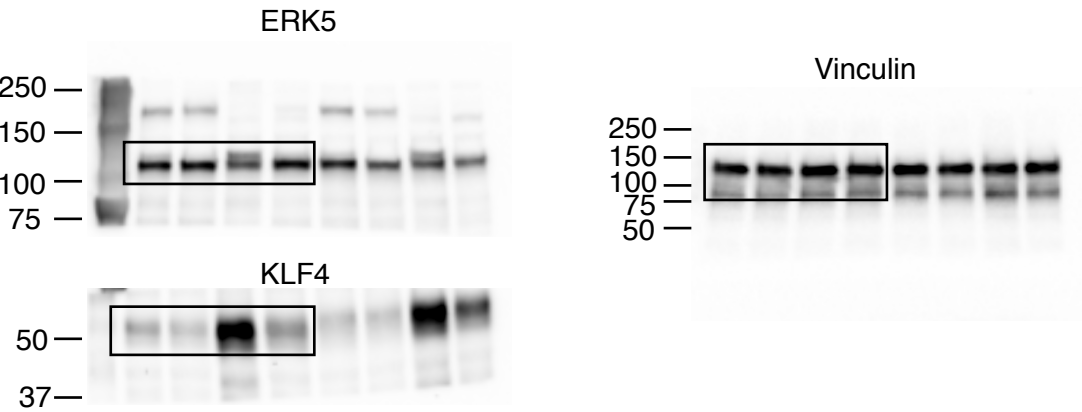

Supplement: Supplementary file 11 — Source Data for Figure 8 [file EMMM-8-06-s009.pdf]
