# Supplementary material for: KLF4 is a key determinant in the development and progression of cerebral cavernous malformations
Source: EMBO Mol Med. 2015 Nov 26;8(1):6–24. doi: 10.15252/emmm.201505433 (PMC4718159; doi:10.15252/emmm.201505433)

**Source data Figure 7 (panels A,B,C,D)**

**Panel A**

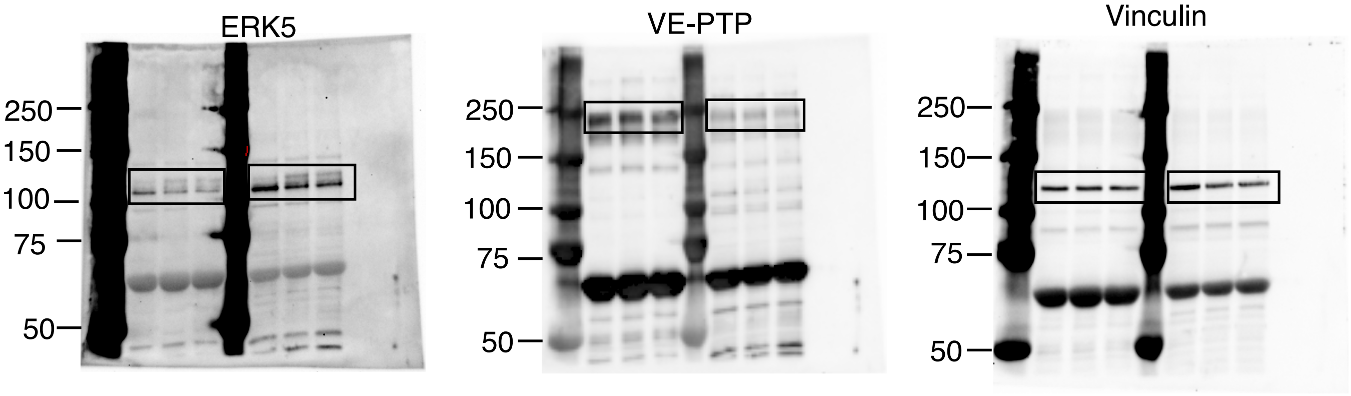


**Panel B**

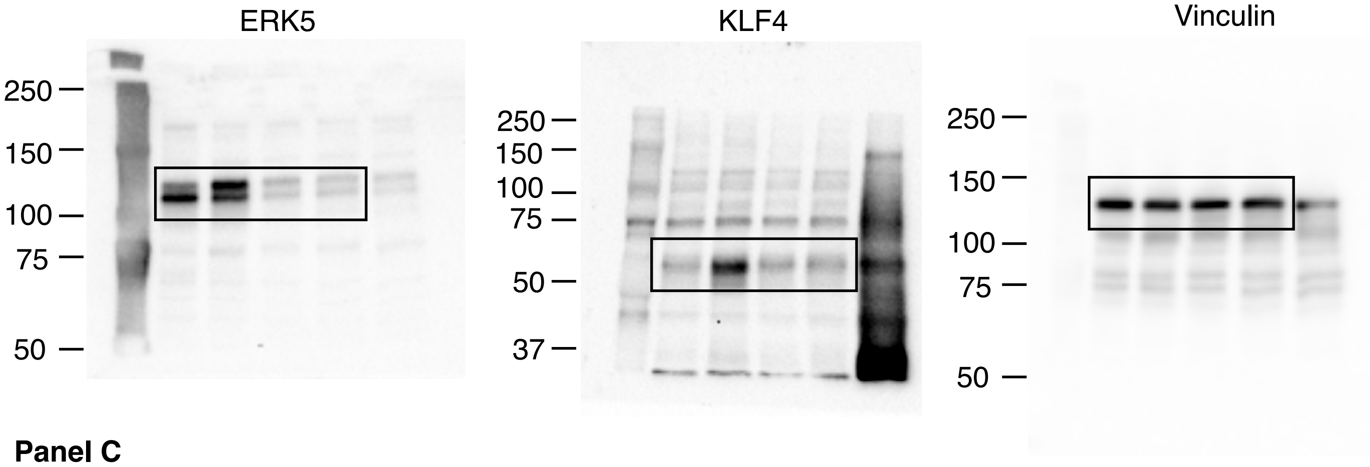


**Panel C**

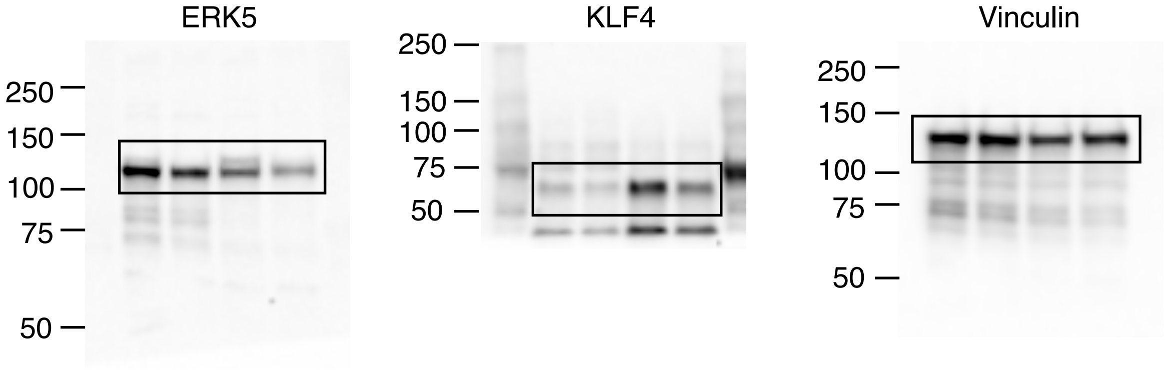


**Panel D**

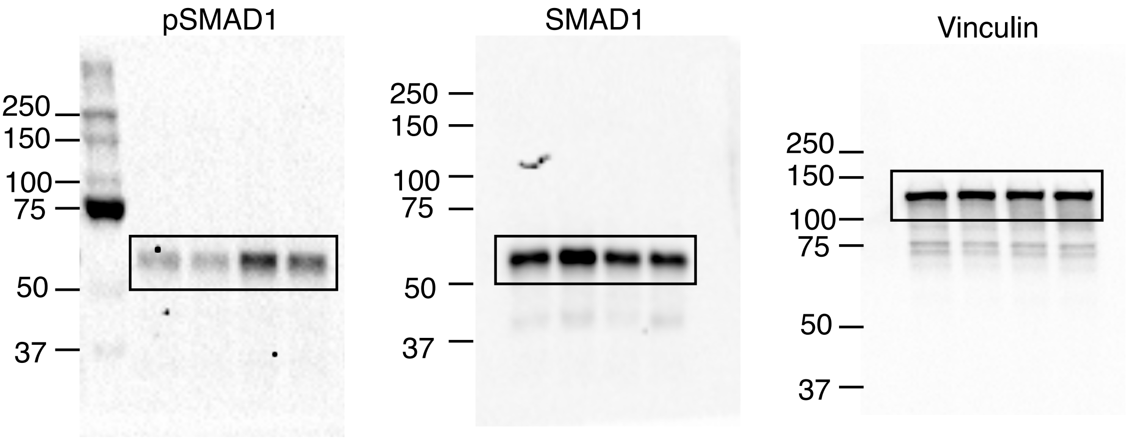

**Source data Figure 7 (panels E, F)**

**Panel E**

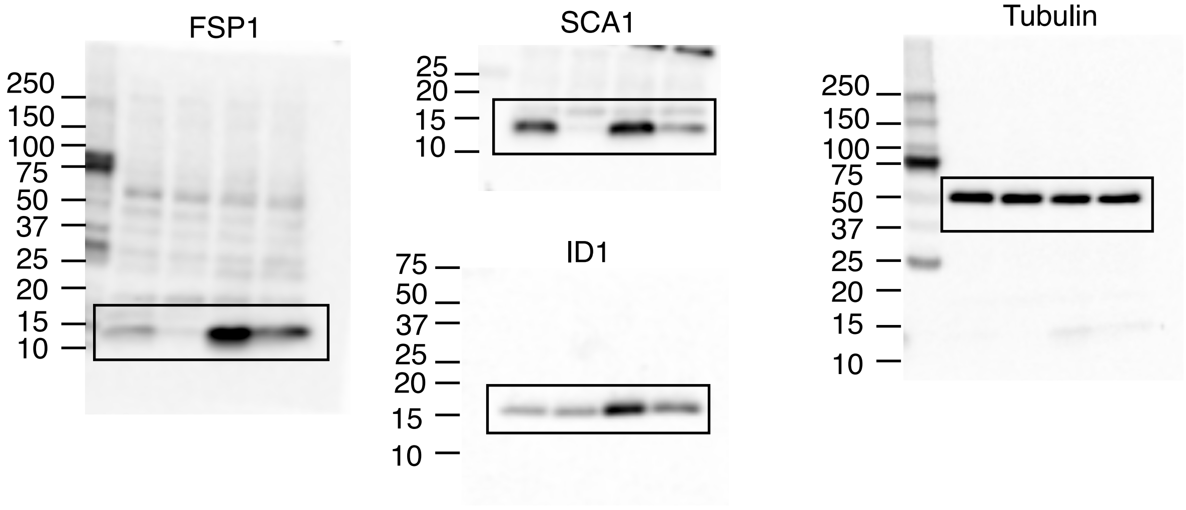


**Panel F**

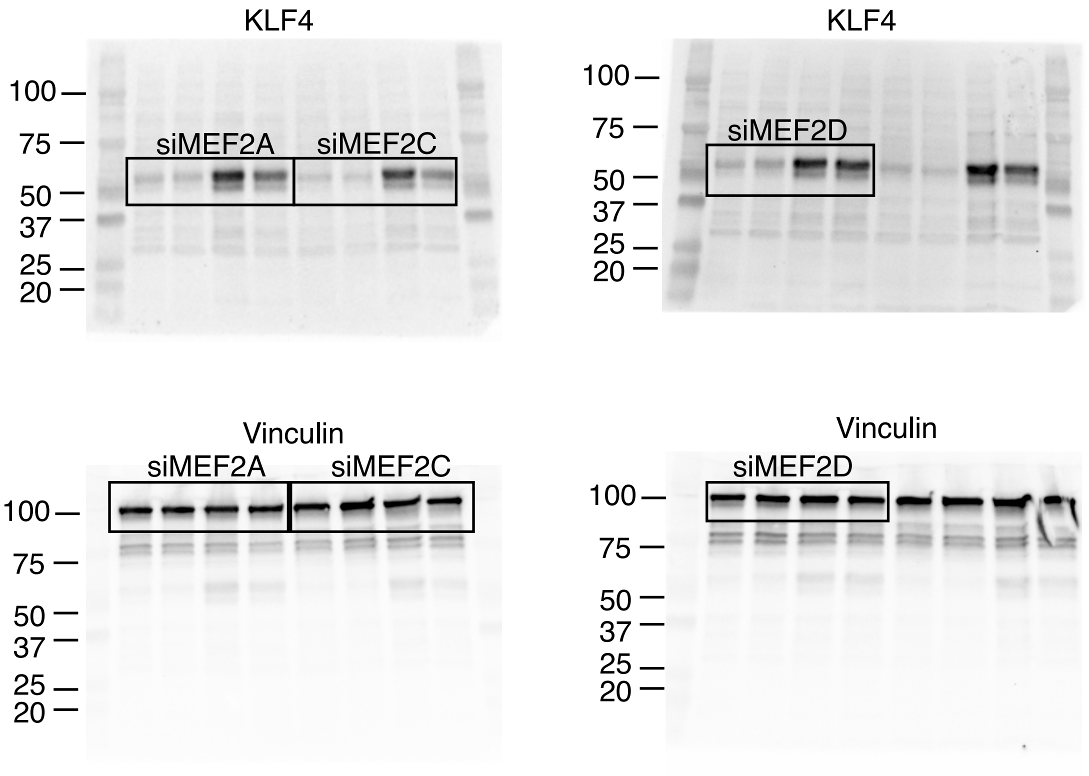

Supplement: Supplementary file 10 — Source Data for Figure 7 [file EMMM-8-06-s008.pdf]
