# Supplementary material for: KLF4 is a key determinant in the development and progression of cerebral cavernous malformations
Source: EMBO Mol Med. 2015 Nov 26;8(1):6–24. doi: 10.15252/emmm.201505433 (PMC4718159; doi:10.15252/emmm.201505433)
Supplement: Supplementary file 4 — Review Process File [file EMMM-8-06-s011.pdf]

EMBO Molecular Medicine

KLF4 is a key determinant in the development and progression of Cerebral Cavernous Malformations

Roberto Cuttano, Noemi Rudini, Luca Bravi, Monica Corada, Costanza Giampietro, Eleanna Papa, Marco Francesco Morini, Luigi Maddaluno, Nicolas Baeyens, Ralf H. Adams, Mukesh K. Jain, Gary K. Owens, Martin Schwartz, Maria Grazia Lampugnani and Elisabetta Dejana

Corresponding author: Elisabetta Dejana, IFOM-FIRC Institute of Molecular Oncology

Review timeline:

Submission date:	12 May 2015
Accepted:	23 October 2015

Editor: Roberto Buccione

Transaction Report:

No Review Process File is available with this article, as the authors have chosen not to make the review process public in this case

© EMBO
